# Neoadjuvant chemotherapy with or without camrelizumab in resectable esophageal squamous cell carcinoma: the randomized phase 3 ESCORT-NEO/NCCES01 trial

**DOI:** 10.1038/s41591-024-03064-w

**Published:** 2024-07-02

**Authors:** Jianjun Qin, Liyan Xue, Anlin Hao, Xiaofeng Guo, Tao Jiang, Yunfeng Ni, Shuoyan Liu, Yujie Chen, Hongjing Jiang, Chen Zhang, Mingqiang Kang, Jihong Lin, Hecheng Li, Chengqiang Li, Hui Tian, Lin Li, Junke Fu, Yong Zhang, Jianqun Ma, Xiaoyuan Wang, Maoyong Fu, Hao Yang, Zhaoyang Yang, Yongtao Han, Longqi Chen, Lijie Tan, Tianyang Dai, Yongde Liao, Weiguo Zhang, Bin Li, Qixun Chen, Shiping Guo, Yu Qi, Li Wei, Zhigang Li, Ziqiang Tian, Xiaozheng Kang, Ruixiang Zhang, Yong Li, Zhen Wang, Xiankai Chen, Zhiguo Hou, Rongrong Zheng, Wenqing Zhu, Jie He, Yin Li

**Affiliations:** 1 Section of Esophageal and Mediastinal Oncology, Department of Thoracic Surgery, National Cancer Center/National Clinical Research Center for Cancer/Cancer Hospital, Chinese Academy of Medical Sciences and Peking Union Medical College, Beijing, China; 2 Department of Pathology, National Cancer Center/National Clinical Research Center for Cancer/Cancer Hospital, Chinese Academy of Medical Sciences and Peking Union Medical College, Beijing, China; 3 Department of Thoracic Surgery, Anyang Cancer Hospital, Anyang, China; 4 Department of Thoracic Surgery, Tangdu Hospital, Air Force Military Medical University, Xi’an, China; 5 Department of Thoracic Surgery, Fujian Provincial Cancer Hospital, Fuzhou, China; 6 Department of Esophageal Minimal Invasive Surgery, Tianjin Medical University Cancer Institute and Hospital, Tianjin, China; 7 Department of Thoracic Surgery, Fujian Medical University Union Hospital, Fuzhou, China; 8 Department of Thoracic Surgery, Ruijin Hospital, Shanghai Jiao Tong University School of Medicine, Shanghai, China; 9 Department of Thoracic Surgery, Qilu Hospital of Shandong University, Jinan, China; 10 Department of Thoracic Surgery, The First Affiliated Hospital of Xi’an Jiaotong University, Xi’an, China; 11 Department of Thoracic Surgery, Harbin Medical University Cancer Hospital, Harbin, China; 12 Department of Thoracic Surgery, Affiliated Hospital of North Sichuan Medical College, Nanchong, China; 13 Department of Thoracic Surgery, Sichuan Cancer Hospital, Chengdu, China; 14 Department of Thoracic Surgery, West China Hospital Sichuan University, Chengdu, China; 15 Department of Thoracic Surgery, Zhongshan Hospital, Fudan University, Shanghai, China; 16 Department of Thoracic Surgery, The Affiliated Hospital of Southwest Medical University, Luzhou, China; 17 Department of Thoracic Surgery, Union Hospital, Tongji Medical College, Huazhong University of Science and Technology, Wuhan, China; 18 Surgery of Esophageal Cancer, The First Affiliated Hospital of Henan University of Science and Technology, Luoyang, China; 19 Department of Thoracic Surgery, The Second Hospital & Clinical Medical School, Lanzhou University, Lanzhou, China; 20 Department of Thoracic Surgery, Cancer Hospital of University of Chinese Academy of Sciences, Zhejiang Cancer Hospital, Hangzhou, China; 21 Department of Thoracic Surgery, Shanxi Provincial Cancer Hospital, Taiyuan, China; 22 Department of Thoracic Surgery, First Affiliated Hospital of Zhengzhou University, Zhengzhou, China; 23 Department of Thoracic Surgery, Henan Provincial People’s Hospital, Zhengzhou, China; 24 Department of Esophageal Surgery, Shanghai Chest Hospital, Shanghai Jiao Tong University School of Medicine, Shanghai, China; 25 Department of Thoracic Surgery, The Fourth Hospital of Hebei Medical University, Shijiazhuang, China; 26 Department of Medical Affairs, Jiangsu Hengrui Pharmaceuticals Co, Ltd, Shanghai, China

**Keywords:** Randomized controlled trials, Oesophageal cancer, Cancer immunotherapy, Chemotherapy

## Abstract

Recent single-arm studies involving neoadjuvant camrelizumab, a PD-1 inhibitor, plus chemotherapy for resectable locally advanced esophageal squamous cell carcinoma (LA-ESCC) have shown promising results. This multicenter, randomized, open-label phase 3 trial aimed to further assess the efficacy and safety of neoadjuvant camrelizumab plus chemotherapy followed by adjuvant camrelizumab, compared to neoadjuvant chemotherapy alone. A total of 391 patients with resectable thoracic LA-ESCC (T1b-3N1-3M0 or T3N0M0) were stratified by clinical stage (I/II, III or IVA) and randomized in a 1:1:1 ratio to undergo two cycles of neoadjuvant therapy. Treatments included camrelizumab, albumin-bound paclitaxel and cisplatin (Cam+nab-TP group; *n* = 132); camrelizumab, paclitaxel and cisplatin (Cam+TP group; *n* = 130); and paclitaxel with cisplatin (TP group; *n* = 129), followed by surgical resection. Both the Cam+nab-TP and Cam+TP groups also received adjuvant camrelizumab. The dual primary endpoints were the rate of pathological complete response (pCR), as evaluated by a blind independent review committee, and event-free survival (EFS), as assessed by investigators. This study reports the final analysis of pCR rates. In the intention-to-treat population, the Cam+nab-TP and Cam+TP groups exhibited significantly higher pCR rates of 28.0% and 15.4%, respectively, compared to 4.7% in the TP group (Cam+nab-TP versus TP: difference 23.5%, 95% confidence interval (CI) 15.1–32.0, *P* < 0.0001; Cam+TP versus TP: difference 10.9%, 95% CI 3.7–18.1, *P* = 0.0034). The study met its primary endpoint of pCR; however, EFS is not yet mature. The incidence of grade ≥3 treatment-related adverse events during neoadjuvant treatment was 34.1% for the Cam+nab-TP group, 29.2% for the Cam+TP group and 28.8% for the TP group; the postoperative complication rates were 34.2%, 38.8% and 32.0%, respectively. Neoadjuvant camrelizumab plus chemotherapy demonstrated superior pCR rates compared to chemotherapy alone for LA-ESCC, with a tolerable safety profile. Chinese Clinical Trial Registry identifier: ChiCTR2000040034.

## Abstract (web summary)

In a randomized phase 3 trial, neoadjuvant anti-PD-1 plus either paclitaxel and cisplatin or nab-paclitaxel and cisplatin elicited a significantly superior pathological complete response rate versus neoadjuvant paclitaxel and cisplatin alone in patients with resectable locally advanced esophageal squamous cell carcinoma.

## Main

Esophageal cancer is a significant global health issue, ranking seventh in incidence and sixth in mortality among all cancers^1^, with over half of the global esophageal squamous cell carcinoma (ESCC) cases in China^2^. In East Asia, neoadjuvant chemotherapy or chemoradiotherapy is standard for resectable locally advanced ESCC (LA-ESCC), with chemotherapy more prevalent^3,4^. Studies such as CROSS and NEOCRTEC5010 highlight neoadjuvant chemoradiotherapy’s survival benefits over surgery alone^5,6^, whereas the JCOG9907 trial shows neoadjuvant chemotherapy improves overall survival (OS) compared to adjuvant therapy^7^. Recent phase 3 trials, including CMISG1701 and JCOG1109 (refs. ^8,9^), along with a network meta-analysis of randomized controlled trials^10^, have not demonstrated a significant OS advantage when comparing neoadjuvant chemoradiotherapy to chemotherapy for LA-ESCC, leaving the optimal neoadjuvant treatment strategy in question.

Immune checkpoint inhibitors (ICIs) have revolutionized the treatment of ESCC. Camrelizumab, a PD-1 inhibitor, has demonstrated promising efficacy and safety in advanced ESCC, including both chemotherapy-refractory and treatment-naive cases, as evidenced by the ESCORT and ESCORT-1st studies^11,12^. Following these results, China has approved camrelizumab as a second-line monotherapy for advanced or metastatic ESCC, and in combination with chemotherapy (paclitaxel and cisplatin, TP) as a first-line treatment. Several phase 1b and 2 trials assessing neoadjuvant immunotherapy with camrelizumab and chemotherapy for LA-ESCC report high pathological complete response (pCR) rates of 17.6% to 39.2% (refs. ^13–18^). Our latest retrospective analysis suggests that neoadjuvant chemotherapy plus immunotherapy showed better 3-year OS rates (91.7% versus 79.8%) and 3-year disease-free survival (DFS) rates (87.4% versus 72.8%) compared to neoadjuvant chemoradiotherapy^19^. Despite these promising results, there remains a lack of phase 3 confirmatory studies to further validate these findings.

Beyond selecting the optimal combination of treatment modalities, refining the chemotherapy regimen is crucial for enhancing neoadjuvant treatment outcomes in ESCC. The TP regimen is commonly used, yet nab-paclitaxel, an innovative albumin-bound formulation of paclitaxel, has shown a superior therapeutic profile compared to traditional paclitaxel. This preference for nab-paclitaxel, especially when combined with immunotherapy in LA-ESCC, is supported by several phase 2 studies^14–16^. Our retrospective analysis further substantiates this, revealing that neoadjuvant immunotherapy with nab-paclitaxel and cisplatin (nab-TP) achieves higher pCR rates than the combination of immunotherapy with TP regimen^20^. Against this backdrop, we initiated the ESCORT-NEO/NCCES01 study, a phase 3, open-label, randomized trial aimed at assessing the efficacy and safety of neoadjuvant camrelizumab plus either TP or nab-TP, as compared to TP alone, in patients with resectable LA-ESCC.

## Results

### Patient disposition

**Fig. 1:**
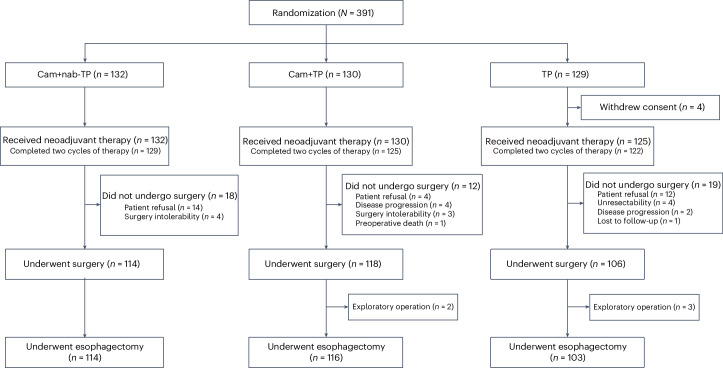
CONSORT diagram. A total of 411 patients were screened for this study, of whom 391 were successfully enrolled between April 28, 2021, and August 7, 2023.

A total of 411 patients were screened for this study, of whom 391 were successfully enrolled between April 28, 2021, and August 7, 2023. In the Cam+nab-TP, Cam+TP and TP groups, 132, 130 and 125 patients, respectively, were allocated and received neoadjuvant therapy; although the TP group initially had 129 before 4 withdrew consent. Consequently, the intention-to-treat (ITT) populations were 132, 130 and 129, with safety set (SS) populations of 132, 130 and 125, respectively (Fig. 1). As of the data cutoff on October 8, 2023, the median follow-up duration was 8.2 months (interquartile range (IQR), 3.5–15.6 months). The median age of all patients was 63 years (range, 44–75), with 84.9% being male. Among these patients, 6 (1.5%) were in clinical stage I (all cT1N1), 100 (25.6%) were stage II, 279 (71.4%) were stage III and 6 (1.5%) were stage IVA. Tumors were located in the upper, middle and lower thoracic esophagus for 41 (10.5%), 201 (51.4%) and 149 (38.1%) patients, respectively. Baseline characteristics were essentially balanced across all three groups (Table 1).

**Table 1:** Baseline characteristics of patients in the ITT population

Variables	Cam+nab-TP (*n* = 132)	Cam+TP (*n* = 130)	TP (*n* = 129)
Age (years)			
<65	74 (56.1)	79 (60.8)	63 (48.8)
≥65	58 (43.9)	51 (39.2)	66 (51.2)
Median (range)	63 (45–75)	63 (44–75)	65 (44–75)
Sex, *n* (%)			
Male	116 (87.9)	112 (86.2)	104 (80.6)
Female	16 (12.1)	18 (13.8)	25 (19.4)
ECOG PS, n (%)			
0	105 (79.5)	106 (81.5)	104 (80.6)
1	27 (20.5)	24 (18.5)	25 (19.4)
Tumor location, *n* (%)			
Upper	10 (7.6)	12 (9.2)	19 (14.7)
Middle	69 (52.3)	75 (57.7)	57 (44.2)
Lower	53 (40.2)	43 (33.1)	53 (41.1)
T stage, *n* (%)			
T1b	3 (2.3)	1 (0.8)	2 (1.6)
T2	15 (11.4)	13 (10.0)	19 (14.7)
T3	114 (86.4)	116 (89.2)	108 (83.7)
N stage, *n* (%)			
N0	20 (15.2)	24 (18.5)	20 (15.5)
N1	71 (53.8)	71 (54.6)	73 (56.6)
N2	38 (28.8)	33 (25.4)	35 (27.1)
N3	3 (2.3)	2 (1.5)	1 (0.8)
Clinical stage, *n* (%)			
I	3 (2.3)	1 (0.8)	2 (1.6)
II	31 (23.5)	34 (26.2)	35 (27.1)
III	95 (72.0)	93 (71.5)	91 (70.5)
IVA	3 (2.3)	2 (1.5)	1 (0.8)
PD-L1 TPS, *n* (%)			
<1%	43 (32.6)	59 (45.4)	49 (38.0)
≥1%	78 (59.1)	61 (46.9)	62 (48.1)
<10%	99 (75.0)	98 (75.4)	97 (75.2)
≥10%	22 (16.7)	22 (16.9)	14 (10.9)
Unknown	11 (8.3)	10 (7.7)	18 (14.0)
PD-L1 CPS, *n* (%)			
<1	14 (10.6)	18 (13.8)	15 (11.6)
≥1	109 (82.6)	102 (78.5)	96 (74.4)
<10	68 (51.5)	80 (61.5)	72 (55.8)
≥10	55 (41.7)	40 (30.8)	39 (30.2)
Unknown	9 (6.8)	10 (7.7)	18 (14.0)

ECOG PS, Eastern Cooperative Oncology Group performance status; TPS, tumor proportion score; CPS, combined positive score.

### Neoadjuvant treatment and surgery summary

Within the ITT population, 132, 130 and 125 patients in the Cam+nab-TP, Cam+TP and TP groups, respectively, received neoadjuvant therapy. Of these, 3 in the Cam+nab-TP group, 5 in the Cam+TP group and 3 in the TP group did not complete two cycles of neoadjuvant therapy for several reasons; in the Cam+nab-TP group, two patients discontinued due to adverse events (AEs) and one due to patient refusal; in the Cam+TP group, four patients discontinued due to AEs and one due to death; and in the TP group, discontinuations were due to one AE and two instances of patient refusal. A total of 114 (86.4%), 116 (89.2%) and 103 (79.8%) underwent esophagectomy. Reasons for cancellation of esophagectomy included refusal of surgery (*n* = 30), surgery intolerability (*n* = 7), disease progression (*n* = 6), exploratory operation (*n* = 5), unresectability (*n* = 4), preoperative death (*n* = 1) and loss to follow-up (*n* = 1). Among the exploratory operations, one case in the TP group revealed peritoneal metastasis, while the remaining four cases involved tumors deemed unresectable due to extensive invasion. The median time from the last neoadjuvant treatment to surgery across the Cam+nab-TP, Cam+TP, and TP groups were 5.9 (IQR, 5.0–7.1), 5.7 (IQR, 5.0–7.4) and 5.4 (IQR, 4.9–6.3) weeks, respectively. In terms of types of surgery, the McKeown procedure was the most common, accounting for 93.9% in the Cam+nab-TP group, 91.4% in the Cam+TP group and 92.2% in the TP group. The median number of lymph nodes harvested was 34 (IQR, 24–50), 37 (IQR, 27–48) and 32 (IQR, 27–45), respectively. The median duration of surgery for the Cam+nab-TP, Cam+TP and TP groups was 4.3 hours (range, 2.6–8.9), 4.2 hours (range, 2.8–7.2) and 4.2 hours (range, 2.9–10.8), respectively (Table 2).

**Table 2:** Surgery summary

Variables	Cam+nab-TP (*n* = 114)	Cam+TP (*n* = 116)	TP (*n* = 103)
Esophagectomy rate (%)^a^	86.4 (114/132)	89.2 (116/130)	79.8 (103/129)
Types of surgical procedures, n (%)			
McKeown	107 (93.9)	106 (91.4)	95 (92.2)
Ivor-Lewis	6 (5.3)	10 (8.6)	7 (6.8)
Sweet	1 (0.9)	0	0
Other	0	0	1 (1.0)
Margin status, *n* (%)			
R0	113 (99.1)	111 (95.7)	95 (92.2)
R1	1 (0.9)	4 (3.4)	6 (5.8)
R2	0	1 (0.9)	2 (1.9)
Number of lymph nodes harvested			
Median (IQR)	34 (24–50)	37 (27–48)	32 (27–45)
Time from last neoadjuvant treatment to surgery (weeks)			
Median (IQR)	5.9 (5.0–7.1)	5.7 (5.0–7.4)	5.4 (4.9–6.3)
Duration of surgery (hours)			
Median (range)	4.3 (2.6–8.9)	4.2 (2.8–7.2)	4.2 (2.9–10.8)
Reoperations, *n* (%)^b^	0	1 (0.9)	1 (1.0)
Mortality within 30 days, *n* (%)^c^	1 (0.9)	2 (1.7)	1 (1.0)
Mortality within 90 days, *n* (%)^d^	2 (1.8)	2 (1.7)	1 (1.0)

^a^Based on ITT population.

^b^Two patients underwent reoperation for adhesive intestinal obstruction (Cam+TP group) or anastomotic leak (TP group).

^c^Mortality within 30 days included sudden postoperative death, cause unknown (Cam+nab-TP group), septic shock (Cam+TP group) and myocardial infarction (TP group).

^d^Mortality within 90 days included mortality within 30 days, with one more death in Cam+nab-TP group (severe pneumonia).

### Primary outcome

**Extended Data Fig. 1:**
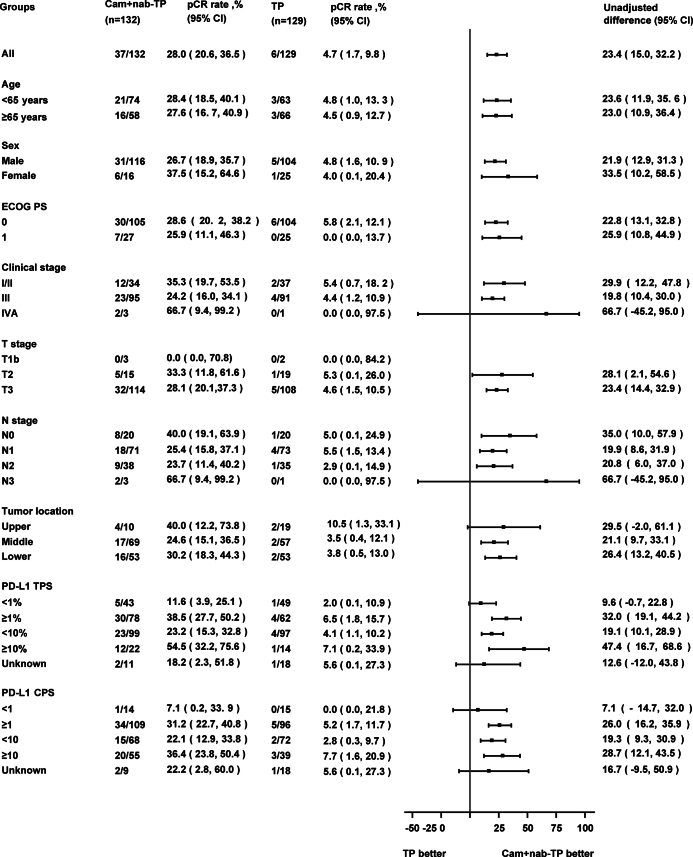
Post hoc subgroup analysis of pCR between Cam+nab-TP group and TP group. The central points on each bar represent the mean difference in pCR rates between the two groups, while the bars indicate the 95% CI. pCR, pathological complete response; ECOG PS, Eastern Cooperative Oncology Group performance status; TPS, tumor proportion score; CPS, combined positive score; CI, confidence interval.

**Extended Data Fig. 2:**
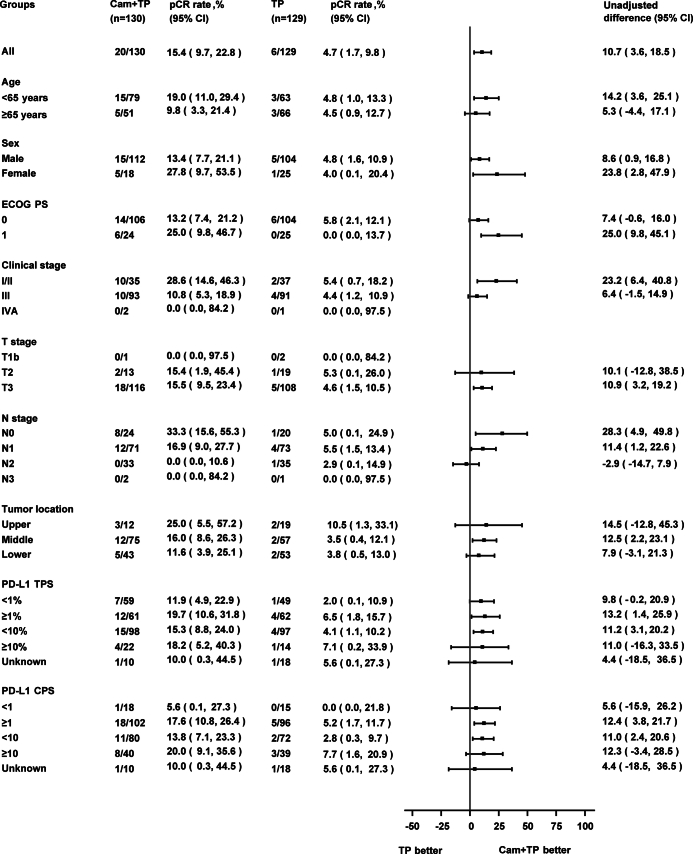
Post hoc subgroup analysis of pCR between Cam+TP group and TP group. The central points on each bar represent the mean difference in pCR rates between the two groups, while the bars indicate the 95% CI. pCR, pathological complete response; ECOG PS, Eastern Cooperative Oncology Group performance status; TPS, tumor proportion score; CPS, combined positive score; CI, confidence interval.

Within the ITT population, the pCR rate was 28.0% in the Cam+nab-TP group, markedly higher than the TP group’s 4.7% (difference 23.5%, 95% confidence interval (CI), 15.1–32.0; odds ratio (OR), 8.11; 95% CI, 3.28–20.06; P < 0.0001). The Cam+TP group’s pCR rate was also significantly greater at 15.4%, compared to the TP group (difference 10.9%, 95% CI, 3.7–18.1; OR, 3.81; 95% CI, 1.48–9.80; P = 0.0034) (Table 3). Post hoc subgroup analyses of pCR rates for the Cam+nab-TP group versus TP group and Cam+TP group versus TP group are presented in Extended Data Figs. 1 and 2. Event-free survival (EFS) data have not matured.

**Table 3:** Pathological outcomes according to blinded independent pathological review in the ITT population

Variables	Cam+nab-TP (*n* = 132)	Cam+TP (*n* = 130)	TP (*n* = 129)
pCR			
Rate, % (95%CI)^a^	28.0 (20.6, 36.5)	15.4 (9.7, 22.8)	4.7 (1.7, 9.8)
Difference (vs TP group), % (95%CI)^b^	23.5 (15.1, 32.0)	10.9 (3.7, 18.1)	
OR (vs TP group) (95%CI)^b^	8.11 (3.28, 20.06)	3.81 (1.48, 9.80)	
*P* value (vs TP group)^c^	<0.0001	0.0034	
MPR			
Rate, % (95%CI)^a^	59.1 (50.2, 67.6)	36.2 (27.9, 45.0)	20.9 (14.3, 29.0)
Difference (vs TP group), % (95%CI)^b^	38.3 (27.4, 49.3)	15.4 (4.7, 26.2)	
OR (vs. TP group) (95%CI)^b^	5.51 (3.18, 9.56)	2.19 (1.25, 3.84)	

^a^95%CI were calculated based on the Clopper-Pearson method.

^b^95%CI for the stratification factor-adjusted rate differences were derived using the Mantel-Haenszel method.

^c^The Cochran-Mantel-Haenszel test, stratified by clinical stage (I/II versus III/IVA), was used to compare between groups.

MPR, major pathological response.

### Secondary outcomes

**Extended Data Fig. 3:**
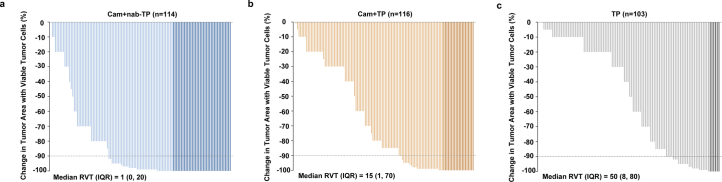
Depth of pathological regression in primary tumor. (**a**) Cam+nab-TP group, (**b**) Cam+TP group and (**c**) TP group. RVT, residual viable tumor cells; IQR, interquartile range.

Within the ITT population, the major pathological response (MPR) rates in the Cam+nab-TP, Cam+TP, and TP groups were 59.1%, 36.2% and 20.9%, respectively (Table 3). The R0 resection rates were 99.1% (113/114), 95.7% (111/116) and 92.2% (95/103) for the Cam+nab-TP, Cam+TP and TP groups, respectively (Table 2). For the post-neoadjuvant pathological staging (ypTNM) staging, 58 patients (50.9%) achieved stage I in the Cam+nab-TP group, 46 (39.7%) in the Cam+TP group and 27 (26.2%) in the TP group (Supplementary Table 1). The median residual viable tumor cells were 1% (IQR, 0–20), 15% (IQR, 1–70) and 50% (IQR, 8–80) for Cam+nab-TP, Cam+TP and TP groups, respectively (Extended Data Fig. 3). DFS and OS are not yet mature.

### Safety

In the Cam+nab-TP, Cam+TP and TP groups, the rates of surgical complications of any grade were 34.2% (39/114), 38.8% (45/116) and 32.0% (33/103), respectively. Among these, the proportions of Clavien-Dindo (CD) grade 3 or higher complications were 6.1% (7/114), 12.1% (14/116) and 6.8% (7/103), respectively. Pneumonia and recurrent laryngeal nerve injury were the most common postoperative complications (Table 4 and Supplementary Table 2). One patient (0.9%) in the Cam+TP group and one patient (1.0%) in the TP group required reoperation due to adhesive intestinal obstruction and an anastomotic leak, respectively. The 30-day postoperative mortality rates were 0.9% in the Cam+nab-TP group (one case of sudden death, cause unknown), 1.7% in the Cam+TP group (two cases of septic shock) and 1.0% in the TP group (one case of myocardial infarction). There was one additional death within 90 days postoperatively in the Cam+nab-TP group, due to severe pneumonia (Table 2).

**Table 4:** Surgical complications according to CD classification in at least two patients in all groups

Events, *n* (%)	Cam+nab-TP (*n* = 114)		Cam+TP (*n* = 116)		TP (*n* = 103)	
	Any grade	Grade ≥3	Any grade	Grade ≥3	Any grade	Grade ≥3
Any events	39 (34.2)	7 (6.1)	45 (38.8)	14 (12.1)	33 (32.0)	7 (6.8)
Pneumonia	12 (10.5)	0	21 (18.1)	1 (0.9)	15 (14.6)	2 (1.9)
Recurrent laryngeal nerve injury	11 (9.6)	0	11 (9.5)	1 (0.9)	9 (8.7)	1 (1.0)
Dysrhythmia	7 (6.1)	0	2 (1.7)	0	3 (2.9)	0
Pleural effusion	3 (2.6)	3 (2.6)	12 (10.3)	7 (6.0)	7 (6.8)	3 (2.9)
Anastomotic leak	3 (2.6)	1 (0.9)	5 (4.3)	2 (1.7)	6 (5.8)	1 (1.0)
Conduit necrosis	2 (1.8)	0	1 (0.9)	0	1 (1.0)	0
Respiratory failure	1 (0.9)	1 (0.9)	0	0	1 (1.0)	1 (1.0)
Intrathoracic abscess	1 (0.9)	1 (0.9)	0	0	1 (1.0)	0
Delirium	1 (0.9)	0	0	0	1 (1.0)	0
Septic shock	0	0	3 (2.6)	3 (2.6)	0	0
Atelectasis	0	0	1 (0.9)	0	1 (1.0)	1 (1.0)
Chylous leak	0	0	0	0	2 (1.9)	0
Delayed conduit emptying	0	0	0	0	2 (1.9)	1 (1.0)

Regarding treatment-related AEs (TRAEs), the incidence rates were 93.9% (124/132) in the Cam+nab-TP group, 83.1% (108/130) in the Cam+TP group and 83.2% (104/125) in the TP group. Grade 3 or higher TRAEs occurred in 34.1% (45/132) of the Cam+nab-TP group, 29.2% (38/130) of the Cam+TP group and 28.8% (36/125) of the TP group (Supplementary Table 3). The most prevalent grade 3 or higher TRAEs were neutrophil count decreased and white blood cell count decreased (Table 5 and Supplementary Table 4). The rates of TRAEs leading to chemotherapy discontinuation were 3.0% for Cam+nab-TP, 3.8% for Cam+TP and 0.8% for the TP group. The rates of discontinuing camrelizumab due to TRAEs were 0.8% for both the Cam+nab-TP and Cam+TP groups. Immune-related AEs (irAEs) were only reported in treatment groups that included camrelizumab, with incidences of 27.3% (36/132) in the Cam+nab-TP group and 24.6% (32/130) in the Cam+TP group. Grade 3 or higher irAEs were observed in 4.5% (6/132) of the Cam+nab-TP group and 3.8% (5/130) of the Cam+TP group. The most common irAE was reactive cutaneous capillary endothelial proliferation, which was grade 1–2 in all cases (Supplementary Table 5).

**Table 5:** Preoperative TRAE in at least 5% of patients in any group

Event, *n* (%)	Cam+nab-TP (*n* = 132)		Cam+TP (*n* = 130)		TP (*n* = 125)	
	Any grade	Grade ≥3	Any grade	Grade ≥3	Any grade	Grade ≥3
Any TRAE	124 (93.9)	45 (34.1)	108 (83.1)	38 (29.2)	104 (83.2)	36 (28.8)
White blood cell count decreased	68 (51.5)	15 (11.4)	51 (39.2)	13 (10.0)	41 (32.8)	6 (4.8)
Neutrophil count decreased	61 (46.2)	33 (25.0)	48 (36.9)	26 (20.0)	38 (30.4)	29 (23.2)
Anemia	41 (31.1)	0	30 (23.1)	2 (1.5)	23 (18.4)	1 (0.8)
Nausea	40 (30.3)	2 (1.5)	28 (21.5)	0	33 (26.4)	1 (0.8)
Alopecia	36 (27.3)	0	30 (23.1)	0	25 (20.0)	0
Lymphocyte count decreased	21 (15.9)	7 (5.3)	12 (9.2)	3 (2.3)	10 (8.0)	1 (0.8)
Vomiting	20 (15.2)	1 (0.8)	9 (6.9)	0	18 (14.4)	2 (1.6)
Platelet count decreased	15 (11.4)	0	8 (6.2)	2 (1.5)	5 (4.0)	0
Hypokalemia	15 (11.4)	2 (1.5)	3 (2.3)	0	7 (5.6)	1 (0.8)
Hyponatremia	14 (10.6)	2 (1.5)	6 (4.6)	2 (1.5)	4 (3.2)	2 (1.6)
Fatigue	13 (9.8)	0	16 (12.3)	0	11 (8.8)	0
Creatinine increased	13 (9.8)	1 (0.8)	10 (7.7)	0	11 (8.8)	0
Rash	12 (9.1)	0	14 (10.8)	1 (0.8)	8 (6.4)	0
Anorexia	12 (9.1)	0	13 (10.0)	0	7 (5.6)	0
Reactive cutaneous capillary endothelial proliferation	11 (8.3)	0	13 (10.0)	0	0	0
Alanine aminotransferase increased	10 (7.6)	1 (0.8)	11 (8.5)	2 (1.5)	6 (4.8)	0
Diarrhea	8 (6.1)	1 (0.8)	9 (6.9)	1 (0.8)	6 (4.8)	0
Aspartate aminotransferase increased	6 (4.5)	0	9 (6.9)	2 (1.5)	3 (2.4)	0
Myalgia	4 (3.0)	0	6 (4.6)	0	9 (7.2)	0
Arthralgia	4 (3.0)	0	9 (6.9)	0	5 (4.0)	0
Dysesthesia	3 (2.3)	0	4 (3.1)	0	7 (5.6)	0

## Discussion

To the best of our knowledge, the ESCORT-NEO/NCCES01 study represents the first phase 3 trial to assess the efficacy and safety of neoadjuvant immunotherapy combined with chemotherapy versus chemotherapy in LA-ESCC. Our findings demonstrate that the addition of camrelizumab to chemotherapy substantially enhances pCR rates in the ITT population. Specifically, the pCR rate for the Cam+nab-TP group and the TP group was 28.0% versus 4.7% (difference: 23.5%, 95% CI, 15.1–32.0; OR: 8.11, 95% CI, 3.28–20.06); for Cam+TP and TP: 15.4% versus 4.7% (difference: 10.9%, 95% CI, 3.7–18.1; OR: 3.81, 95% CI, 1.48–9.80). Our pCR outcomes exceed those of traditional neoadjuvant chemotherapy, which was 9% (95% CI, 6%-14%), and are numerically comparable to neoadjuvant chemoradiotherapy’s 32% (95% CI, 26%-39%) for LA-ESCC in a meta-analysis^21^. These results underscore the substantial potential of combining neoadjuvant immunotherapy with chemotherapy in the treatment of LA-ESCC.

Achieving a pCR in patients with locally advanced esophageal cancer after neoadjuvant chemoradiotherapy is associated with improved OS, yet distant recurrence remains prevalent^22^, highlighting the necessity for more effective systemic treatment, rather than locoregional treatment, to enhance survival outcomes. The impact of pCR varies across different neoadjuvant regimens. An international cohort study comparing neoadjuvant chemotherapy with chemoradiotherapy in surgically treated esophageal adenocarcinoma revealed a notable decrease in 5-year relapse-free survival for patients achieving pCR with chemoradiotherapy compared to chemotherapy (75.3% vs 87.1%), with chemoradiotherapy associated with a higher incidence of 5-year distant recurrence (OR, 2.50; 95% CI 1.25–4.99)^23^. Furthermore, the JCOG1109 trial indicated that neoadjuvant CF (cisplatin and fluorouracil) plus radiotherapy did not significantly enhance survival compared to CF alone (hazard ratio (HR), 0.84; 95% CI, 0.63–1.12) despite higher pCR rate, whereas neoadjuvant DCF (docetaxel, cisplatin and fluorouracil) demonstrated both higher pCR rate (18.6% versus 2.2%), and long-term survival benefits (HR, 0.68; 95% CI, 0.50–0.92)^9^. Neoadjuvant immunotherapy aims to boost systemic immune responses to tumor antigens, potentially eradicating micro metastatic tumor deposits that could lead to relapse after surgery^24^. In this study, neoadjuvant immunotherapy plus chemotherapy showed higher pCR rates, which may offer long-term survival benefits. However, the confirmation of these advantages awaits the maturation of our long-term survival data.

Recently, several studies have investigated the role of neoadjuvant immunotherapy in upper gastrointestinal malignancies. The KEYNOTE-585 trial represents a phase 3 study investigating neoadjuvant pembrolizumab combined with chemotherapy in gastric and gastroesophageal junction cancers. Although it did not meet its predefined endpoint for EFS, the treatment group exhibited a median EFS of 44.4 months, compared to 25.3 months in the control group, with a HR of 0.81 (95% CI, 0.67–0.99)^25^. The subgroup analysis suggests a trend of benefit, particularly in patients with higher combined positive scores (CPS), echoing results from other phase 3 studies like KEYNOTE-859 (ref. ^26^) and Checkmate-649 (ref. ^27^), which highlighted significant survival benefits in advanced gastric cancer treated with immunotherapy combined with chemotherapy. Similarly, the MATTERHORN^28^ and DANTE/FLOT8 trials^29^, which focused on gastric adenocarcinoma and gastroesophageal junction adenocarcinoma, demonstrated promising pCR rates, further supporting the efficacy of neoadjuvant immunotherapy regimens. However, these trials largely involved gastric cancer patients, which may show different prognoses from those with esophageal cancer, likely due to the potent chemotherapy regimens and tumor heterogeneity.

In the realm of esophageal cancer, a systematic review and meta-analysis that included 27 phase 2 trials encompassing 815 patients underscores the promising clinical and safety outcomes of neoadjuvant immunotherapy combined with chemotherapy in patients with resectable esophageal cancer^30^. Moreover, the ongoing phase 2/3 ECOG-ACRIN EA2174 trial^31^, which explores a combination of neoadjuvant carboplatin and paclitaxel with concurrent radiation, either with or without nivolumab, followed by adjuvant nivolumab with or without ipilimumab, is poised to further our understanding of perioperative immunotherapy in locoregional esophageal and gastroesophageal junction adenocarcinoma. Distinguishing between ESCC and adenocarcinoma is crucial, as these subtypes vary significantly in pathogenesis, epidemiology, and prognosis^32^. Notably, the phase 3 ESCORT-NEO/NCCES01 study fills a crucial gap in our understanding and provides critical evidence for the efficacy of neoadjuvant immunotherapy plus chemotherapy in ESCC, demonstrating more significant improvements in pCR rates than neoadjuvant chemotherapy alone. In addition to ESCORT-NEO/NCCES01, several other ongoing phase 3 studies are exploring the role of neoadjuvant immunotherapy in ESCC. These include NCT04848753, which compares neoadjuvant immunochemotherapy to chemotherapy alone; and the KEYSTONE-002 study^33^, which assesses neoadjuvant immunochemotherapy versus chemoradiotherapy. We anticipate the outcomes from these studies will further elucidate the role of neoadjuvant immunotherapy in the management of ESCC.

In our study, the pCR rate in the Cam+nab-TP group was numerically higher than in the Cam+TP group, which might be attributable to prophylactic corticosteroid for paclitaxel before neoadjuvant therapy^34^. Corticosteroid use before ICI therapy could influence efficacy, potentially by inhibiting the proliferation of CD8-positive T cells required for an ICI response^35^. Additionally, the administered frequency and total dose of nab-paclitaxel seemed higher than that of paclitaxel in this study; nab-paclitaxel was administered at 125 mg/m^2^ on days 1 and 8 of each cycle, while paclitaxel was given at 175 mg/m^2^ only on day 1 of each cycle. A more intensive chemotherapy regimen may further contribute to improved efficacy. However, beyond the administered frequency and total dose, it is important to note that differential clinical activity between nab-paclitaxel and paclitaxel has been observed, possibly due to better drug delivery and reduced toxicities^36^. Our retrospective study observed that the pCR rate for immunotherapy combined with nab-paclitaxel and platinum-based chemotherapy was 36.7%, exceeding the 21.4% when combined with paclitaxel and platinum-based chemotherapy for LA-ESCC^20^. This observation aligns with studies in other cancers, such as lung squamous carcinoma and triple-negative breast cancer, where immunotherapy with nab-paclitaxel demonstrated survival benefits^37,38^. Furthermore, meta-analyses showed that nab-paclitaxel can improve pCR rates and survival outcomes when compared with solvent-based paclitaxel^39,40^, suggesting that the observed differences in pCR rates may also stem from the inherent properties of the two drugs. Although our study highlights potential benefits of combining ICIs with nab-paclitaxel in LA-ESCC, the lack of a prespecified hypothesis for comparing the Cam+nab-TP and Cam+TP groups necessitates cautious interpretation of these results. Future research should include direct comparative phase 3 randomized controlled trials to elucidate the efficacy and safety of ICI in combination with different taxane-based chemotherapies in treating ESCC.

PD-L1 expression, a key biomarker for immunotherapy in ESCC, plays a significant role in guiding treatment decisions. High PD-L1 levels (CPS ≥ 10) are linked to improved survival with ICI monotherapy^41,42^. The ESCORT study revealed that camrelizumab offers clinical benefits across all PD-L1 levels, with greater advantages for those with higher expression^11^. Nevertheless, PD-L1’s role as a biomarker is less clear when immunotherapy is combined with chemotherapy, as studies suggest even patients with low PD-L1 expression can benefit from this treatment^43^. For instance, the ESCORT-1st study showed enhanced OS for patients with PD-L1 expression ≥1%, though without statistical significance^12^. In our study, approximately half of the patients (51.4%) exhibited PD-L1 positivity (tumor proportion score [TPS] ≥1%), aligning with the findings from both the ESCORT (42.6%) and ESCORT-1st studies (55.2%). Our analysis also highlighted a pronounced pCR advantage in patients with higher PD-L1 expression receiving combined treatment. Ultimately, while PD-L1 expression aids in assessing the potential of neoadjuvant immunotherapy with chemotherapy in ESCC, its accuracy requires further validation.

In the three groups, resection rates were 86.4%, 89.2% and 79.8%, respectively, indicating that neoadjuvant therapy did not notably affect the execution of surgery. Notably, the median duration of surgery was comparable across all groups, suggesting that adding neoadjuvant immunotherapy to chemotherapy may not complicate surgical procedures. The risks of postoperative morbidity and mortality were similar among the groups, indicating manageable safety for surgery after combined neoadjuvant therapy. These extended dissections resulted in a median of over 30 lymph nodes harvested, surpassing the minimum count necessary for precise staging and management.

Toxicity associated with neoadjuvant camrelizumab combined with chemotherapy aligned with previous studies, without new safety concerns. The most common severe TRAEs included neutrophil count decreased and white blood cell count decreased, which are recognized AEs of camrelizumab with chemotherapy^12–17^. The Cam+nab-TP group exhibited numerically higher rates of total TRAEs at 93.9% and grade ≥3 TRAEs at 34.1%, compared to 83.1% and 29.2% in the Cam+TP group, and 83.2% and 28.8% in the TP group. The primary increased TRAEs in the Cam+nab-TP group were manageable and included conditions such as white blood cell count decreased, neutrophil count decreased, anemia, lymphocyte count decreased, platelet count decreased and vomiting. These TRAEs were effectively managed with established clinical protocols, and there were no new or uncontrollable safety signals observed. Additionally, the rates of TRAEs leading to chemotherapy discontinuation were low and comparable across all groups, at 3.0% for Cam+nab-TP, 3.8% for Cam+TP, and 0.8% for the TP group. Similarly, the rates of discontinuation of camrelizumab due to TRAEs were also low, at 0.8% for both the Cam+nab-TP and Cam+TP groups. Therefore, despite the numerically higher incidence of TRAEs in the Cam+nab-TP group, these were not associated with an increased risk of serious adverse effects, suggesting that the increased TRAEs may not represent a significant safety concern. Notably, the incidence of grade 3 or higher TRAEs in our study’s camrelizumab with chemotherapy group was numerically lower than that reported in the ESCORT-1st study (63.4%)^12^, potentially attributable to fewer treatment cycles. This observation raises the question of whether the occurrence of AEs might accumulate with an increasing number of treatment cycles, meriting further exploration. The most frequent irAE was reactive cutaneous capillary endothelial proliferation, all instances of which were grade 1 or 2. A grade 5 TRAE occurred in the Cam+TP group, where a patient experienced acute liver failure on the first day of neoadjuvant therapy, potentially linked to camrelizumab, paclitaxel or cisplatin. This case highlights the necessity of vigilant patient monitoring during combined immunotherapy.

This study has several limitations. First, this study did not directly compare the two neoadjuvant chemotherapy regimens combined with camrelizumab that were tested. Furthermore, although neoadjuvant chemoradiotherapy is a standard treatment for LA-ESCC, our study did not directly compare neoadjuvant immunochemotherapy with chemoradiotherapy. Previous studies have indicated that patients with ESCC in East Asia respond less to neoadjuvant chemoradiotherapy compared to their Caucasian counterparts^44^. Moreover, neoadjuvant chemotherapy is currently more widely accepted in East Asia. Second, another dual primary endpoint of this study, EFS, has not yet matured. Although prior studies have indicated that pathological response is correlated with long-term survival^45^, suggesting it could act as a surrogate endpoint for neoadjuvant immunotherapy, survival outcomes remain the gold standard in phase 3 studies. Another limitation of this study is that it was conducted exclusively in China, which may restrict the generalizability of the findings to populations in other regions. Moreover, the non-blinded design of this study could introduce some degree of bias. Besides, the current study is limited by the absence of comprehensive biomarker data, including a relatively high number of patients with unknown PD-L1 status. It should be noted that biomarker and patient-reported outcomes data are currently being collected as exploratory endpoints to enhance the understanding of treatment effects.

In conclusion, this study demonstrated that camrelizumab combined with chemotherapy significantly increased the pCR in the ITT population. The combined treatment regimen was found to be safe, with no unexpected increase in toxicity. This study contributes new evidence supporting neoadjuvant treatment in LA-ESCC and lays the foundation for developing and optimizing future treatment strategies. We anticipate further confirmation of our findings as survival data continue to mature.

## Methods

### Study design and patients

The ESCORT-NEO/NCCES01 study is a multicenter, randomized, open-label, phase 3 trial conducted across 24 centers in China, which enrolled patients with LA-ESCC. The inclusion criteria were: 1) provision of a written informed consent form and voluntary participation in the study; 2) histopathological or cytological confirmation of ESCC; 3) Thoracic esophageal cancer confirmed by computed tomography (CT), magnetic resonance imaging (MRI), endoscopic ultrasound (EUS), clinically staged as T1b-3N1-3M0 or T3N0M0 according to the 8th edition of the American Joint Committee on Cancer staging system; 4) expectation to achieve a R0 resection; 5) age between 18 and 75 years, applicable to both male and female patients; 6) Eastern Cooperative Oncology Group performance status (ECOG PS) of 0–1; 7) no prior anti-tumor therapy for esophageal cancer, including radiotherapy, chemotherapy or surgery; 8) scheduled for surgery following the completion of neoadjuvant therapy; 9) no contraindications to undergoing surgery; 10) normal function of major organs, including a) hematology (no administration of blood components, cell growth factors, leukocyte-elevating drugs, platelet-elevating drugs, or anemia-correcting drugs within 14 days before the first administration of the study drug) (neutrophil count ≥1.5 × 10^9^ liter^−1^, platelet count ≥100 × 10^9^ liter^−1^, hemoglobin ≥90 g liter^−1^); b) blood biochemistry (total bilirubin ≤1.5× upper limit of normal (ULN), alanine aminotransferase ≤2.5× ULN, aspartate aminotransferase ≤2.5× ULN, serum creatinine ≤1.5× ULN or creatinine clearance ≥50 ml min^−1^ (using the Cockcroft-Gault formula); c) coagulation function: international normalized ratio ≤1.5× ULN, activated partial thromboplastin time ≤ 1.5×ULN; 11) female participants of childbearing potential must have a negative serum pregnancy test result within 72 h before the first dose of the study drug and agree to use effective contraception (for example, intrauterine devices, hormonal contraceptives or condoms) during the study period and for at least 3 months following the last dose, and male participants with partners of childbearing potential must be surgically sterile or agree to use effective contraception during the study and for 3 months after the last dose; and 12) participants must demonstrate good compliance and cooperation with follow-up requirements throughout the study.

Patients with any of the following conditions were excluded from participating in this study: 1) Presence of a tumor with obvious invasion into adjacent organs of the esophageal lesion, such as major arteries or the trachea. 2) Metastases to supraclavicular lymph nodes. 3) Uncontrollable pleural effusion, pericardial effusion or ascites requiring repeated drainage. 4) Poor nutritional status, defined as a body mass index less than 18.5 kg/m^2^; however, patients whose nutritional status improved after symptomatic support and who were subsequently reassessed and approved by the principal investigator were considered for enrollment. 5) History of allergy to monoclonal antibodies, any component of camrelizumab, paclitaxel, cisplatin or other platinum-based drugs. 6) Prior or ongoing treatment that included: a) any form of radiotherapy, chemotherapy or other anti-tumor drugs; b) use of immunosuppressive drugs or systemic corticosteroid therapy for immunosuppressive purposes (doses exceeding 10 mg day^−1^ prednisone or its equivalent) within 2 weeks before the first dose of the study drug (inhaled or topical steroids, as well as adrenal corticosteroid replacement therapy exceeding doses of 10 mg day^−1^ prednisone or equivalent, were allowed, provided there was no active autoimmune disease); c) receipt of a live attenuated vaccine within 4 weeks before the first dose of the study drug; or d) undergoing major surgery or experiencing serious trauma within 4 weeks before the first dose of the study drug. 7) History of any active or past autoimmune disease, including, but not limited to, interstitial pneumonia, enteritis, hepatitis, hypophysitis, vasculitis, nephritis, hyperthyroidism and hypothyroidism (even if under hormone replacement therapy). Exceptions may be made for patients with historical psoriasis or childhood asthma/allergy that resolved without intervention in adulthood; however, patients requiring ongoing medical intervention with bronchodilators were excluded. 8) History of immunodeficiency, including those testing positive for human immunodeficiency virus, other acquired or congenital immune deficiencies, or a history of organ transplantation or allogeneic bone marrow transplantation. 9) Presence of clinically uncontrolled cardiac symptoms or diseases, including but not limited to: New York Heart Association class II or above heart failure, unstable angina pectoris, myocardial infarction within the past year or clinically notable supraventricular or ventricular arrhythmias that were either untreated or uncontrolled with treatment. 10) Any serious infection (National Cancer Institute Common Terminology Criteria for Adverse Events > grade 2) within 4 weeks before the first dose of the study drug, including severe pneumonia, bacteremia or infection complications requiring hospitalization; active pulmonary inflammation evident on baseline chest X-ray; or symptoms and signs of infection requiring oral or intravenous antibiotic therapy within 14 days before the first dose of the study drug, excluding prophylactic use of antibiotics. 11) Patients with an active tuberculosis infection based on medical history or CT test, those who have had an active tuberculosis infection within the past year, or those with a history of tuberculosis beyond one year without proper treatment. 12) Presence of active hepatitis B virus, defined as hepatitis B virus DNA levels ≥2,000 IU ml^−1^ or 10^4^ copies ml^−1^ or active hepatitis C virus, indicated by positive hepatitis C antibody and hepatitis C virus RNA levels above the lower limit of detection of the assay. 13) Any other malignancies diagnosed within the past 5 years before the first dose of the study drug, with the exception of malignancies with a low risk of metastasis or death (5-year survival rate >90%), such as adequately treated basal cell carcinoma of the skin, squamous cell skin cancer or carcinoma in situ of the cervix. 14) Pregnant or lactating women. 15) Presence of other factors that, in the investigator’s judgment, may necessitate forced withdrawal from the study. These factors included other serious diseases (including psychiatric disorders) that required concomitant therapy, alcoholism, drug abuse or family or social factors that may compromise the safety or compliance of the subjects.

The study protocol was approved by the Ethics Committee of the National Cancer Center/National Clinical Research Center for Cancer/Cancer Hospital, Chinese Academy of Medical Sciences and Peking Union Medical College; Anyang Cancer Hospital; Tangdu Hospital, Air Force Military Medical University; Fujian Provincial Cancer Hospital; Tianjin Medical University Cancer Institute and Hospital; Fujian Medical University Union Hospital; Ruijin Hospital, Shanghai Jiao Tong University School of Medicine; Qilu Hospital of Shandong University; The First Affiliated Hospital of Xi’an Jiaotong University; Harbin Medical University Cancer Hospital; Affiliated Hospital of North Sichuan Medical College; Sichuan Cancer Hospital; West China Hospital, Sichuan University; Zhongshan Hospital, Fudan University; The Affiliated Hospital of Southwest Medical University; Union Hospital, Tongji Medical College, Huazhong University of Science and Technology; The First Affiliated Hospital of Henan University of Science and Technology; The Second Hospital & Clinical Medical School, Lanzhou University; Cancer Hospital of University of Chinese Academy of Sciences, Zhejiang Cancer Hospital; Shanxi Provincial Cancer Hospital; First Affiliated Hospital of Zhengzhou University; Henan Provincial People’s Hospital; Shanghai Chest Hospital, Shanghai Jiao Tong University School of Medicine; The Fourth Hospital of Hebei Medical University. All enrolled patients provided written informed consent. The study was registered before patient enrollment (ChiCTR2000040034). An independent data monitoring committee was established to ensure ongoing participant safety and data integrity throughout the trial.

The sex of participants was collected according to the identity information provided by the patients. Gender, as shaped by social and cultural circumstances, was not specifically assessed or reported in the study design. Both male and female patients were eligible. Post hoc subgroup analysis of pCR was performed based on sex (male versus female). Patients were provided with the study treatment for free (including camrelizumab, paclitaxel, albumin-bound paclitaxel and cisplatin) and also received a transportation reimbursement.

### Procedure

Eligible patients were randomly assigned in a 1:1:1 ratio to either the Cam+nab-TP group, the Cam+TP group, or the TP group using the randomized trial management system. Randomization was stratified according to clinical stage into I/II, III, and IVA. All patients underwent two 3-week cycles of neoadjuvant therapy. The Cam+nab-TP group received camrelizumab (200 mg on day 1), albumin-bound paclitaxel (125 mg/m² on days 1 and 8), and cisplatin (75 mg/m² on day 1). The Cam+TP group was administered camrelizumab (200 mg on day 1), paclitaxel (175 mg/m² on day 1), and cisplatin (75 mg/m² on day 1), while the TP group was given paclitaxel (175 mg/m² on day 1) and cisplatin (75 mg/m² on day 1). Currently, there is no recommended dose of nab-paclitaxel for patients with ESCC, and the dose of nab-paclitaxel used in our study was derived from existing literature on other cancer types and corroborated by our own preliminary findings^20,36,46,47^. For paclitaxel administration, patients received prophylactic agents including dexamethasone, diphenhydramine and H2 receptor antagonists (cimetidine or ranitidine) to prevent hypersensitivity reactions. Cisplatin was administered after dilution in a similar solution, with adequate hydration and diuresis implemented to prevent renal toxicity; no prophylactic antiemetic corticosteroids were used. In this study, the camrelizumab dose was fixed, but chemotherapy doses could be adjusted in response to AEs as detailed in the protocol. Following the completion of neoadjuvant therapy, participants were reassessed for surgery eligibility, which was scheduled to occur within 4–6 weeks later. The surgical procedure involved esophagectomy and lymph node dissection, with the McKeown approach and a total two-field lymphadenectomy being recommended^48^. Both minimally invasive and open esophagectomy were deemed acceptable. For those in the Cam+nab-TP and Cam+TP groups, postoperative adjuvant therapy with camrelizumab (200 mg every 3 weeks) was prescribed for up to 15 cycles.

In this study, all pathological samples were reviewed by a blind independent review committee (BIRC), with the tumor regression grade evaluated according to the Mandard criteria, which involves comparing the proportion of viable tumor to fibrosis confirmed microscopically^49^. The study documented all AEs, postoperative complications and postoperative mortality at 30 and 90 days. AEs were graded and recorded in accordance with the National Cancer Institute Common Terminology Criteria for Adverse Events version 5.0. Postoperative complications were classified as per the standards of the Esophageal Complications Consensus Group^50^, and the severity was graded according to the CD classification^51^.

Furthermore, before treatment initiation, formalin-fixed paraffin-embedded tissue sections acquired through endoscopic biopsy were used to determine PD-L1 expression levels at a central laboratory via immunohistochemistry (PD-L1 IHC 22C3 pharmDx kit, Dako). The assessment of PD-L1 expression involved both CPS and TPS. The CPS is defined as the number of PD-L1 staining cells divided by the total number of viable tumor cells, multiplied by 100; the TPS represents the percentage of viable tumor cells exhibiting membrane staining, assessed in a sample of at least 100 viable tumor cells.

### Endpoints

The study’s dual primary endpoints included the pCR rate, assessed by the BIRC, and EFS, evaluated by the investigators. pCR is defined as the absence of residual tumor at the primary tumor site (tumor regression grade 1) and negative lymph nodes. Secondary endpoints included the MPR rate (defined as less than 10% residual viable tumor cells in the primary tumor) assessed by BIRC, R0 resection rate, ypTNM staging according to the 8th edition of the American Joint Committee on Cancer, OS, DFS, AEs and surgical complications.

### Statistical analysis

This study was designed to demonstrate the superiority of the Cam+nab-TP and Cam+TP groups over the TP group, focusing on the dual primary endpoints of pCR and EFS. The overall type I error rate (α) for the dual primary endpoints was controlled at 0.025 (one-sided) using the graphical approach by Maurer and Bretz, with 0.005 allocated to the pCR hypothesis and 0.02 to the EFS hypothesis tests initially. The comparisons of pCR between the Cam+nab-TP versus TP group, as well as the Cam+TP versus TP group, were sequentially tested at the 0.005 level. If pCR was shown to be superior in the Cam+nab-TP group compared to the TP group, the pCR comparison between the Cam+TP and TP groups would subsequently be tested. Similarly, for EFS comparisons, the following will be tested hierarchically: Cam+nab-TP + Cam+TP versus TP, Cam+nab-TP versus TP, and Cam+TP versus TP. If both pCR rates in the Cam+nab-TP and Cam+TP groups are significantly higher compared with the TP group, the EFS hypotheses will be tested at the 0.025 level; otherwise, they will be tested at the 0.02 level. This report focuses on the final analysis of pCR rates.

The sample size was calculated using NCSS&PASS version 15.0. Assuming pCR rates of 30% for the Cam+nab-TP group, 25% for the Cam+TP group and 9% for the TP group, and using a 1:1:1 randomization ratio with an α level set at 0.005 (one-sided), it was calculated that 111 patients per group would provide at least 93% power to establish the superiority of the Cam+nab-TP group over the TP group, and at least 75% power to demonstrate that the Cam+TP group surpasses the TP group. To accommodate a potential dropout rate of 15%, the study planned to enroll 130 participants in each group. We assumed the median EFS in the TP group to be 30 months. The expected HR for the Cam+nab-TP and Cam+TP groups (combined test groups) compared to the TP group was 0.67. With an initial α set at a one-sided level of 0.02 and with a randomization ratio of 2:1 (Cam+nab-TP and Cam+TP groups versus TP group), a total of 228 events (141 in the combined test groups and 87 in the TP group) are necessary to achieve at least 80% power to detect the superiority of the test groups. Based on an enrollment period of 36 months, a total study duration of 84 months, and an anticipated dropout rate of 15%, approximately 390 patients were required across the three groups. The final analysis of pCR was performed as prespecified when all randomized patients had the opportunity to undergo surgery.

Efficacy analysis adhered to the ITT principle, including all randomized participants. The SS comprised individuals who received at least one dose of the study drug. The 95% CIs for pCR and MPR rates were computed using the Clopper-Pearson method. Adjusted differences in pCR rates and ORs, along with their 95% CIs between the Cam+nab-TP and TP groups, as well as between the Cam+TP and TP groups, were calculated using the Mantel-Haenszel method, stratified by clinical stage (I/II versus III/IVA). The pCR between Cam+nab-TP and TP groups as well as between Cam+TP and TP groups were compared by using stratified Cochran-Mantel-Haenszel test with the same stratification factor. Post hoc subgroup analyses of pCR, based on baseline characteristics, were performed, with rate differences estimated and presented in an unadjusted forest plot for each subgroup comparison. All statistical analyses were conducted using SAS software version 9.4. Data collection was performed using Bioknow electronic data capture system.

### Reporting summary

Further information on research design is available in the Nature Portfolio Reporting Summary linked to this article.

## Online content

Any methods, additional references, Nature Portfolio reporting summaries, source data, extended data, supplementary information, acknowledgements, peer review information; details of author contributions and competing interests; and statements of data and code availability are available at 10.1038/s41591-024-03064-w.

## Supplementary information

Supplementary Information Supplementary Tables 1–5, study protocol and statistical analysis plan.

Reporting Summary

## Data Availability

Due to intellectual property and confidentiality obligations, individual deidentified participant data that underlie the results reported in this article can be requested 24 months after study completion. Qualified researchers must submit a proposal to the corresponding author at liyin@cicams.ac.cn, outlining the reasons for requesting the data. The leading clinical site and sponsor will review the request to ensure compliance with intellectual property and confidentiality obligations and will respond within two weeks. A signed data access agreement with the sponsor is required before any data can be shared. The study protocol and statistical analysis plan are available alongside the published article.

## Code availability

No custom code was used for data analysis in this study.
