# Supplementary material for: Neoadjuvant chemotherapy with or without camrelizumab in resectable esophageal squamous cell carcinoma: the randomized phase 3 ESCORT-NEO/NCCES01 trial
Source: Nat Med. 2024 Jul 2;30(9):2549–57. doi: 10.1038/s41591-024-03064-w (PMC11405280; doi:10.1038/s41591-024-03064-w)
Supplement: Supplementary file 1 — Supplementary Tables 1–5, study protocol and statistical analysis plan. [file 41591_2024_3064_MOESM1_ESM.pdf]

nature medicine

Supplementary information https://doi.org/10.1038/s41591-024-03064-w

Neoadjuvant chemotherapy with or without camrelizumab in resectable esophageal squamous cell carcinoma: the randomized phase 3 ESCORT-NEO/NCCES01 trial

In the format provided by the authors and unedited

Supplementary Table 1. Pathological outcomes in patients undergoing esophagectomy

Variables	Cam+nab-TP (n=114)	Cam+TP (n=116)	TP (n=103)
TRG in primary tumor, n (%)			
TRG 1	47 (41.2)	23 (19.8)	7 (6.8)
TRG 2	24 (21.1)	21 (18.1)	12 (11.7)
TRG 3	30 (26.3)	36 (31.0)	32 (31.1)
TRG 4	13 (11.4)	34 (29.3)	47 (45.6)
TRG 5	0	2 (1.7)	5 (4.9)
ypN stage, n (%)			
ypN0	72 (63.2)	63 (54.3)	47 (45.6)
ypN1	24 (21.1)	30 (25.9)	29 (28.2)
ypN2	15 (13.2)	17 (14.7)	22 (21.4)
ypN3	3 (2.6)	6 (5.2)	5 (4.9)
ypTNM stage, n (%)			
I	58 (50.9)	46 (39.7)	27 (26.2)
II	14 (12.3)	16 (13.8)	20 (19.4)
IIIA	16 (14.0)	15 (12.9)	14 (13.6)
IIIB	23 (20.2)	32 (27.6)	35 (34.0)
IVA	3 (2.6)	6 (5.2)	7 (6.8)
IVB	0	1 (0.9)	0

TRG, tumor regression grade.

Supplementary Table 2. Surgical complications according to Clavien-Dindo classification in all groups

Events, n (%)	Cam+nab-TP (n=114)		Cam+TP (n=116)		TP (n=103)	
	Any grade	Grade ≥3	Any grade	Grade ≥3	Any grade	Grade ≥3
Any events	39 (34.2)	7 (6.1)	45 (38.8)	14 (12.1)	33 (32.0)	7 (6.8)
Pneumonia	12 (10.5)	0	21 (18.1)	1 (0.9)	15 (14.6)	2 (1.9)
Recurrent laryngeal nerve injury	11 (9.6)	0	11 (9.5)	1 (0.9)	9 (8.7)	1 (1.0)
Dysrhythmia	7 (6.1)	0	2 (1.7)	0	3 (2.9)	0
Pleural effusion	3 (2.6)	3 (2.6)	12 (10.3)	7 (6.0)	7 (6.8)	3 (2.9)
Anastomotic leak	3 (2.6)	1 (0.9)	5 (4.3)	2 (1.7)	6 (5.8)	1 (1.0)
Conduit necrosis	2 (1.8)	0	1 (0.9)	0	1 (1.0)	0
Respiratory failure	1 (0.9)	1 (0.9)	0	0	1 (1.0)	1 (1.0)
Intrathoracic abscess	1 (0.9)	1 (0.9)	0	0	1 (1.0)	0
Delirium	1 (0.9)	0	0	0	1 (1.0)	0
Septic shock	0	0	3 (2.6)	3 (2.6)	0	0
Atelectasis	0	0	1 (0.9)	0	1 (1.0)	1 (1.0)
Chylous leak	0	0	0	0	2 (1.9)	0
Delayed conduit emptying	0	0	0	0	2 (1.9)	1 (1.0)

Wound dehiscence	0	0	1 (0.9)	1 (0.9)	0	0
Congestive heart failure	0	0	0	0	1 (1.0)	1 (1.0)
Anastomotic stenosis	0	0	1 (0.9)	0	0	0
Urine retention	1 (0.9)	1 (0.9)	0	0	0	0
Myocardial infarction	0	0	0	0	1 (1.0)	1 (1.0)
Acute respiratory distress syndrome	0	0	1 (0.9)	0	0	0
Postoperative wound infection	0	0	0	0	1 (1.0)	1 (1.0)
Mechanical bowel obstruction	0	0	1 (0.9)	1 (0.9)	0	0
Sudden death with unknown cause	1 (0.9)	1 (0.9)	0	0	0	0
Pneumothorax	1 (0.9)	0	0	0	0	0
Electrolyte imbalance	0	0	1 (0.9)	0	0	0
Subcutaneous haemorrhage	1 (0.9)	0	0	0	0	0
Subcutaneous emphysema	1 (0.9)	0	0	0	0	0
Complications of jejunostomy tube	1 (0.9)	0	0	0	0	0
Sinus tachycardia	0	0	1 (0.9)	0	0	0
Mediastinal abscess	0	0	0	0	1 (1.0)	0
Gastrointestinal haemorrhage	0	0	1 (0.9)	1 (0.9)	0	0

Soft tissue infection	1 (0.9)	0	0	0	0	0

Supplementary Table 3. Summary of preoperative adverse events

Events, n (%)	Cam+nab-TP (n=132)	Cam+TP (n=130)	TP (n=125)
TEAE	125 (94.7)	118 (90.8)	108 (86.4)
Grade ≥3 TEAE	46 (34.8)	41 (31.5)	37 (29.6)
TEAE leading to camrelizumab discontinuation	1 (0.8)	1 (0.8)	-
TEAE leading to chemotherapy discontinuation	4 (3.0)	5 (3.8)	1 (0.8)
TEAE leading to death	0	1 (0.8)	0
TRAE	124 (93.9)	108 (83.1)	104 (83.2)
Grade ≥3 TRAE	45 (34.1)	38 (29.2)	36 (28.8)
TRAE leading to camrelizumab discontinuation	1 (0.8)[a]	1 (0.8)[b]	-
TRAE leading to chemotherapy discontinuation	4 (3.0)	5 (3.8)	1 (0.8)
TRAE leading to death	0	1 (0.8)[b]	0
SAE	10 (7.6)	12 (9.2)	7 (5.6)
irAE	36 (27.3)	32 (24.6)	0
Grade ≥3 irAE	6 (4.5)	5 (3.8)	0

a Preoperative acute kidney injury; b Subacute hepatic failure.

TEAE, treatment emergent adverse event; TRAE, treatment-related adverse event; SAE, serious adverse event; irAE, immune-related adverse event.

Supplementary Table 4. Preoperative TRAE in any group

Events, n (%)	Cam+nab-TP (n=132)		Cam+TP (n=130)		TP (n=125)	
	Any grade	Grade ≥3	Any grade	Grade ≥3	Any grade	Grade ≥3
Any TRAE	124 (93.9)	45 (34.1)	108 (83.1)	38 (29.2)	104 (83.2)	36 (28.8)
White blood cell count decreased	68 (51.5)	15 (11.4)	51 (39.2)	13 (10.0)	41 (32.8)	6 (4.8)
Neutrophil count decreased	61 (46.2)	33 (25.0)	48 (36.9)	26 (20.0)	38 (30.4)	29 (23.2)
Anemia	41 (31.1)	0	30 (23.1)	2 (1.5)	23 (18.4)	1 (0.8)
Nausea	40 (30.3)	2 (1.5)	28 (21.5)	0	33 (26.4)	1 (0.8)
Alopecia	36 (27.3)	0	30 (23.1)	0	25 (20.0)	0
Lymphocyte count decreased	21 (15.9)	7 (5.3)	12 (9.2)	3 (2.3)	10 (8.0)	1 (0.8)
Vomiting	20 (15.2)	1 (0.8)	9 (6.9)	0	18 (14.4)	2 (1.6)
Platelet count decreased	15 (11.4)	0	8 (6.2)	2 (1.5)	5 (4.0)	0
Hypokalemia	15 (11.4)	2 (1.5)	3 (2.3)	0	7 (5.6)	1 (0.8)
Hyponatremia	14 (10.6)	2 (1.5)	6 (4.6)	2 (1.5)	4 (3.2)	2 (1.6)
Fatigue	13 (9.8)	0	16 (12.3)	0	11 (8.8)	0
Creatinine increased	13 (9.8)	1 (0.8)	10 (7.7)	0	11 (8.8)	0
Rash	12 (9.1)	0	14 (10.8)	1 (0.8)	8 (6.4)	0

Anorexia	12 (9.1)	0	13 (10.0)	0	7 (5.6)	0
Reactive cutaneous capillary endothelial proliferation	11 (8.3)	0	13 (10.0)	0	0	0
Alanine aminotransferase increased	10 (7.6)	1 (0.8)	11 (8.5)	2 (1.5)	6 (4.8)	0
Diarrhea	8 (6.1)	1 (0.8)	9 (6.9)	1 (0.8)	6 (4.8)	0
Aspartate aminotransferase increased	6 (4.5)	0	9 (6.9)	2 (1.5)	3 (2.4)	0
Myalgia	4 (3.0)	0	6 (4.6)	0	9 (7.2)	0
Arthralgia	4 (3.0)	0	9 (6.9)	0	5 (4.0)	0
Dysesthesia	3 (2.3)	0	4 (3.1)	0	7 (5.6)	0
Hypothyroidism	6 (4.5)	0	1 (0.8)	0	0	0
Blood bilirubin increased	5 (3.8)	1 (0.8)	5 (3.8)	0	1 (0.8)	0
GGT increased	4 (3.0)	0	1 (0.8)	0	2 (1.6)	0
Weight loss	4 (3.0)	0	1 (0.8)	0	2 (1.6)	0
Mononuclear cell count decreased	4 (3.0)	0	1 (0.8)	0	2 (1.6)	0
Fever	4 (3.0)	1 (0.8)	2 (1.5)	1 (0.8)	1 (0.8)	0
Hypoalbuminemia	3 (2.3)	0	5 (3.8)	0	4 (3.2)	0
Hiccups	3 (2.3)	0	6 (4.6)	0	2 (1.6)	0

Hyperthyroidism	3 (2.3)	0	6 (4.6)	0	0	0
Hypoproteinemia	3 (2.3)	0	1 (0.8)	0	2 (1.6)	0
Lactate dehydrogenase increased	3 (2.3)	0	2 (1.5)	0	0	0
Mouth ulcer	3 (2.3)	0	1 (0.8)	0	0	0
Serum amylase increased	3 (2.3)	0	1 (0.8)	0	0	0
Acute kidney injury	3 (2.3)	2 (1.5)	0	0	0	0
Decreased receptivity	2 (1.5)	0	6 (4.6)	0	4 (3.2)	0
Dyspepsia	2 (1.5)	0	4 (3.1)	0	2 (1.6)	0
Hypochloremia	2 (1.5)	0	3 (2.3)	0	1 (0.8)	0
Constipation	2 (1.5)	0	2 (1.5)	0	2 (1.6)	0
Pruritus	2 (1.5)	0	3 (2.3)	0	1 (0.8)	0
Hypocalcemia	2 (1.5)	0	2 (1.5)	0	1 (0.8)	0
dizziness	2 (1.5)	0	3 (2.3)	0	0	0
Electrolyte imbalance	2 (1.5)	1 (0.8)	0	0	3 (2.4)	1 (0.8)
Elevated urea	2 (1.5)	0	2 (1.5)	0	1 (0.8)	0
Alkaline phosphatase increased	2 (1.5)	0	3 (2.3)	0	0	0
Cough	2 (1.5)	0	0	0	2 (1.6)	0

Flatulence	2 (1.5)	0	2 (1.5)	0	0	0
Pulmonary inflammation	2 (1.5)	0	1 (0.8)	0	1 (0.8)	0
Hyperuricemia	2 (1.5)	0	1 (0.8)	0	1 (0.8)	0
Gum infection	2 (1.5)	0	1 (0.8)	0	1 (0.8)	0
Headache	2 (1.5)	0	1 (0.8)	0	0	0
Hyperglycemia	2 (1.5)	1 (0.8)	1 (0.8)	0	0	0
Eosinophils decreased	2 (1.5)	0	0	0	0	0
Retching	2 (1.5)	0	0	0	0	0
Gastrointestinal diseases	2 (1.5)	0	0	0	0	0
Hypophosphatemia	2 (1.5)	0	1 (0.8)	0	0	0
Phlebitis	2 (1.5)	0	0	0	0	0
Belching	1 (0.8)	0	6 (4.6)	0	3 (2.4)	0
Insomnia	1 (0.8)	0	4 (3.1)	0	3 (2.4)	0
Pain in the limbs	1 (0.8)	0	1 (0.8)	0	6 (4.8)	0
Pain	1 (0.8)	0	1 (0.8)	0	2 (1.6)	0
Hydroxybutyrate dehydrogenase increased	1 (0.8)	0	2 (1.5)	1 (0.8)	0	0
Laryngeal pain	1 (0.8)	0	0	0	2 (1.6)	0

Hepatic dysfunction	1 (0.8)	1 (0.8)	0	0	2 (1.6)	1 (0.8)
Gastroesophageal reflux disease	1 (0.8)	0	1 (0.8)	0	1 (0.8)	0
Abdominal discomfort	1 (0.8)	0	2 (1.5)	0	0	0
Upper respiratory infection	1 (0.8)	0	0	0	1 (0.8)	0
Upper gastrointestinal hemorrhage	1 (0.8)	0	1 (0.8)	0	0	0
Febrile neutropenia	1 (0.8)	0	1 (0.8)	1 (0.8)	0	0
Hypersomnia	1 (0.8)	0	0	0	1 (0.8)	0
Hyperhidrosis	1 (0.8)	0	1 (0.8)	0	0	0
Red blood cell count decreased	1 (0.8)	0	0	0	1 (0.8)	0
Thyroid stimulating hormone decreased	1 (0.8)	0	1 (0.8)	0	0	0
Gutathione reductase activity increased	1 (0.8)	0	0	0	1 (0.8)	0
Hypersensitivity	1 (0.8)	0	0	0	1 (0.8)	0
Hyperkalemia	1 (0.8)	0	0	0	1 (0.8)	1 (0.8)
Hypoglycemia	1 (0.8)	0	0	0	0	0
Immune mediated pulmonary disease	1 (0.8)	0	0	0	0	0
Arthritis	1 (0.8)	0	0	0	0	0
Dry mouth	1 (0.8)	0	0	0	0	0

Sleepiness	1 (0.8)	0	0	0	0	0
Infectious pneumonia	1 (0.8)	1 (0.8)	0	0	0	0
Gastritis	1 (0.8)	0	0	0	0	0
Bronchitis	1 (0.8)	0	0	0	0	0
Activated partial thromboplastin time prolonged	1 (0.8)	0	0	0	0	0
Ophthalmoplegia	1 (0.8)	0	0	0	0	0
Sinus tachycardia	1 (0.8)	0	0	0	0	0
Conjugated bilirubin increased	1 (0.8)	0	0	0	0	0
Creatinine clearance rate decreased	1 (0.8)	1 (0.8)	0	0	0	0
Cardiac troponin I increased	1 (0.8)	0	0	0	0	0
Chest pain	1 (0.8)	0	0	0	0	0
Dehydration	1 (0.8)	0	0	0	0	0
Hypochondriac pain	1 (0.8)	0	0	0	0	0
Abdominal pain	1 (0.8)	0	0	0	0	0
Urea reduction	1 (0.8)	0	0	0	0	0
CPK isoenzyme increased	1 (0.8)	0	0	0	0	0
Glutamate dehydrogenase increased	1 (0.8)	0	0	0	0	0

Hyperlipidemia	1 (0.8)	0	0	0	0	0
Drug induced hypersensitivity	0	0	2 (1.5)	1 (0.8)	1 (0.8)	0
Upper abdominal pain	0	0	2 (1.5)	0	0	0
Urinary tract infection	0	0	1 (0.8)	0	1 (0.8)	0
Edema	0	0	1 (0.8)	0	1 (0.8)	0
Toothache	0	0	1 (0.8)	0	1 (0.8)	0
Chest discomfort	0	0	1 (0.8)	0	1 (0.8)	0
Brain natriuretic peptide increased	0	0	1 (0.8)	0	1 (0.8)	0
Thyroid stimulating hormone increased	0	0	2 (1.5)	0	0	0
Cholesterol high	0	0	1 (0.8)	0	1 (0.8)	0
Pseudohallucination	0	0	0	0	1 (0.8)	0
Dyspnea	0	0	1 (0.8)	0	0	0
Peripheral edema	0	0	1 (0.8)	0	0	0
Urinary white blood cell positivity	0	0	1 (0.8)	0	0	0
Acute hepatic failure	0	0	1 (0.8)	1 (0.8)	0	0
Dysuria	0	0	0	0	0	0
Lymphocyte percentage decreased	0	0	1 (0.8)	0	1 (0.8)	0

Flushing	0	0	1 (0.8)	0	1 (0.8)	0
Skin peeling	0	0	1 (0.8)	0	0	0
Mediastinal fistula	0	0	1 (0.8)	0	0	0
Conjunctival hemorrhage	0	0	1 (0.8)	0	0	0
Ear pain	0	0	1 (0.8)	0	0	0
Tinnitus	0	0	1 (0.8)	0	0	0
Estimated glomerular filtration rate decreased	0	0	1 (0.8)	0	0	0
Abdominal distension	0	0	0	0	1 (0.8)	0
Autoimmune thyroiditis	0	0	1 (0.8)	0	0	0
Autoimmune hepatitis	0	0	1 (0.8)	1 (0.8)	0	0
CPK increased	0	0	1 (0.8)	0	0	0
Eating disorders	0	0	0	0	1 (0.8)	0
Hypertriglyceridemia	0	0	0	0	1 (0.8)	0
Hypertension	0	0	1 (0.8)	0	0	0

TRAE, treatment-related adverse events, GGT: gamma-glutamyl transferase, CPK: creatine phosphokinase, CPK isoenzyme, creatine phosphokinase isoenzyme.

Supplementary Table 5. Preoperative irAE in at least two patients in all groups

Events, n (%)	Cam+nab-TP (n=132)		Cam+TP (n=130)	
	Any grade	Grade ≥3	Any grade	Grade ≥3
Any irAE	36 (27.3)	6 (4.5)	32 (24.6)	5 (3.8)
Reactive cutaneous capillary endothelial proliferation	11 (8.3)	0	13 (10.0)	0
Rash	7 (5.3)	0	9 (6.9)	1 (0.8)
Creatinine increased	7 (5.3)	1 (0.8)	2 (1.5)	0
Hypothyroidism	6 (4.5)	0	1 (0.8)	0
Hyperthyroidism	3 (2.3)	0	3 (2.3)	0
White blood cell count decreased	2 (1.5)	1 (0.8)	0	0
Pruritus	1 (0.8)	0	3 (2.3)	0
Neutrophil count decreased	1 (0.8)	0	1 (0.8)	1 (0.8)
Urea increased	1 (0.8)	0	1 (0.8)	0
Hyperglycemia	1 (0.8)	1 (0.8)	1 (0.8)	0
Amylase increased	1 (0.8)	0	1 (0.8)	0
Alanine aminotransferase increased	0	0	3 (2.3)	2 (1.5)
Diarrhea	0	0	2 (1.5)	1 (0.8)

irAE, immune-related adverse event.

A randomized, open-label, parallel-controlled clinical study of camrelizumab combined with neoadjuvant chemotherapy versus neoadjuvant chemotherapy alone for resectable locally advanced thoracic esophageal squamous cell carcinoma

Study Protocol

Version No.: 3.0

Version Date: April 15, 2022

Principal investigator: Professor Yin Li

Study Institution: Cancer Hospital Chinese Academy of Medical Sciences

Version History/Revision History

Document Version	Version Date	Revision Record
V1.0	September 15, 2020	Not applicable
V2.0	July 02, 2021	**Secondary endpoints:**<br>Listed EFS as a key secondary endpoint, and included it in the statistical hypotheses.<br>**Exploratory endpoints:**<br>Added PRO as an exploratory endpoint.<br>**Safety assessment criteria:**<br>Included the description of grading according to Clavien-Dindo (CD) criteria for the surgical complications.<br>**Sample size**<br>Included pCR and EFS in the statistical hypotheses, with the plan to enroll 390 subjects after calculation.<br>**Detail correction**
V3.0	April 15, 2022	• **Primary objective:**<br>Adjusted the original secondary objective EFS to one of the primary objectives, specifically "to compare the Event-Free Survival (EFS) between the test group and the control group";<br>• **Secondary objective:**<br>Removed EFS from the secondary objective and adjusted it to the primary objective;<br>• **Primary endpoints:**<br>Adjusted the original key secondary endpoint EFS to the primary endpoints, and refined the definition of EFS;<br>• **Secondary endpoints:**<br>Removed the key secondary endpoint EFS; added new secondary efficacy endpoints MPR, and ypTNM staging;<br>• **Regimen:**<br>Adjusted from the original "until... new anti-tumor therapy" to "until... other systemic anti-tumor therapy"; adjusted from the original "maximum treatment duration of camrelizumab should not exceed 1 year" to "maximum treatment duration of camrelizumab should be up to a total of 17 doses pre- and post-surgery", clarified the duration of postoperative adjuvant therapy for the test group, without actually increasing the drug exposure of the subjects;<br>• **Postoperative adjuvant therapy:**<br>Added the following: For subjects who are pathologically assessed as non-R0 resected (R1/R2) after surgery, other treatment options determined by the investigator may be accepted according to clinical practice, but subsequent safety follow-up, tumor progression/recurrence follow-up, and survival follow-up should still be continued.<br>• **Radiological assessment:**<br>Added the recommendation for the inclusion of

		neck ultrasound for the diagnosis and differential diagnosis of metastatic lesions such as cervical lymph nodes.<br>Changed from the original "…until radiological progression/recurrence, new anti-tumor therapy, withdrawal of informed consent, loss to follow-up, or death, whichever occurs first" to "…until radiological progression/recurrence, withdrawal of informed consent, loss to follow-up, or death, whichever occurs first";<br>Removed "new anti-tumor therapy" in radiological assessment.<br>• **Follow-up:**<br>Clarified that the follow-up period includes safety follow-up, tumor progression/recurrence follow-up, and survival follow-up;<br>Clarified that subjects who discontinue treatment for reasons other than disease progression/recurrence need to continue radiological follow-up until radiological progression/recurrence, withdrawal of informed consent, loss to follow-up, or death.<br>• **Stratification factors:**<br>Deleted site stratification;<br>• **Statistical hypotheses**<br>Modified the statistical hypotheses, adjusted to a statistical design of α allocation , see details in Protocol 11.2;<br>• **Sample size calculation**<br>Modified the description related to sample size calculation according to the statistical hypotheses;<br>• **Analysis sets**<br>Defined ITT as the primary efficacy set;<br>Add a new surgery analysis set, mainly for the assessment of surgery safety;<br>• **Interim analysis**<br>Modified α allocation for 11.4.4 Interim Analysis<br>• **Multiplicity**<br>Added 11.4.5 Multiplicity as a result of the modification of statistical hypotheses, which involved testing of multiplicity hypotheses.

Protocol Signature Page

I, as a participating physician/statistical analyst, have read the protocol of this study.

I have had thorough discussion with the study director regarding the purpose of this study and the contents of the protocol.

I agree to conduct the study according to this protocol and the guidance of Good Clinical Practice (GCP), and to adhere to ethical standards.

I agree to keep this protocol confidential, not to disclose this protocol to any third party, and to use this protocol only for the purpose of conducting this study.

I understand that I will be notified in writing if this study is terminated or suspended for any reason at any time. Similarly, if I decide to withdraw from this study, I will immediately notify the responsible institution of this study and the principal investigator (sponsor) in writing.

Version No.: 3.0

Version Date: April 15, 2022

Study site name: ____________________________

Investigator signature: ____________________________

Date: ____________________________

Table of Contents

Version History/Revision History i
Protocol Signature Page iii
Protocol Synopsis 1
Schedule of Activities 12
1. Study Background and Scientific Rationale 19
1.1 Epidemiology and Treatment Status of Esophageal Cancer 19
1.2 Immune Checkpoint Therapy 20
1.3 Scientific Rationale for the Study 20
2. Study Objectives and Endpoints 21
2.1 Study Objectives 21
2.2 Study Endpoints 22
3. Study Design 23
3.1 Overall design 23
3.2 Methods to Reduce Bias 26
3.3 Study Duration 26
4. Study Population 27
4.1 Inclusion Criteria 27
4.2 Exclusion Criteria 27
4.3 Discontinuation of Treatment/Withdrawal from Study/Study Termination 29
5. Investigational Medicinal Products 30
5.1 Overview of Study Drugs 30
5.2 Packaging and Labeling 31
5.3 Preparation of Study Drugs 31
5.4 Treatment Plan 31
5.5 Dose Modification and Delay 34
5.6 Concomitant Medications and Concomitant Therapies 40
6. Study Procedures 42
6.1 Screening Period 42
6.2 Treatment Period 42
6.3 End of Treatment and Safety Follow-up Period 43
6.4 Survival Follow-up 43
6.5 Tumor Progression/Recurrence Follow-up 43
6.6 Unscheduled Visits 43
6.7 Collection of Tissue Samples and Blood Samples 44
7. Efficacy Evaluation 44
7.1 Efficacy Parameters 44
7.2 Efficacy Assessment 44
8. Safety Evaluation 46
8.1 Safety Parameters 46

8.2 Safety Assessment Criteria 46
8.3 Laboratory Safety Evaluation 47
8.4 Others 47
9. Patient-reported Outcomes 47
9.1 EORTC QLQ-C30 48
9.2 EORTC QLQ-OES18 48
10. Adverse Event Reporting 49
10.1 Adverse Events 49
10.2 Serious Adverse Events 50
10.3 Collection/Follow-up of Adverse Events and Reporting of Serious Adverse Events 51
10.4 Pregnancy Reporting 52
10.5 Non-serious Adverse Events/Reactions 52
10.6 Disease Progression and Death Reporting 52
10.7 Immune-related Adverse Events 53
10.8 Hepatic Function Abnormal 53
11. Data Analysis/Statistical Methods 54
11.1 Sample Size 54
11.2 Statistical Hypotheses 54
11.3 Analysis Sets 55
11.4 Statistical Analysis Methods 55
12. Data Management 58
12.1 Study Data Management 58
12.2 Data Monitoring 59
12.3 Protocol Deviations 59
13. Raw Data and Original Documents 60
14. Quality Assurance and Quality Control 60
15. Ethics and Informed Consent 61
15.1 Ethical Codes 61
15.2 Institutional Review Board/Independent Ethics Committee 61
15.3 Informed Consent 61
16. References 62

Protocol Synopsis

**Study Title**	A randomized, open-label, parallel-controlled clinical study of camrelizumab combined with neoadjuvant chemotherapy versus neoadjuvant chemotherapy alone for resectable locally advanced thoracic esophageal squamous cell carcinoma
**Version No.**	Version 3.0
**Version Date**	April 15, 2022
**Sponsor/Leading Unit**	Cancer Hospital Chinese Academy of Medical Sciences
**Principal Investigator**	Professor Yin Li
**Study Nature**	Investigator-initiated clinical study
**Participating Sites**	Approximately 20 sites
**Study Objectives**	**Primary objective:**<br>• To compare the perioperative regimen of camrelizumab combined with neoadjuvant chemotherapy and postoperative camrelizumab adjuvant therapy versus neoadjuvant chemotherapy with paclitaxel and cisplatin for patients with resectable esophageal squamous cell carcinoma in terms of pathologcal complete response (pCR) and Event-Free Survival (EFS).
	**Secondary objective:**<br>• To compare the perioperative regimen of camrelizumab combined with neoadjuvant chemotherapy and postoperative camrelizumab adjuvant therapy versus neoadjuvant chemotherapy with paclitaxel and cisplatin for patients with resectable esophageal squamous cell carcinoma in terms of major pathological response (MPR), R0 resection rate, pathological stage after neoadjuvant therapy (ypTNM stage), disease-free survival (DFS), overall survival (OS), and safety and tolerability.
	**Exploratory objectives:**<br>• To explore the quality of life of patients with resectable esophageal squamous cell carcinoma treated with the perioperative regimen of camrelizumab combined with neoadjuvant chemotherapy and postoperative camrelizumab adjuvant therapy compared to the neoadjuvant chemotherapy with paclitaxel and cisplatin;<br>• To evaluate the relationship between biomarkers in tumor tissues and response;
**Study Endpoints**	**Primary endpoints:**<br>• pCR assessed by Blinded Independent Review Committee (BIRC), defined as the proportion of subjects with no residual tumor in the primary tumor site (Mandard grade 1) and histologically negative lymph nodes;<br>• EFS assessed by the investigator according to RECIST 1.1, defined as the time from randomization to the occurrence of any of the following events, whichever comes first:<br>➢ Radiographic tumor progression assessed by RECIST 1.1;<br>➢ Tumor recurrence assessed by radiology or tissue biopsy, including local recurrence or distant metastasis (for subjects with no residual tumor after surgery);<br>➢ Death due to any cause;<br>Note: A second primary malignancy, or radiological progression occurring during the neoadjuvant phase that does not affect the radical resection of esophageal cancer, is not considered an EFS event.
	**Secondary endpoints:**<br>**Efficacy endpoints:**<br>• MPR assessed by BIRC: The percentage of subjects with <10% residual tumor in the primary tumor site;<br>• R0 resection rate;<br>• Pathological stage after neoadjuvant therapy (ypTNM stage) based on the 8th edition of AJCC;

	• OS: The time from randomization to death due to any cause;<br>• DFS: The time from the date of surgery who completed R0 resection to local or distant recurrence, or death due to any cause, whichever occurs first;<br>**Safety:**<br>• Adverse events (AEs): Incidence and grade (including serious adverse events [SAEs] and immune-related adverse events [irAEs]), determined according to the NCI-CTCAE 5.0 criteria;<br>• Surgery safety: Record surgical complications during the 30 days after surgery or during the hospitalization period, length of hospital stay, reoperation rate, and mortality rates at 30 days post-surgery and 90 days post-surgery, with the severity of surgical complications graded according to the Clavien-Dindo (CD) criteria;<br>**Exploratory endpoints:**<br>• Patient-reported outcomes (PRO): Quality of life and changes from baseline assessed from baseline to survival follow-up according to the European Organization for Research and Treatment of Cancer Quality of Life Questionnaire-C30 (EORTC QLQ-C30) and the Supplementary Questionnaire for Patients with Esophageal Cancer (EORTC QLQ-OES18);<br>• The relationship between potential biomarkers in tumor tissues (including but not limited to baseline PD-L1 expression (IHC, PD-L1 IHC 22C3 pharmDx kit, Dako, The assessment of PD-L1 expression involved both CPS and TPS), MMR status, TMB, EBER) and response;
**Study Subjects**	Patients with previously untreated, histopathologically or cytologically confirmed resectable locally advanced thoracic esophageal squamous cell carcinoma (clinical stage T1b-3N1-3M0 or T3N0M0, according to the 8th edition of AJCC)
**Study Design**	This study is a multicenter, randomized, open, parallel-controlled clinical study in China to compare the efficacy and safety of camrelizumab combined with albumin-bound paclitaxel and cisplatin (Group A) or camrelizumab combined with paclitaxel and cisplatin (Group B) versus paclitaxel and cisplatin (Group C) in patients with histopathologically or cytologically confirmed resectable locally advanced thoracic esophageal squamous cell carcinoma.<br><br>Eligible patients will be randomized in a 1:1:1 ratio to test group A, test group B, or control group C, with pCR assessed by BIRC and EFS assessed by the investigator as primary endpoints, and 390 patients are planned to be enrolled. A stratified block randomization method will be used in this study, with stratification factors including:<br>• Clinical stage (Stage I/II vs. Stage III vs. Stage IVa)<br><br>The study includes a screening period (no more than 21 days, from the signing of informed consent form [ICF] to the first dose), a treatment period (including neoadjuvant therapy, surgery, and postoperative adjuvant therapy) and a follow-up period (including safety follow-up, tumor progression/recurrence follow-up, and survival follow-up).<br><br>**<u>Neoadjuvant therapy:</u>**<br>• **Test group (Group A):** Camrelizumab combined with albumin-bound paclitaxel and cisplatin, 2 cycles, followed by surgical resection;<br>• **Test group (Group B):** Camrelizumab combined with paclitaxel and cisplatin, 2 cycles, followed by surgical resection;<br>• **Control group (Group C):** Paclitaxel and cisplatin, 2 cycles, followed by surgical resection;<br>During the study treatment period, a dosing time window of ± 3 days is allowed, but within 3 days before each dose, except for necessary radiological

examinations, subjects should complete laboratory tests and physical examinations (as needed), ECOG scoring and other safety assessments to ensure tolerance before continuing with the study treatment (if the laboratory tests of screening period are completed within the protocol-specified pre-randomization time, then there is no need to retest before the first dose). The safety of subjects is continuously assessed throughout the study process.

Surgery:

- At any time during regular tumor assessment or neoadjuvant therapy, if disease progression is observed in a subject but the investigator judges the tumor to be still resectable, without distant metastasis, and the subject still meets the eligibility criteria for study treatment and evaluation, they may continue to receive surgery and subsequent treatment.
- Subjects who discontinue neoadjuvant therapy due to disease progression and no longer undergo surgery will discontinue the subsequent study treatment procedures and receive other treatment plans determined by the investigator. However, subjects will continue to receive safety follow-up and survival follow-up.
- For subjects whose tumor does not progress during neoadjuvant therapy but cannot undergo surgery due to other reasons, the investigator may determine the next step of the treatment plan with the subject. Subjects will still continue to receive safety follow-up and survival follow-up.
- Subjects will be re-assessed by the investigator prior to surgery. Preoperative visits and related assessments should be performed within 14 days before surgery, and follow the treatment principles of each site. Surgery should be performed within 4-6 weeks of last dose. If surgical treatment exceeds 6 weeks of the last dose of neoadjuvant therapy, the preoperative visits and related assessments will be repeated within 14 days before surgery. After surgery, the surgical specimens will be assessed for pathological response by each site and BIRC.
- As for resection of esophageal cancer + complete thoracoabdominal two-field lymphadenectomy, the recommended surgical method is Mckeown procedure, and the complete thoracoabdominal two-field dissection is recommended for the lymphadenectomy.

Postoperative adjuvant therapy:

- **Test groups (Groups A and B):** Camrelizumab will be administered every 3 weeks as a dosing cycle, until radiological disease recurrence or metastasis, intolerable toxicity, initiation of other systemic anti-tumor therapy, withdrawal at the subject's own request, or withdrawal required by the investigator; the first dose of camrelizumab after surgery should be administered within 4-6 weeks postoperatively; during the entire study period, camrelizumab is administered for a maximum of 17 doses in total before and after surgery.

- **Control group (Group C):** Subjects in the control group will receive regular follow-up observation until disease recurrence or metastasis, withdrawal at the subject's own request, or withdrawal required by the investigator;

During the study treatment period, a dosing time window of ±3 days is allowed, but within 3 days before each dose, subjects should complete laboratory tests and/or necessary physical examinations, ECOG scoring, and other safety assessments to ensure they can still tolerate the study treatment. The safety of subjects is continuously assessed throughout the study process. Upon discontinuation of treatment, the subjects are required to undergo a comprehensive examination, including vital signs, physical examination, laboratory tests, and radiological tumor assessment.

For subjects who are pathologically assessed as non-R0 resected (R1/R2) after surgery, other treatment options determined by the investigator may be accepted according to clinical practice, but subsequent safety follow-up, tumor progression/recurrence follow-up, and survival follow-up should still be continued.

Radiological assessment:

Regular radiological response assessments will be performed according to RECIST 1.1 criteria, which include enhanced CT scans of the neck/chest/abdomen (CT scan slice thickness ≤5 mm) or enhanced MRI scans; it is recommended to add neck ultrasound for the diagnosis and differential diagnosis of metastatic lesions such as cervical lymph nodes; those suspected of having brain metastasis must also undergo enhanced brain MRI or enhanced CT to exclude brain metastasis; when metastases to bone are confirmed or clinically suspected, a bone scan is required; unless otherwise specified, the permissible window period for radiological examinations is ± 7 days. When disease progression is suspected (such as worsening of symptoms), unscheduled radiological examinations may be conducted.

- **At screening**, all subjects should undergo tumor radiological assessments; the tumor baseline assessments can be relaxed to within 3 weeks before randomization, and CT/MRI results obtained before signing the ICF can be used for the tumor assessment at screening if they meet the requirements of this protocol, and esophageal ultrasound endoscopy (EUS) should be added when making clinical staging judgments;
- **After randomization,** the conditions for radiological examination should be the same as the baseline (including scan slice thickness, contrast agents, etc.). Tumor radiological assessments should be performed within 14 days before surgery (i.e., within 3-4 weeks after the last dose of neoadjuvant therapy, or within 14 days before surgery if surgery exceeds 6 weeks after the last dose of neoadjuvant therapy), at 4 weeks after surgery, and before receiving postoperative carelizumab treatment only for test groups A and B, then every 12 weeks (±14 days), and every 24 weeks (±28 days) starting from the third year, until radiological progression/recurrence, withdrawal of informed consent, loss to follow-up, or death, whichever occurs first.
- At the time of the first radiographic disease progression while receiving camrelizumab, if the subject's clinical status is stable as assessed by the investigator and the subject's informed consent is obtained, it is recommended to repeat radiological examination after an interval of 4-6 weeks for confirmation. If disease progression is not confirmed, tumor assessments will be performed at the original planned frequency. If it is confirmed, and the investigator judges that the subject continues to benefit, tumor assessments will continue at the frequency specified in the original protocol during subsequent treatment. If the subject's clinical symptoms are unstable, there is no need for further radiological confirmation.
- When a subject discontinus study treatment for reasons other than radiological disease progression, an radiological examination should be performed at the end of treatment (unless it has been performed within 28 days), and thereafter, whenever possible, at the same frequency until radiological progression/ recurrence, withdrawal of informed consent, loss to follow-up, or death.

Surgery assessment:

Surgery for esophageal cancer will be evaluated for the extent of radical resection, surgical complications, perioperative mortality (within 30 days post-surgery, within 90 days post-surgery), length of hospital stay, and rate of reoperation, among others;

	**Pathological evaluation:**<br>Tumor regression grade (TRG) will be assessed based on the Mandard criteria, and yp staging is performed according to the 8th edition of AJCC.<br><br>**Follow-up:**<br>After the end of treatment, the subjects enter the follow-up period, which is divided into safety follow-up and survival follow-up. Subjects who discontinue treatment for reasons other than disease progression/recurrence should also be followed for tumor progression/recurrence.<br><br>**Safety follow-up:**<br>The end of the safety follow-up period is defined as 90 days after the last dose of camrelizumab for the test group, or 30 days after surgery for patients who have undergone surgery and 30 days after the last dose of study drug for patients who have not undergone surgery in the control group, or screening failure. Only SAEs considered related to the study drug are collected after the safety follow-up period. Subjects in the test group are followed up every 30 days starting from the last dose of camrelizumab until 90 days, through clinical visits or telephone interviews, to collect information on the subjects' survival status, AEs, concomitant medications, and concomitant treatments. The investigator may increase visits as needed for AE follow-up, with the aim of monitoring the resolution of AEs.<br><br>**Tumor progression/recurrence follow-up:**<br>For subjects who discontinue treatment for reasons other than disease progression/ recurrence, radiological examinations should continue at the frequency specified in the protocol to follow up on the time of disease progression/recurrence, until radiological progression/recurrence, withdrawal of informed consent, loss to follow-up, or death.<br>**Survival follow-up:**<br>After the end of study treatment, survival follow-up begins, occurring every 3 months, through clinical visits or telephone interviews, to collect survival information and information on post-study anti-tumor therapy and time of disease progression, until the subject's death, loss to follow-up, or study termination.
**Study Drugs**	• Camrelizumab for injection, 200 mg/vial, referred to as “camrelizumab”;<br>• Paclitaxel for injection (albumin bound), 100 mg/vial, referred to as “albumin-bound paclitaxel”<br>• Paclitaxel injection, 5 mL:30 mg, referred to as “paclitaxel”<br>• Cisplatin injection, 6 mL:30mg, referred to as “cisplatin”
**Administration Regimen**	**Test group (A):**<br>Pre-surgery: every 3 weeks as a treatment cycle (Q3W) for a total of 2 cycles<br>Camrelizumab 200 mg/dose on D1, IV drip, Q3W;<br>Albumin-bound paclitaxel 125 mg/m^2 on D1/D8, IV drip, Q3W;<br>Cisplatin 75 mg/m^2 on D1, IV drip, Q3W;<br>The drugs are infused in the following order:<br>Camrelizumab → Albumin-bound paclitaxel → Cisplatin, with at least a 30 min interval;<br>Post-surgery: every 3 weeks as a treatment cycle (Q3W)<br>Camrelizumab 200 mg/dose on D1, IV drip, Q3W, until radiological disease recurrence/progression, intolerable toxicity, initiation of other systemic anti-tumor therapy, withdrawal of informed consent, or discontinuation of study treatment as judged by the investigator. Throughout the study, the maximum duration of camrelizumab treatment is up to a total of 17 doses before and after surgery.

Test group (B):

Pre-surgery: every 3 weeks as a treatment cycle (Q3W) for a total of 2 cycles

Camrelizumab 200 mg/dose on D1, IV drip, Q3W;
Paclitaxel 175 mg/m^2 on D1, IV drip , Q3W;
Cisplatin 75 mg/m^2 on D1, IV drip, Q3W;
The drugs are infused in the following order:
Camrelizumab → Paclitaxel → Cisplatin, with at least a 30 min interval;

Post-surgery: every 3 weeks as a treatment cycle (Q3W)

Camrelizumab 200 mg/ dose on D1, IV drip, Q3W, until radiological disease recurrence/progression, intolerable toxicity, initiation of other systemic anti-tumor trerapy, withdrawal of informed consent, or discontinuation of study treatment as judged by the investigator. Throughout the study, the maximum duration of camrelizumab treatment is up to a total of 17 doses before and after surgery.

Control group (C):

Pre-surgery: every 3 weeks as a treatment cycle (Q3W) for a total of 2 cycles

Paclitaxel 175 mg/m^2 on D1, IV drip, Q3W;
Cisplatin 75 mg/m^2 on D1, IV drip, Q3W;

Post-surgery: follow-up observation;

Inclusion Criteria

Subjects must meet all of the following criteria to be enrolled in this study:

1. Having signed a written ICF, and voluntarily participating in this study;
2. Histopathologically or cytologically confirmed esophageal squamous cell carcinoma;
3. Thoracic esophageal cancer assessed by CT/MRI/EUS etc., with the clinical staged of T1b-3N1-3M0 or T3N0M0 (according to the 8th edition of AJCC);
4. Expected to achieve R0 resection;
5. Aged 18-75 years, male or female;
6. ECOG PS 0-1;
7. Having not received any prior anti-tumor therapy for esophageal cancer, including radiotherapy, chemotherapy, surgery, etc.;
8. Planned surgery after completion of neoadjuvant therapy;
9. No contraindication to surgery;
10. Normal major organ functions, including:
    a) Hematology (no use of any blood components, cell growth factors, leukocyte-elevating drugs, platelet-elevating drugs, or anaemia-correcting drugs within 14 days before the first use of study drug):
        - Neutrophil count ≥ 1.5×10^9/L
        - Platelet count ≥ 100×10^9/L
        - Haemoglobin ≥ 90 g/L
    b) Blood biochemistry:
        - Total bilirubin ≤ 1.5×ULN
        - ALT ≤ 2.5×ULN, AST ≤ 2.5×ULN,
        - Serum creatinine ≤ 1.5×ULN, or creatinine clearance ≥ 50 mL/min (Cocheroft-Gault formula)
    c) Coagulation function:
        - International normalized ratio (INR) ≤ 1.5×ULN
        - Activated partial thromboplastin time (APTT) ≤ 1.5×ULN
11. Female subjects of childbearing potential should have a negative serum pregnancy test within 72 hours before taking the study drug, and use effective contraception (e.g., intrauterine devices, contraceptives, or condoms) during the study and for at least 3 months after the last dose; male subjects with female partners of childbearing potential should be surgically sterile or agree to use effective contraception during the study and for 3 months after the last dose;
12. Subjects with good compliance, and cooperating with follow-up;

**Exclusion Criteria**	**Subjects with any of the following conditions will not be enrolled in this study:**<br>1. Tumor with obvious invasion into adjacent organs of the esophageal lesion (major arteries or trachea);<br>2. Presence of metastases to supraclavicular lymph nodes;<br>3. Presence of uncontrollable pleural effusion, pericardial effusion, or ascites requiring repeated drainage;<br>4. Poor nutritional status, BMI < 18.5 kg/m^2; if corrected after symptomatic nutritional support before randomization, these patients may be considered for enrollment after evaluation by the principal investigator;<br>5. History of allergy to monoclonal antibodies, any component of camrelizumab, paclitaxel, cisplatin, or other platinum-based drugs;<br>6. Prior or ongoing treatment with any of the following:<br>a) Any radiotherapy, chemotherapy, or other anti-tumor drugs;<br>b) Use of immunosuppressive drugs, or systemic corticosteroid therapy for immunosuppressive purposes (doses > 10 mg/day prednisone or equivalent) within 2 weeks prior to the first dose of study drug; inhaled or topical steroids and adrenal corticosteroid replacement at doses > 10 mg/day prednisone or equivalent are permitted in the absence of active autoimmune disease;<br>c) Having received a live attenuated vaccine within 4 weeks prior to the first use of study drug;<br>d) Major surgery or serious trauma within 4 weeks prior to the first use of study drug;<br>7. History of any active autoimmune disease or autoimmune disease, including but not limited to: interstitial pneumonia, enteritis, hepatitis, hypophysitis, vasculitis, nephritis, hyperthyroidism, hypothyroidism (considered after hormone replacement therapy); patients with psoriasis or childhood asthma/allergy that have resolved without any intervention in adulthood may be considered for inclusion, but patients requiring medical intervention with bronchodilators should not be included;<br>8. History of immunodeficiency, including HIV test positive, or presence of other acquired or congenital immune deficiencies, or history of organ transplantation or allogeneic bone marrow transplantation;<br>9. Subject with clinically uncontrolled cardiac symptoms or diseases, including but not limited to: (1) heart failure of NYHA class II or above, (2) unstable angina pectoris, (3) myocardial infarction within 1 year, (4) clinically significant supraventricular or ventricular arrhythmias without clinical intervention or uncontrolled with clinical intervention;<br>10. Serious infection (CTCAE > Grade 2) within 4 weeks prior to the first dose of study drug, such as severe pneumonia, bacteraemia, and infection complications requiring hospitalization; active pulmonary inflammation based on chest X-ray at baseline; symptoms and signs of infection requiring oral or intravenous antibiotic therapy within 14 days prior to the first dose of study drug, except prophylactic use of antibiotics;<br>11. Patients with active tuberculosis infection based on medical history or CT test, or patients with a history of active tuberculosis infection within 1 year before enrollment, or patients with active tuberculosis infection beyond 1 year but without proper treatment;<br>12. Presence of active hepatitis B (HBV DNA ≥ 2000 IU/mL or 10^4 copies/mL) or hepatitis C (positive for hepatitis C antibody and HCV RNA above the lower limit of detection of the assay);<br>13. Other malignancies diagnosed within 5 years prior to the first dose of study drug. Except malignancies with low risk of metastasis or death (5-year survival rate > 90%), such as adequately treated basal cell carcinoma of skin or squamous cell skin cancer or carcinoma in situ of the cervix;<br>14. Pregnant or lactating women;

	15. Presence of other factors that may lead to forced withdrawal from the study as judged by the investigator, such as other serious diseases (including psychiatric disorders) requiring concomitant therapy, alcoholism, drug abuse, family or social factors, which may affect the safety or compliance of the subjects.
**Discontinuation Criteria**	Discontinuation of study treatment does not represent withdrawal from the study. Subjects who discontinue study treatment, as long as they have not withdrawn informed consent to refuse further follow-up, are required to complete the remaining study visits as required by the protocol.<br>A subject must discontinue study treatment if any of the following criteria is met:<br>1. Having completed all protocol-specified treatments;<br>2. Withdrawal of informed consent, refusing to continue receiving study drug treatment;<br>3. Worsening of clinical symptoms or decline of physical condition as judged by the investigator;<br>4. Radiological disease progression/tumor recurrence or metastasis, unless subjects in the test group meet the criteria for continued treatment after disease progression and agree to continue treatment;<br>5. Occurrence of intolerable AEs, or other events affecting the safety of the subject, including but not limited to any clinical AEs, laboratory abnormalities, or other medical conditions;<br>6. Significant protocol deviations;<br>7. Other situations deemed necessary by the investigator to discontinue study drug treatment;<br>8. Subject request;<br>9. Pregnancy;<br>10. Loss to follow-up;<br>11. Initiation of other systemic anti-tumor therapy.
**Study Withdrawal Criteria**	Reasons for subject's withdrawal from the study may include:<br>• Subject's withdrawal of informed consent, refusing further follow-up;<br>• Loss to follow-up;<br>• Death;<br>• Termination of the study;<br>• Other situations deemed necessary by the investigator for withdrawal from the study.
**Study Early Termination Criteria**	Reasons for early termination or suspension of the study may include:<br>• Identified unexpected, significant, or unacceptable risks to the subjects.<br>• Existing efficacy results, which support early termination of the study.<br>• Significant errors found in the protocol during the implementation of the study.<br>• Great difficulty in completing the study, and low compliance with protocol requirements due to reasons such as severe lagging in subject enrollment or frequent protocol deviations.<br>• Ineffectiveness of the study drug/study treatment, making it meaningless to continue the study;<br>• Incomplete or inaccurate data.
**Tissue Samples**	Subjects should agree to provide formalin-fixed paraffin-embedded (FFPE) tumor tissue blocks or at least 10 slides of unstained tumor specimens obtained before randomization, and about 10 mL of whole blood samples should be collected at baseline;
**Determination of Sample Size**	The study's co-primary endpoints are pCR and EFS, and the final analysis of the study will be performed when approximately 228 EFS events are observed, with a planned sample size of about 390 subjects.<br><br>(1) pCR: Assuming that the pCR of test group A is 30%, the pCR of test group B is 25%, and the pCR of control group C is 9%, with a randomization ratio of

	1:1:1, and α set at one-sided 0.005, 111 subjects in each group are expected to provide at least 93% power to detect that test group A is superior to control group C, and can provide at least 75% power to detect that test group B is superior to control group C. Considering a 15% dropout rate, each group will need 130 subjects.<br><br>(2) EFS: Assuming the median EFS in the control group is 30 months, the hazard ratio (HR) for the test group (A+B) compared to the control group is 0.67, with an initial α assignment set at one-sided 0.02, the efficacy interim analysis of EFS is planned when 70% of events is observed;<br>- If the randomization ratio is 2:1 (test group A+B vs. control group C), then 228 events (141 in the test group, 87 in the control group) are required to provide at least 80% power to detect the superiority of the test group over the control group, with an enrollment period of 36 months, a total study duration of 84 months, and a dropout rate of 15%, so approximately 390 subjects are needed for the three groups.<br>- If the randomization ratio is 1:1 (test group A vs. control group C or test group B vs. control group C), 158 events (71 in the test group, 87 in the control group) can be observed in the above sample size, to provide at least 67% power to detect the superiority of test group B or test group A over the control group.
**Statistical Methods**	**Statistical Hypotheses**<br>This study is designed for superiority, and is statistically tested for the dual primary endpoints pCR and EFS using the multiplicity test. The multiplicity strategy is detailed in Section 11.4.5. This study includes two primary hypotheses and three secondary hypotheses.<br>The two primary hypotheses are:<br>1) pCR: Test group A vs. control group<br>H_{01} (null hypothesis): The pCR rate of test group A is lower than or equal to that of the control group.<br>H_{11} (alternative hypothesis): The pCR rate of test group A is higher than that of the control group.<br>2) EFS: Test group A + B vs. control group<br>H_{02} (null hypothesis): The EFS of test group A+B is worse than or equal to that of the control group.<br>H_{12} (alternative hypothesis): The EFS of test group A+B is superior to that of the control group.<br>The three secondary hypotheses are:<br>3) pCR: Test group B vs. control group<br>H_{03} (null hypothesis): The pCR rate of test group B is lower than or equal to that of the control group.<br>H_{13} (alternative hypothesis): The pCR rate of test group B is higher than that of the control group.<br>4) EFS: Test group A vs. control group<br>H_{04} (null hypothesis): The EFS of test group A is worse than or equal to that of the control group.<br>H_{14} (alternative hypothesis): The EFS of test group A is superior to that of the control group.<br>5) EFS: Test group B vs. control group<br>H_{05} (null hypothesis): The EFS of test group B is worse than or equal to that of the control group.<br>H_{15} (alternative hypothesis): The EFS of test group B is superior to that of the control group.<br><br>The Cochran-Mantel-Haenszel (CMH) chi-square test is used for intergroup comparison of pCR, and the stratified Logrank test is used for intergroup comparison of EFS. Stratification factors include clinical stage (Stage I/II vs. Stage III vs. Stage IVa). The sequence of tests is detailed in Section 11.4.5 on

multiplicity, and the specific process of hypothesis testing is detailed in the statistical analysis plan (SAP).

General Analysis

For continuous data, statistics such as number, mean, standard deviation, median, minimum, and maximum values will be summarized; for categorical data, statistics such as frequency and percentage will be summarized; for time-to-event data, the Kaplan-Meier method will be used to estimate the survival function and the median time to event occurrence, and survival curves will be plotted.

Efficacy Analysis

The primary efficacy endpoints of this study are pCR and EFS.

pCR will be compared using the CMH test based on stratification factors, with differences in pCR comparisons between test group A and control group, test group B and control group, along with 95% confidence intervals (CIs) and p-values listed separately.

EFS will be compared using the log-rank method considering stratification factors for the predefined intergroup differences in EFS. The Kaplan-Meier method will be used to estimate the median EFS for test group A+B, test group A, test group B, and control group C, and the Brookmeyer-Crowley method will be used to estimate the two-sided 95% CIs for the median EFS, with Kaplan-Meier curves for EFS of test group A+B, test group A, test group B, and control group C plotted. HRs and 95% CIs are estimated for test group A + B, test group A, and test group B relative to control group C based on a stratified Cox proportional model considering stratification factors.

For the time-to-event data among secondary endpoints, DFS and OS are analyzed using the same method as EFS, but the intergroup survival differences are only subjected to informal statistical hypothesis testing, and nominal p-values can be reported if needed; for the grade data among secondary endpoints, such as post-surgery pathological stage (ypTNM stage), the distribution of stage before and after medication will be described; for the binary data among secondary endpoints, the R0 resection rate, the MPR, etc. are analyzed using the same method as the primary efficacy endpoint pCR, but the inter-group differences are only subjected to informal statistical hypothesis testing, and nominal p-values can be reported if needed.

Safety Analysis

Safety analysis is primarily based on descriptive statistical analysis. All AEs will be coded using MedDRA and graded according to the NCI-CTCAE version 5.0 grading system; AEs assessed as surgical complications will also be graded according to the CD criteria. The worst grade of all AEs, drug-related AEs, SAEs, and drug-related SAEs occurring during treatment will be listed.

Laboratory test results, vital signs, ECG data, etc. will be analyzed using conversion tables to compare the baseline and post-baseline conditions.

Interim Analysis

An interim efficacy analysis of EFS is planned for this study when 70% of EFS events (160 cases) have been collected (test group A+B vs. control group). [redacted]

	[redacted]<br><br>The final analysis of pCR and the interim analysis of EFS will be performed by an independent statistical team, and their results will be evaluated by an independent data monitoring committee (iDMC). For a more detailed plan of the final analysis of pCR and the interim analysis of EFS, please refer to the SAP and the iDMC charter.<br><br>**Multiplicity**<br>The overall type I error (α) for the two primary hypotheses H_{01} and H_{02} is controlled at 2.5% (one-sided), with 0.5% assigned to the pCR hypothesis for test group A vs. control group, and 2% assigned to the EFS hypothesis for test group A+B vs. control group. α is assigned and transferred across multiple hypotheses using the graphical method of Maurer and Bretz[17] (see Section 11.4.5 for details).
**Study Duration**	Estimated first subject enrollment date: December 2020<br>Estimated last subject enrollment date: December 2023<br>Estimated study completion date: December 2027

Schedule of Activities

<table>
<tr><th></th><th colspan="3">Screening Period [1]</th><th colspan="4">Preoperative Neoadjuvant</th><th>Preoperative [3]</th><th>Surgery</th><th>Postoperative Adjuvant</th><th colspan="3">Follow-up Period</th></tr>
<tr><th></th><th>Within 3 weeks prior to randomization</th><th>Within 2 weeks prior to randomization</th><th>Within 1 week prior to randomization</th><th>C1D1[1]</th><th>C1D8[2] ±3d</th><th>C2 D1 ±3d</th><th>C2 D8 ±3d</th><th></th><th></th><th>CXD1 ±3d</th><th>End of Treatment [4] ±7d</th><th>Safety Follow-up Within 90 ± 7 days</th><th>Tumor Progression/ Recurrence and Survival Follow-up</th></tr>
<tr><td colspan="14">Baseline Data</td></tr>
<tr><td>Signing of ICF</td><td>×</td><td></td><td></td><td></td><td></td><td></td><td></td><td></td><td></td><td></td><td></td><td></td><td></td></tr>
<tr><td>Demographics [5]</td><td>×</td><td></td><td></td><td></td><td></td><td></td><td></td><td></td><td></td><td></td><td></td><td></td><td></td></tr>
<tr><td>Tumor History and Treatment History [6]</td><td>×</td><td></td><td></td><td></td><td></td><td></td><td></td><td></td><td></td><td></td><td></td><td></td><td></td></tr>
<tr><td>Other Medical History[7]</td><td>×</td><td></td><td></td><td></td><td></td><td></td><td></td><td></td><td></td><td></td><td></td><td></td><td></td></tr>
<tr><td>Concomitant Medications and Concomitant Therapies [8]</td><td>×</td><td>×</td><td>×</td><td colspan="9">×</td><td></td></tr>
<tr><td colspan="14">Laboratory Tests</td></tr>
<tr><td>Hematology [9]</td><td></td><td></td><td>×</td><td>×</td><td>×</td><td>×</td><td>×</td><td>×</td><td></td><td>×</td><td>×</td><td></td><td></td></tr>
<tr><td>Blood Chemistry [10]</td><td></td><td>×</td><td></td><td>×</td><td>×</td><td>×</td><td>×</td><td>×</td><td></td><td>×</td><td>×</td><td></td><td></td></tr>
<tr><td>Thyroid Function [11]</td><td></td><td>×</td><td></td><td></td><td></td><td>×</td><td></td><td>×</td><td></td><td>×</td><td>×</td><td></td><td></td></tr>
<tr><td>Cardiac Enzyme Spectrum [12]</td><td></td><td>×</td><td></td><td></td><td></td><td>×</td><td></td><td>×</td><td></td><td>×</td><td>×</td><td></td><td></td></tr>
<tr><td>Virology [13]</td><td>×</td><td></td><td></td><td></td><td></td><td></td><td></td><td></td><td></td><td></td><td></td><td></td><td></td></tr>
<tr><td>Urinalysis [14]</td><td></td><td>×</td><td></td><td></td><td></td><td>×</td><td></td><td>×</td><td></td><td>×</td><td>×</td><td></td><td></td></tr>
<tr><td>Fecal Occult Blood [15]</td><td></td><td>×</td><td></td><td></td><td colspan="9">Perform as needed</td></tr>
</table>

	**Screening Period [1]**			**Preoperative Neoadjuvant**				**Preoperative [3]**	**Surgery**	**Postoperative Adjuvant**	**Follow-up Period**		
	**Within 3 weeks prior to randomization**	**Within 2 weeks prior to randomization**	**Within 1 week prior to randomization**	**C1D1[1]**	**C1D8[2] ±3d**	**C2D1 ±3d**	**C2D8 ±3d**			**CXD1 ±3d**	**End of Treatment [4] ±7d**	**Safety Follow-up Within 90 ± 7 days**	**Tumor Progression/ Recurrence and Survival Follow-up**
Coagulation Function [16]		×				×		×		×	×		
Pregnancy Test [17]			×										
Clinical Assessment, Examinations													
AEs [18]	×	×	×	×	×	×	×	×	×	×	×	×	
Surgery Records and Perioperative Complications [19]									×				
Vital Signs [20]			×	×	×	×	×	×		×	×		
Physical Examination [21]		×		×	×	×	×	×		×	×		
Height [22]		×											
Weight [23]			×	×	×	×	×	×		×	×		
ECOG PS[24]			×	×		×		×		×	×		
12-lead ECG[25]		×				×		×		×	×		
Echocardiogram [26]		×			Perform as needed								
Study Drugs													
Camrelizumab Administration [27]				×		×				×			
Albumin-bound Paclitaxel Administration [28]				×	×	×	×						

	**Screening Period [1]**			**Preoperative Neoadjuvant**				**Preoperative [3]**	**Surgery**	**Postoperative Adjuvant**	**Follow-up Period**		
	**Within 3 weeks prior to randomization**	**Within 2 weeks prior to randomization**	**Within 1 week prior to randomization**	**C1D1[1]**	**C1D8[2] ±3d**	**C2D1 ±3d**	**C2D8 ±3d**			**CXD1 ±3d**	**End of Treatment [4] ±7d**	**Safety Follow-up Within 90 ± 7 days**	**Tumor Progression/ Recurrence and Survival Follow-up**
Paclitaxel Administration [29]				×		×							
Cisplatin Administration [30]				×		×							
Efficacy Assessment													
Radiological Assessment [31]	×							×		×	×	×	×
Pathological Evaluation [32]									×				
Tissue and Blood Samples [33]		×											
Follow-up after End of Treatment													
Disease Progression /Time to Recurrence													×
Subsequent Anti-tumor Therapy [34]													×
Death Time													×
Collection and Assessment of Patient-reported Outcomes (PROs)													
Collection and Assessment of Patient-reported Outcomes			×					×		×	×	×	

	Screening Period [1]			Preoperative Neoadjuvant				Preoperative [3]	Surgery	Postoperative Adjuvant	Follow-up Period		
	Within 3 weeks prior to randomization	Within 2 weeks prior to randomization	Within 1 week prior to randomization	C1D1[1]	C1D8[2] ±3d	C2D1 ±3d	C2D8 ±3d			CXD1 ±3d	End of Treatment [4] ±7d	Safety Follow-up Within 90 ± 7 days	Tumor Progression/ Recurrence and Survival Follow-up
(PROs) [35]													

Notes:

1. If the tests or examinations completed by the subject at the study site before signing the ICF (such as radiological assessment, etc.) are within the time limits specified by the study, there is no need to repeat them during the screening period; laboratory tests and physical examinations prior to each dose should be completed within 3 days before dosing; if the examinations and tests completed during the screening period fall within the window before the first dose, there is no need to repeat them before the first dose.
2. C1D8: Administration and various tests and examinations are only for test group A;
3. Preoperative visits and related assessments should be conducted within 14 days before surgery, and in accordance with the treatment principles of each site. If surgery treatment exceeds 6 weeks after the last dose of neoadjuvant treatment, relevant assessments will be repeated within 14 days before surgery.
4. End of treatment visit: When the subject meets any reason for the end of study treatment, the relevant tests and examinations for end of treatment visit are required. If the specified tests and examinations for end of treatment have been completed within 14 days before withdrawal from treatment, there is no need to repeat them.
5. **Demographics:** It includes date of birth, age, gender, ethnicity, place of origin;
6. **Tumor History and Treatment History:** It includes tumor diagnosis, surgical history, local treatment history, systemic treatment history, and radiotherapy history. Tumor diagnosis should include at least histological classification, histological grade, clinical stage, and time of initial diagnosis;
7. **Other Medical History:** It includes drug allergy history, diagnosis and treatment history of other diseases, tumor history other than esophageal cancer;
8. **Concomitant Medications and Concomitant Therapies:** All concomitant medications and concomitant therapies will be collected from signing the ICF until the end of the safety follow-up period (90 days after the last dose of camrelizumab for the test group, 30 days after surgery for patients who have undergone surgery and 30 days after the last dose of study drug for patients who have not undergone surgery in the control group, or screening failure), and concomitant medications and concomitant therapies related to surgical complications up to 90 days after surgery should be collected for subjects who undergo surgery; if subjects start other new anti-tumor therapies before the end of the safety follow-up period, only concomitant medications and concomitant therapies for AEs/SAEs suspected to be related to the study drug or fatal will be collected after new anti-tumor therapies; concomitant medications and concomitant therapies for SAEs related to the study drug will be recorded after the safety follow-up period;.
9. **Hematology:** It includes total blood cell differential count + five-part differential, red blood cell count, haemoglobin, platelet count; it will be performed at screening, before dosing on CXD1 and CXD8 in the preoperative neoadjuvant stage, and before surgery; in the postoperative adjuvant stage, only the test group undergoes the test before each dose of camrelizumab on CXD1, and at the end of camrelizumab treatment; in the postoperative adjuvant stage, the control group undergoes the test as clinically indicated, on an as-needed basis;
10. **Blood Chemistry:** It includes total bilirubin (TBIL), direct bilirubin (DBIL), alanine aminotransferase (ALT), aspartate aminotransferase (AST), alkaline phosphatase, total protein, albumin, blood urea nitrogen (BUN), creatinine (Cr), endogenous creatinine clearance (Ccr), blood glucose, amylase, potassium (K), sodium (Na), chlorine (Cl), calcium (Ca), phosphorus (P); it will be performed at screening, before dosing on CXD1 and CXD8 in the preoperative neoadjuvant

stage, and before surgery; in the postoperative adjuvant stage, only the test group undergoes the test before each dose of camrelizumab on CXD1, and at the end of camrelizumab treatment; in the postoperative adjuvant stage, the control group undergoes the test as clinically indicated, on an as-needed basis;

11. **Thyroid Function:** It includes serum free triiodothyronine (FT3), free thyroxine (FT4), and serum thyroid-stimulating hormone (TSH), with triiodothyronine (T3) and thyroxine (T4) as alternatives; it will be performed at screening, before dosing on C2D1 in the preoperative neoadjuvant stage, and before surgery; in the postoperative adjuvant stage, only the test group undergoes the test before each dose of camrelizumab on CXD1, and at the end of camrelizumab treatment; in the postoperative adjuvant stage, the control group undergoes the test as clinically indicated, on an as-needed basis; if the test time for FT3 and FT4 is long, an assessment based on T3, T4, and TSH should be conducted before dosing to evaluate if treatment can continue;
12. **Cardiac Enzyme Spectrum:** It includes creatine kinase-MB isoenzyme (CK-MB), cardiac troponin I (cTnI), lactate dehydrogenase (LDH); it will be performed at screening, before dosing on C2D1 in the preoperative neoadjuvant stage, and before surgery; in the postoperative adjuvant stage, only the test group undergoes the test before each dose of camrelizumab on CXD1, and at the end of camrelizumab treatment; in the postoperative adjuvant stage, the control group undergoes the test as clinically indicated, on an as-needed basis;
13. **Virology:** It includes HBsAg (if positive, HBV-DNA is required), HBsAb, HBeAg, HBeAb, HBcAb, HCV-Ab (if positive, HCV-RNA is required), HIV-Ab; it will be performed at screening only; virology results obtained before signing the ICF that meet the requirements of this protocol can be used for screening assessment;
14. **Urinalysis:** It includes urine protein, occult blood, red blood cells, white blood cells; it will be performed at screening, before dosing on C2D1 in the preoperative neoadjuvant stage, and before surgery; in the postoperative adjuvant stage, only the test group undergoes the test before each dose of camrelizumab on CXD1, and at the end of camrelizumab treatment; in the postoperative adjuvant stage, the control group undergoes the test as clinically indicated, on an as-needed basis;
15. **Fecal Occult Blood:** It is only performed at screening, and thereafter as clinically indicated, on an as-needed basis;
16. **Coagulation Function:** It includes international normalized ratio (INR), activated partial thromboplastin time (APTT), prothrombin time (PT), fibrinogen (FIB); it will be performed at screening, before dosing on C2D1 in the preoperative neoadjuvant stage, and before surgery; in the postoperative adjuvant stage, only the test group undergoes the test before each dose of camrelizumab on CXD1, and at the end of camrelizumab treatment; in the postoperative adjuvant stage, the control group undergoes the test as clinically indicated, on an as-needed basis;
17. **Pregnancy Test:** At screening, female subjects of childbearing potential should have a negative serum pregnancy test within 72 hours prior to the first dose; female subjects of childbearing potential and male subjects with partners of childbearing potential should take effective contraception methods (such as intrauterine devices, contraceptives, or condoms) during the study or for at least 3 months after the last dose;
18. **AEs:** AEs will be recorded from signing of ICF until the end of the safety follow-up period (90 days after the last dose of camrelizumab for the test group, 30 days after surgery for patients who have undergone surgery or 30 days after the last dose of study drug for patients who have not undergone surgery in the control group, or screening failure); if the subject starts other new anti-tumor therapy before the end of the safety follow-up period, for non-fatal AEs/SAEs suspected to be unrelated to the study drug, the collection period ends when the other new anti-tumor therapies begin. Deaths occurring during the safety follow-up period must be reported as SAEs, regardless of whether or not there are other treatments. After the end of the safety follow-up period, only SAEs related to the study drug should be reported; surgery-related complications occurring within 30 days and 90 days after surgery should be collected for subjects undergoing surgery;
19. **Surgery Records and Perioperative Complications:** The surgery process will be recorded by video, to document the surgery duration, surgical approach, number of lymph nodes dissected, and the status of surgical resection (R0/R1/R2). The mortality, complications caused by surgery, length of hospital stay and reoperation rate at 30 days and 90 days after surgery (perioperative period) will be recorded;
20. **Vital Signs:** It includes body temperature, pulse, respiratory rate, blood pressure; it will be performed at screening, before dosing on CXD1 and CXD8 in the preoperative neoadjuvant stage, and before surgery; in the postoperative adjuvant stage, only the test group undergoes the examination before each dose of camrelizumab on CXD1, and at the end of camrelizumab treatment; in the postoperative adjuvant stage, the control group undergoes the examination as clinically indicated, on an as-needed basis;

21. **Physical Examination:** It includes general condition, head and face, eyes, nose and throat, oral cavity, skin, lymph nodes, respiratory system, cardiovascular system, gastrointestinal system, genitourinary system, musculoskeletal system, nervous system, mental status, and others; it will be performed at screening, before dosing on CXD1 and CXD8 in the preoperative neoadjuvant stage, and before surgery; in the postoperative adjuvant stage, only the test group undergoes the examination before each dose of camrelizumab on CXD1, and at the end of camrelizumab treatment; in the postoperative adjuvant stage, the control group undergoes the examination as clinically indicated, on an as-needed basis;
22. **Height:** It will be performed at **s**creening only;
23. **Weight:** It will be performed at screening, before dosing on CXD1 and CXD8 in the preoperative neoadjuvant stage, and before surgery. In the postoperative adjuvant stage, only the test group undergoes the examination before each dose of camrelizumab on CXD1, and at the end of camrelizumab treatment; in the postoperative adjuvant stage, the control group undergoes the examination as clinically indicated, on an as-needed basis;
24. **ECOG PS**: It will be performed at screening, before dosing on CXD1 in the preoperative neoadjuvant stage, and before surgery. In the postoperative adjuvant stage, only the test group undergoes the examination before each dose of camrelizumab on CXD1, and at the end of camrelizumab treatment; in the postoperative adjuvant stage, the control group undergoes the examination as clinically indicated, on an as-needed basis;
25. **12-lead ECG**: The heart rate, QT interval, and P-R interval should be indicated; it will be performed at screening, before doing on C2D1 in the preoperative neoadjuvant stage, and before surgery; in the postoperative adjuvant stage, only the test group undergoes the examination before each dose of camrelizumab on CXD1, and at the end of camrelizumab treatment; in the postoperative adjuvant stage, the control group undergoes the examination as clinically indicated, on an as-needed basis;
26. **Echocardiogram:** Left ventricular ejection fraction (LVEF, %) should be included; it should be performed at screening only, and as needed as clinically indicated after randomization;
27. **Camrelizumab Administration:** Test groups A and B received camrelizumab 200 mg/dose on Day 1, by IV drip for approximately 30-60 minutes, Q3W, 2 treatment cycles in the preoperative neoadjuvant stage, and continued in the postoperative adjuvant stage until radiological disease recurrence/progression, toxicity intolerance, initiation of other systemic anti-tumor therapy, withdrawal of consent, or discontinuation of study treatment as judged by the investigator. Throughout the study, the maximum duration of camrelizumab administration should be a total of 17 doses before and after surgery;
28. **Albumin-bound Paclitaxel Administration:** For test group A, 125 mg/m^2 on Day 1 and Day 8, IV drip, Q3W, 2 treatment cycles in the preoperative neoadjuvant stage;
29. **Paclitaxel Administration:** For test group B and control group C, 175 mg/m^2 on D1, IV drip, Q3W, 2 treatment cycles in the preoperative neoadjuvant stage;
30. **Cisplatin Administration:** 75 mg/m^2 on D1, IV drip, Q3W, 2 treatment cycles in the preoperative neoadjuvant stage;
31. **Radiological Assessment:** Radiographic response assessment should be performed regularly according to RECIST 1.1 criteria, including enhanced CT scan of neck/chest/abdomen (CT scan slice thickness ≤ 5 mm) or enhanced MRI scan; neck ultrasound is recommended to be added for the diagnosis and differential diagnosis of metastatic lesions such as cervical lymph nodes; enhanced MRI or enhanced CT of the brain is also required to exclude brain metastases if brain metastases are suspected; when metastases to bone are confirmed or clinically suspected, a bone scan is required; unless specifically stated otherwise, the permissible window period for radiological examination is ±7 days. When disease progression is suspected (such as worsening of symptoms), unscheduled radiological examinations may be conducted.
    a) At screening, all subjects should undergo tumor radiological assessments; the tumor baseline assessments can be relaxed to within 3 weeks before randomization, and CT/MRI results obtained before signing the ICF can be used for the tumor assessment at screening if they meet the requirements of this protocol, **and EUS should be added when making clinical staging judgments**; After randomization, the conditions for radiological examination should be the same as the baseline (including scan slice thickness, contrast agents, etc.). Tumor radiological assessments should be performed within 14 days before surgery (i.e., within 3-4 weeks after the last dose of neoadjuvant therapy, or within 14 days before surgery if surgery exceeds 6 weeks after the last dose of neoadjuvant therapy), at 4 weeks after surgery, and before receiving postoperative carelizumab treatment only for test groups A and B, then every 12 weeks

(±14 days), and every 24 weeks (±28 days) starting from the third year, until radiological progression/ recurrence, withdrawal of informed consent, loss to follow-up, or death, whichever occurs first;

b) At the time of the first radiographic disease progression while receiving camrelizumab, if the subject's clinical status is stable as assessed by the investigator and the subject's informed consent is obtained, it is recommended to repeat radiological examination after an interval of 4-6 weeks for confirmation. If disease progression is not confirmed, tumor assessments will be performed at the original planned frequency. If it is confirmed, and the investigator judges that the subject continues to benefit, tumor assessments will continue at the frequency specified in the original protocol during subsequent treatment. If the subject's clinical symptoms are unstable, there is no need for further radiological confirmation;

c) When a subject discontinus study treatment for reasons other than radiological disease progression, an radiological examination should be performed at the end of treatment (unless it has been performed within 28 days), and thereafter, whenever possible, at the same frequency until radiological progression/ recurrence, withdrawal of informed consent, loss to follow-up, or death;

d) The time of radiological examination should follow calendar days and should not be adjusted for the delay in medication cycle;

32. **Pathological Assessment:** The surgical specimens should be preserved and processed according to the Pathological Sample Operating Manual, tumor regression grade should be assessed according to the Mandard criteria, and the degree of radical resection and yp stage should be assessed according to the 8th edition of AJCC;
33. **Tissue and Blood Samples:** Before randomization, subjects should agree to provide formalin-fixed paraffin-embedded (FFPE) tumor tissue blocks or at least 10 slides of unstained tumor specimens obtained prior to study treatment, and approximately 10 mL of whole blood samples will be collected at baseline. In principle, tissue samples within 21 days prior to randomization and whole blood samples within 7 days prior to randomization are required;
34. **Subsequent Anti-tumor Therapy：** After discontinuation of study treatment, survival status and subsequent anti-tumor information will be collected every 3 months by clinical follow-up or telephone follow-up until death, loss to follow-up, or end of study.
35. **Collection and Assessment of Patient-reported Outcomes (PROs):** EORTC QLQ-C30 (version 3) and OES18 quality of life questionnaires will be collected and assessed at screening, within 14 days before surgery, 4 weeks after surgery (before receiving postoperative camrelizumab for test groups A and B), every 12 weeks (± 14 days) thereafter, and every 24 weeks (± 28 days) from the third year, at the end of treatment/withdrawal, and at the end of the safety follow-up period.

1. Study Background and Scientific Rationale

1.1 Epidemiology and Treatment Status of Esophageal Cancer

Esophageal cancer is a malignant tumor with high incidence and mortality in the world, especially in China, where the annual number of new cases and deaths accounts for more than half of the world's total. According to China's cancer registry data in 2015[1], the incidence of esophageal cancer ranks 6th among all malignant tumors, with 246,000 new cases per year, and the mortality ranks 4th, with 188,000 deaths per year. Unlike esophageal adenocarcinoma which is more common in Europe and America, the main pathological type of esophageal cancer in China is esophageal squamous cell carcinoma, accounting for 90% of the cases, and the prognosis of esophageal squamous cell carcinoma is worse. The overall 5-year survival rate of esophageal cancer is less than 30%.

The treatment of esophageal cancer should adopt the principle of individualized comprehensive treatment, that is, according to the patient's physical condition, the pathological type of the tumor, the extent of invasion, and the trend of development, a multidisciplinary comprehensive treatment mode should be adopted, and surgery, radiotherapy, chemotherapy, molecular targeted therapy, and immunotherapy should be used in a planned and rational manner, in order to maximize the survival time of patients, improve the survival rate, control tumor progression, and improve the quality of life of patients.

In recent years, significant progress has been made in immunotherapy represented by immune checkpoint inhibitors (such as PD-1 monoclonal antibodies or PD-L1 monoclonal antibodies, etc.). Multiple randomized controlled studies have confirmed that in patients with advanced or metastatic esophageal squamous cell carcinoma who are receiving second-line treatment, therapy based on immune checkpoint inhibitors can bring survival benefits, and immunosuppressive therapy has become the standard of care for patients with advanced esophageal squamous cell carcinoma in the second-line setting[2-3].

According to the 8th edition of the AJCC/UICC, patients with T1b-cT2N+ or T3-4aNanyM0 stage can choose to undergo preoperative adjuvant chemoradiotherapy, chemotherapy or radiotherapy first, and then they can be assessed for the eligibility for surgery after completing preoperative adjuvant therapy. For patients with multi-station lymph node metastasis (N3), although it is a relative contraindication for surgery, T1-3 tumors can also be resected when there is regional lymph node metastasis (N+), and the patient's age and physical condition need to be comprehensively considered.

As a preoperative treatment regimen, neoadjuvant therapy can not only improve the local tumor control rate, increase the possibility of surgery, but also increase the R0 resection rate and bring overall survival benefits to patients. There is a growing consensus on the treatment modes of neoadjuvant chemotherapy and neoadjuvant chemoradiotherapy[4-5], but patients are prone to recurrence and distant metastasis after surgery, so more effective neoadjuvant therapies are urgently needed for patients with resectable locally advanced esophageal cancer.

In recent years, there has been continuous exploration in the postoperative adjuvant therapy for esophageal cancer. Checkmate 577 is a global, multicenter, randomized, double-blind, placebo-controlled phase 3 clinical study, which included 794 patients with esophageal cancer or gastroesophageal junction cancer who had received preoperative neoadjuvant chemoradiotherapy and did not achieve pathological complete response after surgery, and were randomized in a 2:1 ratio to the test group or control group to receive nivolumab or placebo as adjuvant therapy. The results showed that the disease-free survival (DFS) of the test group was significantly higher than that of the control group (22.4 months vs. 11.0 months, HR=0.69, P <

0.001), and post hoc subgroup analysis showed that patients with esophageal squamous cell carcinoma seemed to gain greater clinical benefit from adjuvant immunotherapy (DFS 29.7 months vs. 11.0 months, HR=0.61).

1.2 Immune Checkpoint Therapy

In the process of tumorigenesis and development, tumor cells have many genetic and epigenetic changes compared to normal cells, theoretically possessing enough antigens to be recognized by the human immune system, thereby triggering an immune response to inhibit tumor growth. However, in reality, tumors suppress the immune response against them through various pathways, thus evading the attack of the immune system.[6] For example, by highly expressing immune checkpoints PD-1 or PD-L1.

At present, several humanized monoclonal antibodies against PD-1/PD-L1 have been marketed or are under development. They work by blocking the binding of PD-L1/PD-1, enhancing the patient's immune system response to the tumor, thereby achieving the purpose of killing tumor cells.

Camrelizumab (code SHR-1210) is a recombinant humanized antibody independently developed by Jiangsu Hengrui Pharmaceuticals Co., Ltd., targeting PD-1.

Camrelizumab has been approved by the National Medical Products Administration (NMPA) of China in May 2019 for the treatment of patients with recurred or refractory classical Hodgkin Lymphoma who have received at least two lines of systemic chemotherapy. Based on a randomized, open-label, multicenter Phase 3 clinical study in China comparing camrelizumab with chemotherapy as a second-line treatment for advanced esophageal squamous cell carcinoma (ESCORT study)[7], it was officially approved by the NMPA on June 19, 2020, for patients with locally advanced or metastatic esophageal squamous cell carcinoma who have progressed or are intolerable after first-line chemotherapy.

In addition, for first-line advanced esophageal squamous cell carcinoma, a randomized, double-blind, placebo-controlled multicenter Phase 3 study in China—ESCORT 1st—announced its positive results at the 2021 ASCO meeting. In this study, subjects were randomized in a 1:1 ratio to receive either 200 mg of camrelizumab combined with chemotherapy or placebo combined with chemotherapy, with the primary endpoints being PFS and overall survival (OS). A total of 548 subjects were included in this study, and the results showed that the test group receiving camrelizumab combined with chemotherapy had significantly prolonged OS and PFS compared to the control group (mOS: 15.3 months vs. 12.0 months, HR = 0.70, P = 0.001; mPFS: 6.9 months vs. 5.6 months, HR = 0.56, P < 0.001), and patients in different subgroups all benefited from the treatment with camrelizumab.

Studies on camrelizumab as a monotherapy and in combination with chemotherapy or other therapeutic drugs are ongoing. For detailed information on preclinical and clinical studies of camrelizumab, please refer to the Investigator’s Brochure and the package insert.

1.3 Scientific Rationale for the Study

Available data suggest that the efficacy of conventional chemotherapy or radiotherapy has reached a plateau for perioperative treatment. Compared to conventional therapies, immunotherapy has made breakthrough progress in the second-line treatment for advanced and metastatic esophageal cancer, and several ongoing registration studies exploring the efficacy of immunotherapy in first-line treatment for advanced oesophageal cancer and unresectable locally advanced esophageal cancer have successively obtained positive results, providing a new perspective for perioperative treatment of esophageal cancer.

Several studies have already explored the efficacy of immune checkpoint inhibitors alone or in combination for neoadjuvant or adjuvant therapy. Pembrolizumab combined with neoadjuvant concurrent chemoradiotherapy for the treatment of patients with resectable esophageal squamous cell carcinoma[8] (clinical stage T1N1-2 or T2-4aN0-2, according to the 7th edition of AJCC), followed by postoperative adjuvant maintenance therapy with pembrolizumab monotherapy, showed that the immune checkpoint inhibitor combined with neoadjuvant chemoradiotherapy did not delay surgery, and did not increase the risk of surgical complications. The pathologcal complete response (pCR) rate of primary lesion was 46.1%, and the pCR rate of primary lesion and lymph node was 23.1%. Although the study did not reach the preset primary endpoint, the addition of pembrolizumab in preoperative neoadjuvant therapy showed promising efficacy and acceptable toxicity. For patients with locally advanced esophageal cancer, patients who underwent R0 resection after preoperative chemoradiotherapy received durvalumab monotherapy after surgery[9], and the 1-year relapse-free survival (RFS) rate was 79.2%, which was significantly higher compared to previous historical data (~50%). There are also several ongoing Phase 2 studies exploring the combination of camrelizumab with neoadjuvant chemotherapy for resectable locally advanced esophageal squamous cell carcinoma, with preliminary results showing controllable safety and promising efficacy (data not yet published).

Cytotoxic drugs release a large amount of tumor antigens while killing tumor cells and expose them to the body's immune system. Standard chemotherapy regimens combined with PD-1/PD-L1 inhibitors can increase tumor-specific T-cell immune activity, which is theoretically presumed to produce synergistic and lasting anti-tumor effects compared to standard chemotherapy regimens.

This study compares the efficacy of camrelizumab combined with chemotherapy for neoadjuvant therapy for patients with resectable locally advanced esophageal squamous cell carcinoma, in order to provide new options for perioperative treatment of locally advanced patients.

2. Study Objectives and Endpoints

2.1 Study Objectives

2.1.1 Primary objective

To compare the perioperative regimen of camrelizumab combined with neoadjuvant chemotherapy and postoperative camrelizumab adjuvant therapy versus neoadjuvant chemotherapy with paclitaxel and cisplatin for patients with resectable esophageal squamous cell carcinoma in terms of pathologcal complete response (pCR) and Event-Free Survival (EFS).

2.1.2 Secondary objective

To compare the perioperative regimen of camrelizumab combined with neoadjuvant chemotherapy and postoperative camrelizumab adjuvant therapy versus neoadjuvant chemotherapy with paclitaxel and cisplatin for patients with resectable esophageal squamous cell carcinoma in terms of major pathological response (MPR), R0 resection rate, pathological stage after neoadjuvant therapy (ypTNM stage), disease-free survival (DFS), overall survival (OS), and safety and tolerability.

2.1.3 Exploratory objectives

- To explore the quality of life of patients with resectable esophageal squamous cell carcinoma treated with the perioperative regimen of camrelizumab combined with

neoadjuvant chemotherapy and postoperative camrelizumab adjuvant therapy compared to the neoadjuvant chemotherapy with paclitaxel and cisplatin;

- To evaluate the relationship between biomarkers in tumor tissues and response;

2.2 Study Endpoints

2.2.1 Primary endpoints

- pCR assessed by Blinded Independent Review Committee (BIRC), defined as the proportion of subjects with no residual tumor in the primary tumor site (Mandard grade 1) and histologically negative lymph nodes;
- EFS assessed by the investigator according to RECIST 1.1, defined as the time from randomization to the occurrence of any of the following events, whichever comes first:
  - ➢ Radiographic tumor progression assessed by RECIST 1.1;
  - ➢ Tumor recurrence assessed by radiology or tissue biopsy, including local recurrence or distant metastasis (for subjects with no residual tumor after surgery);
  - ➢ Death due to any cause;
- Note: A second primary malignancy, or radiological progression occurring during the neoadjuvant phase that does not affect the radical resection of esophageal cancer, is not considered an EFS event.

2.2.2 Secondary endpoints

- MPR assessed by BIRC: The percentage of subjects with <10% residual tumor in the primary tumor site;
- R0 resection rate;
- Pathological stage after neoadjuvant therapy (ypTNM stage) based on the 8th edition of AJCC;
- OS: The time from randomization to death due to any cause;
- DFS: The time from the date of surgery who completed R0 resection to local or distant recurrence, or death due to any cause, whichever occurs first;

Safety:

- Adverse events (AEs): Incidence and grade (including serious adverse events [SAEs] and immune-related adverse events [irAEs]), determined according to the NCI-CTCAE 5.0 criteria;
- Surgery safety: Record surgical complications during the 30 days after surgery or during the hospitalization period, length of hospital stay, reoperation rate, and mortality rates at 30 days post-surgery and 90 days post-surgery, with the severity of surgical complications graded according to the Clavien-Dindo (CD) criteria;

Exploratory endpoints:

- Patient-reported outcomes (PRO): Quality of life and changes from baseline assessed from baseline to survival follow-up according to the European Organization for Research and Treatment of Cancer Quality of Life Questionnaire-C30 (EORTC QLQ-C30) and the Supplementary Questionnaire for Patients with Esophageal Cancer (EORTC QLQ-OES18);
- The relationship between potential biomarkers in tumor tissues (including but not limited to baseline PD-L1 expression (IHC, PD-L1 IHC 22C3 pharmDx kit, Dako, The assessment of PD-L1 expression involved both CPS and TPS), MMR status, TMB, EBER) and response; The CPS is defined as the number of PD-L1 staining cells divided by the total number of viable tumor cells, multiplied by 100; the TPS represents the percentage of viable tumor cells exhibiting membrane staining, assessed in a sample of at least 100 viable tumor cells.

3. Study Design

3.1 Overall design

This study is a multicenter, randomized, open, parallel-controlled clinical study in China to compare the efficacy and safety of camrelizumab combined with albumin-bound paclitaxel and cisplatin (Group A) or camrelizumab combined with paclitaxel and cisplatin (Group B) versus paclitaxel and cisplatin (Group C) in patients with histopathologically or cytologically confirmed resectable locally advanced thoracic esophageal squamous cell carcinoma.

Eligible patients will be randomized in a 1:1:1 ratio to test group A, test group B, or control group C, with pCR assessed by BIRC and EFS assessed by the investigator as primary endpoints, and 390 patients are planned to be enrolled.
A stratified block randomization method will be used in this study, with stratification factors including:

- Clinical stage (Stage I/II vs. Stage III vs. Stage IVa)

The study includes a screening period (no more than 21 days, from the signing of informed consent form [ICF] to the first dose), a treatment period (including neoadjuvant therapy, surgery, and postoperative adjuvant therapy) and a follow-up period (including safety follow-up, tumor progression/recurrence follow-up, and survival follow-up).

Neoadjuvant therapy:

- **Test group (Group A):** Camrelizumab combined with albumin-bound paclitaxel and cisplatin, 2 cycles, followed by surgical resection;
- **Test group (Group B):** Camrelizumab combined with paclitaxel and cisplatin, 2 cycles, followed by surgical resection;
- **Control group (Group C):** Paclitaxel and cisplatin, 2 cycles, followed by surgical resection;

During the study treatment period, a dosing time window of ± 3 days is allowed, but within 3 days before each dose, except for necessary radiological examinations, subjects should complete laboratory tests and physical examinations (as needed), ECOG scoring and other safety assessments to ensure tolerance before continuing with the study treatment (if the laboratory tests of screening period are completed within the protocol-specified pre-randomization time, then there is no need to retest before the first dose). The safety of subjects is continuously assessed throughout the study process.

Surgery:

- At any time during regular tumor assessment or neoadjuvant therapy, if disease progression is observed in a subject but the investigator judges the tumor to be still resectable, without distant metastasis, and the subject still meets the eligibility criteria for study treatment and evaluation, they may continue to receive surgery and subsequent treatment.
- Subjects who discontinue neoadjuvant therapy due to disease progression and no longer undergo surgery will discontinue the subsequent study treatment procedures and receive other treatment plans determined by the investigator. However, subjects will continue to receive safety follow-up and survival follow-up.
- For subjects whose tumor does not progress during neoadjuvant therapy but cannot undergo surgery due to other reasons, the investigator may determine the next step of the treatment plan with the subject. Subjects will still continue to receive safety follow-up and

survival follow-up.

- Subjects will be re-assessed by the investigator prior to surgery. Preoperative visits and related assessments should be performed within 14 days before surgery, and follow the treatment principles of each site. Surgery should be performed within 4-6 weeks of last dose. If surgical treatment exceeds 6 weeks of the last dose of neoadjuvant therapy, the preoperative visits and related assessments will be repeated within 14 days before surgery. After surgery, the surgical specimens will be assessed for pathological response by each site and BIRC.
- As for resection of esophageal cancer + complete thoracoabdominal two-field lymphadenectomy, the recommended surgical method is Mckeown procedure, and the complete thoracoabdominal two-field dissection is recommended for the lymphadenectomy.

Postoperative adjuvant therapy:

- **Test groups (Groups A and B):** Camrelizumab will be administered every 3 weeks as a dosing cycle, until radiological disease recurrence or metastasis, intolerable toxicity, initiation of other systemic anti-tumor therapy, withdrawal at the subject's own request, or withdrawal required by the investigator; the first dose of camrelizumab after surgery should be administered within 4-6 weeks postoperatively; during the entire study period, camrelizumab is administered for a maximum of 17 doses in total before and after surgery.

- **Control group (Group C):** Subjects in the control group will receive regular follow-up observation until disease recurrence or metastasis, withdrawal at the subject's own request, or withdrawal required by the investigator;

During the study treatment period, a dosing time window of ±3 days is allowed, but within 3 days before each dose, subjects should complete laboratory tests and/or necessary physical examinations, ECOG scoring, and other safety assessments to ensure they can still tolerate the study treatment. The safety of subjects is continuously assessed throughout the study process. Upon discontinuation of treatment, the subjects are required to undergo a comprehensive examination, including vital signs, physical examination, laboratory tests, and radiological tumor assessment.

For subjects who are pathologically assessed as non-R0 resected (R1/R2) after surgery, other treatment options determined by the investigator may be accepted according to clinical practice, but subsequent safety follow-up, tumor progression/recurrence follow-up, and survival follow-up should still be continued.

Radiological assessment:

Regular radiological response assessments will be performed according to RECIST 1.1 criteria, which include enhanced CT scans of the neck/chest/abdomen (CT scan slice thickness ≤5 mm) or enhanced MRI scans; it is recommended to add neck ultrasound for the diagnosis and differential diagnosis of metastatic lesions such as cervical lymph nodes; those suspected of having brain metastasis must also undergo enhanced brain MRI or enhanced CT to exclude brain metastasis; when metastases to bone are confirmed or clinically suspected, a bone scan is required; unless otherwise specified, the permissible window period for radiological examinations is ± 7 days. When disease progression is suspected (such as worsening of symptoms), unscheduled radiological examinations may be conducted.

- **At screening**, all subjects should undergo tumor radiological assessments; the tumor baseline assessments can be relaxed to within 3 weeks before randomization, and CT/MRI results obtained before signing the ICF can be used for the tumor assessment at screening if they meet the requirements of this protocol, and esophageal ultrasound endoscopy (EUS)

should be added when making clinical staging judgments;

- **After randomization,** the conditions for radiological examination should be the same as the baseline (including scan slice thickness, contrast agents, etc.). Tumor radiological assessments should be performed within 14 days before surgery (i.e., within 3-4 weeks after the last dose of neoadjuvant therapy, or within 14 days before surgery if surgery exceeds 6 weeks after the last dose of neoadjuvant therapy), at 4 weeks after surgery, and before receiving postoperative carelizumab treatment only for test groups A and B, then every 12 weeks (±14 days), and every 24 weeks (±28 days) starting from the third year, until radiological progression/recurrence, withdrawal of informed consent, loss to follow-up, or death, whichever occurs first.
- At the time of the first radiographic disease progression while receiving camrelizumab, if the subject's clinical status is stable as assessed by the investigator and the subject's informed consent is obtained, it is recommended to repeat radiological examination after an interval of 4-6 weeks for confirmation. If disease progression is not confirmed, tumor assessments will be performed at the original planned frequency. If it is confirmed, and the investigator judges that the subject continues to benefit, tumor assessments will continue at the frequency specified in the original protocol during subsequent treatment. If the subject's clinical symptoms are unstable, there is no need for further radiological confirmation.
- When a subject discontinus study treatment for reasons other than radiological disease progression, an radiological examination should be performed at the end of treatment (unless it has been performed within 28 days), and thereafter, whenever possible, at the same frequency until radiological progression/ recurrence, withdrawal of informed consent, loss to follow-up, or death.

Surgery assessment:

Surgery for esophageal cancer will be evaluated for the extent of radical resection, surgical complications, perioperative mortality (within 30 days post-surgery, within 90 days post-surgery), length of hospital stay, and rate of reoperation, among others;

Pathological evaluation:

Tumor regression grade (TRG) will be assessed based on the Mandard criteria, and yp staging is performed according to the 8th edition of AJCC.

Follow-up:

After the end of treatment, the subjects enter the follow-up period, which is divided into safety follow-up and survival follow-up. Subjects who discontinue treatment for reasons other than disease progression/recurrence should also be followed for tumor progression/recurrence.

Safety follow-up:

The end of the safety follow-up period is defined as 90 days after the last dose of camrelizumab for the test group, or 30 days after surgery for patients who have undergone surgery and 30 days after the last dose of study drug for patients who have not undergone surgery in the control group, or screening failure. Only SAEs considered related to the study drug are collected after the safety follow-up period. Subjects in the test group are followed up every 30 days starting from the last dose of camrelizumab until 90 days, through clinical visits or telephone interviews, to collect information on the subjects' survival status, AEs, concomitant medications, and concomitant treatments. The investigator may increase visits as needed for AE follow-up, with the aim of monitoring the resolution of AEs.

Tumor progression/recurrence follow-up:
For subjects who discontinue treatment for reasons other than disease progression/ recurrence, radiological examinations should continue at the frequency specified in the protocol to follow up on the time of disease progression/recurrence, until radiological progression/recurrence, withdrawal of informed consent, loss to follow-up, or death.
Survival follow-up:
After the end of study treatment, survival follow-up begins, occurring every 3 months, through clinical visits or telephone interviews, to collect survival information and information on post-study anti-tumor therapy and time of disease progression, until the subject's death, loss to follow-up, or study termination.

3.2 Methods to Reduce Bias

3.2.1 Enrollment procedures

This study employs a randomized, controlled design.

After obtaining written informed consent from the subjects, completing all screening procedures and assessments, and confirming the eligibility of the subjects, the study sites will obtain the subjects' identification numbers and treatment drug allocation information from the IVRS/IWRS.

3.2.2 Randomization

Eligible subjects, with an expected enrollment of 390 subjects, will be randomly assigned to test group A, test group B, or control group C in a 1:1:1 ratio. This study will use stratified block randomization, with the following stratification factors:

- Clinical stage: Stage I/II vs. Stage III vs. Stage IVa

The randomization of subjects will be completed through the RTMS system in this study. Whenever possible, patients should receive the first dose of study treatment at the time of randomization. Those who are unable to receive treatment on the day of randomization should receive the first dose within 3 working days after randomization.

Subjects who are not eligible for inclusion in the study should not be randomized under any circumstances.

3.2.3 Blinded independent pathological assessment

In this study, the BIRC will independently review all pathological slides and assess the grading of pathological regression according to the Mandard criteria. Each study site will submit postoperative pathological slides to the independent pathology review institution during the study or upon the sponsor’s request. Detailed rules and guidelines for the BIRC’s pathological remission assessment will be described in a separate operation manual.

3.3 Study Duration

The first subject enrollment for this study is expected in December 2020, and 390 patients will be enrolled nationwide for data collection over a period of 3 years. The total duration of the study is expected to be 7 years, with an anticipated study completion date of December 2027.

4. Study Population

4.1 Inclusion Criteria

Subjects must meet all of the following criteria to be enrolled in this study:

1) Having signed a written ICF, and voluntarily participating in this study;
2) Histopathologically or cytologically confirmed esophageal squamous cell carcinoma;
3) Thoracic esophageal cancer assessed by CT/MRI/EUS etc., with the clinical staged of T1b-3N1-3M0 or T3N0M0 (according to the 8th edition of AJCC);
4) Expected to achieve R0 resection;
5) Aged 18-75 years, male or female;
6) ECOG PS 0-1;
7) Having not received any prior anti-tumor therapy for esophageal cancer, including radiotherapy, chemotherapy, surgery, etc.;
8) Planned surgery after completion of neoadjuvant therapy;
9) No contraindication to surgery;
10) Normal major organ functions, including:
    a) Hematology (no use of any blood components, cell growth factors, leukocyte-elevating drugs, platelet-elevating drugs, or anaemia-correcting drugs within 14 days before the first use of study drug):
        - Neutrophil count ≥ 1.5×10^9/L
        - Platelet count ≥ 100×10^9/L
        - Haemoglobin ≥ 90 g/L
    b) Blood biochemistry:
        - Total bilirubin ≤ 1.5×ULN
        - ALT ≤ 2.5×ULN, AST ≤ 2.5×ULN,
        - Serum creatinine ≤ 1.5×ULN, or creatinine clearance ≥ 50 mL/min (Cocheroft-Gault formula)
    c) Coagulation function:
        - International normalized ratio (INR) ≤ 1.5×ULN
        - Activated partial thromboplastin time (APTT) ≤ 1.5×ULN
11) Female subjects of childbearing potential should have a negative serum pregnancy test within 72 hours before taking the study drug, and use effective contraception (e.g., intrauterine devices, contraceptives, or condoms) during the study and for at least 3 months after the last dose; male subjects with female partners of childbearing potential should be surgically sterile or agree to use effective contraception during the study and for 3 months after the last dose;

Subjects with good compliance, and cooperating with follow-up;

4.2 Exclusion Criteria

Subjects with any of the following conditions will not be enrolled in this study:

1) Tumor with obvious invasion into adjacent organs of the esophageal lesion (major arteries or trachea);
2) Presence of metastases to supraclavicular lymph nodes;
3) Presence of uncontrollable pleural effusion, pericardial effusion, or ascites requiring repeated drainage;
4) Poor nutritional status, BMI < 18.5 kg/m^2; if corrected after symptomatic nutritional support before randomization, these patients may be considered for enrollment after evaluation by the principal investigator;

5) History of allergy to monoclonal antibodies, any component of camrelizumab, paclitaxel, cisplatin, or other platinum-based drugs;
6) Prior or ongoing treatment with any of the following:
    a) Any radiotherapy, chemotherapy, or other anti-tumor drugs;
    b) Use of immunosuppressive drugs, or systemic corticosteroid therapy for immunosuppressive purposes (doses > 10 mg/day prednisone or equivalent) within 2 weeks prior to the first dose of study drug; inhaled or topical steroids and adrenal corticosteroid replacement at doses > 10 mg/day prednisone or equivalent are permitted in the absence of active autoimmune disease;
    c) Having received a live attenuated vaccine within 4 weeks prior to the first use of study drug;
    d) Major surgery or serious trauma within 4 weeks prior to the first use of study drug;
7) History of any active autoimmune disease or autoimmune disease, including but not limited to: interstitial pneumonia, enteritis, hepatitis, hypophysitis, vasculitis, nephritis, hyperthyroidism, hypothyroidism (considered after hormone replacement therapy); patients with psoriasis or childhood asthma/allergy that have resolved without any intervention in adulthood may be considered for inclusion, but patients requiring medical intervention with bronchodilators should not be included;
8) History of immunodeficiency, including HIV test positive, or presence of other acquired or congenital immune deficiencies, or history of organ transplantation or allogeneic bone marrow transplantation;
9) Subject with clinically uncontrolled cardiac symptoms or diseases, including but not limited to: (1) heart failure of NYHA class II or above, (2) unstable angina pectoris, (3) myocardial infarction within 1 year, (4) clinically significant supraventricular or ventricular arrhythmias without clinical intervention or uncontrolled with clinical intervention;
10) Serious infection (CTCAE > Grade 2) within 4 weeks prior to the first dose of study drug, such as severe pneumonia, bacteraemia, and infection complications requiring hospitalization; active pulmonary inflammation based on chest X-ray at baseline; symptoms and signs of infection requiring oral or intravenous antibiotic therapy within 14 days prior to the first dose of study drug, except prophylactic use of antibiotics;
11) Patients with active tuberculosis infection based on medical history or CT test, or patients with a history of active tuberculosis infection within 1 year before enrollment, or patients with active tuberculosis infection beyond 1 year but without proper treatment;
12) Presence of active hepatitis B (HBV DNA ≥ 2000 IU/mL or 10^4 copies/mL) or hepatitis C (positive for hepatitis C antibody and HCV RNA above the lower limit of detection of the assay);
13) Other malignancies diagnosed within 5 years prior to the first dose of study drug. Except malignancies with low risk of metastasis or death (5-year survival rate > 90%), such as adequately treated basal cell carcinoma of skin or squamous cell skin cancer or carcinoma in situ of the cervix;
14) Pregnant or lactating women;

Presence of other factors that may lead to forced withdrawal from the study as judged by the investigator, such as other serious diseases (including psychiatric disorders) requiring concomitant therapy, alcoholism, drug abuse, family or social factors, which may affect the safety or compliance of the subjects.

4.3 Discontinuation of Treatment/Withdrawal from Study/Study Termination

4.3.1 Discontinuation of treatment/withdrawal from study

Subjects may withdraw informed consent at any time for any reason. If an adverse event (AE) occurs, the investigator may decide to discontinue the subject's study treatment. In addition, if the subject is ineligible based on the inclusion and exclusion criteria, violates the study protocol, or for administrative and/or other safety reasons, the investigator may discontinue the subject's treatment and withdraw the subject from the study.

Discontinuation of study treatment does not mean that the patient has withdrawn from the study; as long as the subject has not withdrawn the informed consent and refused further follow-up, the subject is still required to complete the remaining study visits as required by the protocol after discontinuing treatment.

The subject must discontinue treatment but continue follow-up in any of the following situations:

- Having completed all protocol-specified treatments;
- Withdrawal of informed consent, refusing to continue receiving study drug treatment;
- Worsening of clinical symptoms or decline of physical condition as judged by the investigator;
- Radiological disease progression/tumor recurrence or metastasis, unless subjects in the test group meet the criteria for continued treatment after disease progression and agree to continue treatment;
- Occurrence of intolerable AEs, or other events affecting the safety of the subject, including but not limited to any clinical AEs, laboratory abnormalities, or other medical conditions;
- Significant protocol deviations;
- Other situations deemed necessary by the investigator to discontinue study drug treatment;
- Subject request;
- Pregnancy;
- Loss to follow-up;
- Initiation of other systemic anti-tumor therapy.

The subject will be withdrawn from the study in any of the following situations:

- Subject's withdrawal of informed consent, refusing further follow-up;
- Loss to follow-up;
- Death;
- Termination of the study;
- Other situations deemed necessary by the investigator for withdrawal from the study.

The main reason for the discontinuation of subject's treatment should be recorded in the appropriate case report form (CRF).

If a subject is found to be ineligible after enrollment, the investigator must discuss with the principal investigator (sponsor) whether the subject should continue or withdraw from the study.

4.3.2 Steps for withdrawal or discontinuation of treatment

If the subject does not appear for a scheduled visit, every effort should be made to contact them. Under any circumstances, the final status of the subject should be recorded as completely as possible. The investigator should inquire about the reasons for withdrawal, request a visit from the subject, and make every effort to complete the efficacy and safety examinations specified

in the protocol at the time of withdrawal, as well as complete the safety follow-up period, and fully record AEs and their outcomes. The investigator may suggest or provide new or alternative treatments to the subject based on the actual situation of the subject. Subjects without disease progression should continue to be followed with radiological assessments until radiological disease progression occurs.

It should be noted that, unlike withdrawal from the study, subjects who request discontinuation of study treatment will remain on study, and should be encouraged to complete follow-up according to the treatment plan. If a subject refuses further visits, their survival status should still be followed up, unless the subject withdraws consent for further information disclosure or to be contacted again. In this case, no further study evaluations should be conducted, nor should any more data be collected. All data collected prior to the subject's withdrawal of informed consent will continue to be retained and used, unless the subject requests the withdrawal of the information already collected.

4.3.3 Early termination or suspension of the study

The study may be terminated or suspended early if there are sufficient reasons. This could be due to changes in the opinion of the Ethics Committee, issues with the efficacy or safety of the study drug, etc. Once the decision to suspend/terminate the study is made, the investigator must immediately notify the Ethics Committee and provide the relevant reasons.

Reasons for early termination or suspension of the study may include:

- Identified unexpected, significant, or unacceptable risks to the subjects.
- Existing efficacy results, which support early termination of the study.
- Significant errors found in the protocol during the implementation of the study.
- Great difficulty in completing the study, and low compliance with protocol requirements due to reasons such as severe lagging in subject enrollment or frequent protocol deviations.
- Ineffectiveness of the study drug/study treatment, making it meaningless to continue the study;
- Incomplete or inaccurate data.

Once the drug safety, protocol compliance, and data quality issues leading to study suspension mentioned above are resolved, the study may proceed with the approval of the Ethics Committee.

Furthermore, if a site experiences difficulties in patient recruitment, and non-compliance with the protocol, GCP and/or other applicable regulations, or has a high number of subjects who discontinue the study due to management issues, consideration may be given to limiting further subject enrollment at the site or to closing the site.

5. Investigational Medicinal Products

5.1 Overview of Study Drugs

The following study drugs are all uniformly provided by the sponsor, Jiangsu Hengrui Pharmaceuticals Co., Ltd.

Drug Name	Short Name	Strength
Camrelizumab for Injection	Camrelizumab	200 mg/vial
Paclitaxel for Injection (albumin-bound)	Albumin-bound Paclitaxel	100 mg/vial
Paclitaxel Injection	Paclitaxel	5 mL: 30 mg
Cisplatin Injection	Cisplatin	6 mL: 30 mg

5.2 Packaging and Labeling

The sponsor will package the study drugs in accordance with applicable regulatory requirements and label them as required.

5.3 Preparation of Study Drugs

Study drugs should be prepared by qualified or experienced study personnel according to the package insert.

5.4 Treatment Plan

5.4.1 Dosing regimen

The dose and frequency of administration for each study treatment component are detailed in the table below:

Therapeutic Drug	Dose	Route of Administration	Dosing Cycle
**Neoadjuvant Therapy**			
Camrelizumab	200 mg/dose	IV	D1, Q3W for 2 cycles
Albumin-bound Paclitaxel	125 mg/m^2	IV	D1/D8, Q3W, for 2 cycles
Paclitaxel	175 mg/m^2	IV	D1, Q3W for 2 cycles
Cisplatin	75 mg/m^2	IV	D1, Q3W for 2 cycles
**Postoperative Adjuvant Therapy (only for subjects in test groups A and B)**			
Camrelizumab	200 mg/dose	IV	D1, Q3W;up to a total of 17 doses preoperatively and postoperatively during the entire study treatment period.

Neoadjuvant therapy: All eligible subjects will receive drug infusions in the following order according to their group:

- Test Group A: Camrelizumab → Albumin-bound Paclitaxel → Cisplatin on Day 1, with at least a 30 min interval; Albumin-bound Paclitaxel on Day 8;
- Test Group B: Camrelizumab monoclonal antibody → Paclitaxel → Cisplatin on Day 1, with at least a 30 min interval;
- Test Group C: Paclitaxel → Cisplatin on Day 1, with at least a 30 min interval;

Postoperatively, subjects in Test Groups A and B will receive camrelizumab monoclonal antibody monotherapy as adjuvant treatment, once every 3 weeks. Camrelizumab monoclonal antibody adjuvant treatment should begin within 4-6 weeks after surgery. From the start of neoadjuvant therapy, the maximum duration of camrelizumab monoclonal antibody use throughout the study is up to a total of 17 doses before and after surgery.

Subjects in the control group will receive regular follow-up after surgery.

5.4.1.1 Camrelizumab

It is recommended that camrelizumab monoclonal antibody be administered via an infusion pump, by intravenous infusion, lasting about 30-60 minutes. Intravenous bolus or rapid bolus is not permitted. At the end of the infusion, flush the infusion line with an adequate amount of saline or 5% glucose solution (according to the medical practice of each site).

5.4.1.2 Chemotherapy drugs

Before and after receiving chemotherapy drugs, antiemetic treatment should be given in

accordance with clinical practice and local treatment principles. Due to the immunomodulatory effects of corticosteroids, the use of corticosteroids to prevent nausea and vomiting should be avoided as much as possible.

Albumin-bound Paclitaxel

Albumin-bound paclitaxel will be administered at 125 mg/m^2 on Day 1 and Day 8 of each 3-week cycle. Before infusion, the lyophilized powder of albumin-bound paclitaxel must be dissolved in 0.9% sodium chloride injection solution, with a final concentration of 5 mg/mL. It is recommended that the infusion time for albumin-bound paclitaxel be limited to 30 minutes to reduce the possibility of infusion reactions. After administration, flush the vein with 0.9% sodium chloride injection solution.

For more information, please refer to the package insert of albumin-bound paclitaxel.

Paclitaxel

Paclitaxel will be administered on Day 1 of each 3-week cycle, with a total dose of 175 mg/m^2.

To prevent allergic reactions, dexamethasone, diphenhydramine, and H2 receptor antagonists (Cimetidine or Ranitidine) should be administered before paclitaxel treatment. Before infusion, paclitaxel should be diluted with 0.9% sodium chloride injection solution or 5% glucose injection solution, using disposable non-polyethylene material for the infusion bottle and infusion line, and filtered through the connected filter before intravenous drip, with a recommended infusion time of 3 hours.

For more information, please refer to the package insert of paclitaxel.

Cisplatin

Cisplatin will be administered on Day 1 of each 3-week cycle, with a total dose of 75 mg/m^2.

Before infusion, cisplatin should be diluted with 0.9% sodium chloride injection solution or 5% glucose injection solution, with adequate hydration and diuresis to prevent the renal toxicity of the product.

For more information, please refer to the package insert of cisplatin.

5.4.2 Surgery

The subject's eligibility for surgical resection should be assessed by an experienced thoracic surgeon at screening. At screening, subjects should meet the criteria for R0 resection with curative intent.

During regular tumor assessments (after the end of Cycle 2) or at any time during neoadjuvant therapy, if disease progression is observed but the investigator judges the tumor to be resectable, without distant metastasis, and the subject still meets the eligibility criteria for study treatment and evaluation, they may continue to receive surgery and subsequent treatment. Subjects who discontinue neoadjuvant therapy early due to disease progression and no longer undergo surgery will discontinue the subsequent study procedures and receive other treatments determined by the investigator. These subjects will receive survival follow-up. For subjects whose tumor does not progress during neoadjuvant therapy but who are unable to undergo surgery for other reasons, the investigator will confirm the next treatment plan with the subject.

The investigator must reassess the subject after the completion of neoadjuvant therapy and before surgery. Preoperative visits and related assessments should be performed within 14 days before surgery, including but not limited to blood tests, coagulation, cardiac assessment, anesthesia assessment, and other assessment procedures, following the treatment principles of

each study site. Surgery should be performed within 4-6 weeks after the last dose of neoadjuvant therapy. If surgery cannot be performed within 6 weeks (for example, due to ongoing AEs), it should be discussed with the principal investigator (sponsor). If surgery is planned after 6 weeks from the last neoadjuvant therapy, the preoperative visits and related assessments, as well as radiographical scans, should be repeated within 14 days before the planned surgery.

For those who can tolerate surgery, esophagectomy + complete thoracoabdominal two-field lymphadenectomy is recommended, with the Mckeown procedure being the recommended approach. Complete thoracoabdominal two-field lymphadenectomy is recommended for lymph node dissection. The scope of lymphadenectomy is recommended to include lymph nodes adjacent to the left/right cardia, lesser curvature of the stomach, left gastric artery, alongside the celiac artery, diaphragm, alongside the lower/middle/upper esophagus, below the carina, and adjacent to the left/right recurrent laryngeal nerve, and to mark the lymph node dissection locations and send them for pathological examinatoin, with a minimum of 15 lymph nodes recommended for radical purpose and accurate staging.

The surgical procedures performed should be recorded and reported in the CRF. If the surgeon determines that the subject is not suitable for the planned surgery after neoadjuvant therapy or during surgery, the reasons should be recorded and reported in the CRF.

5.4.3 Continued medication after disease progression

More and more evidence suggests that despite initial evidence of disease progression, some subjects treated with immunotherapy may still derive clinical benefit. In addition, disease progression (first radiological finding) does not necessarily indicate treatment failure, so conventional response criteria may not adequately assess the effect of immunotherapy drugs. Therefore, during the study, if subjects exhibit radiological evidence of disease progression according to RECIST 1.1 criteria, and the investigator, combining radiological, biopsy (if possible), and clinical status, assesses that they can continue to derive clinical benefit (without unacceptable toxicity or significant symptom worsening related to disease progression), they may continue to receive treatment with camrelizumab, with the following criteria:

1. The investigator judges that continuing treatment is in the best interest of the subject;
2. The subject's disease condition is stable and does not require immediate initiation of other anti-tumor therapy;
3. The subject can tolerate continued treatment;
4. There is no significant decline in the subject's physical condition, and no significant worsening of tumor-related symptoms;
5. Continuing treatment will not delay urgently needed interventions to prevent serious complications due to disease progression;
6. Before continuing treatment, the subject must re-consent, with the informed consent form describing possible risks, discomfort, and other treatment options;
7. Continuation of treatment is reviewed and approved by the principal investigator of this study site;

Clinical benefit assessment must consider whether the subject is clinically deteriorating and whether they can benefit from continued treatment; if the subject continues treatment, they should continue to undergo various examinations according to the Schedule of Activities; Since no absolute distinction can be made between pseudoprogression and true tumor progression, there may be a certain risk that subjects may continue to receive treatment with camrelizumab despite its ineffectiveness, which may lead to further disease progression. After initial disease

progression judged according to RECIST 1.1 criteria, it is recommended that the investigator should arrange a radiological examination for the subject again after 4-6 weeks if the subject's clinical symptoms are stable. If disease progression is not confirmed, tumor assessments should still be performed at the original planned frequency; if disease progression is confirmed, tumor assessments will continue at the frequency specified in the original protocol during subsequent treatment. If clinical symptoms are unstable, there is no need for further radiological confirmation. if disease progression is observed in the subject's next tumor assessment, and the investigator judges that the subject can no longer benefit from continuing treatment, they should be withdrawn from the study treatment;

5.5 Dose Modification and Delay

5.5.1 General principles of dose modification

The investigator should carefully assess the severity of toxicity and risk-benefit for the subject, referring to the following guidelines to modify or delay the dose of the study drug, with the aim of ensuring the subject's safety and maximizing patient compliance. The reasons for dose modification or delay, the actions taken, and the results should be recorded in the subject's medical records and CRF. The severity of AEs will be graded according to the NCI-CTCAE V5.0 grading system;

The dosing window period is ±3 days from the planned dosing date (calculated as the date of the first dose). If the dosing window period (3 days) is exceeded, it will be considered a delayed dosing, and the subsequent dosing dates will be recalculated based on the actual date of last dosing. In cases where dosing is delayed due to toxicity (which cannot be clearly related to any particular drug), all three drugs need to be delayed simultaneously, and upon resumption of dosing, the entire combination regimen should be administered in the original prescribed order as much as possible, if feasible. However, during the combination period, chemotherapy is allowed to be withheld for up to 9 consecutive weeks, and chemotherapy should be terminated if it exceeds 9 weeks. In principle, camrelizumab can be withheld for up to 12 consecutive weeks, and if it exceeds 12 weeks, camrelizumab should be terminated unless the investigator believes that the subject can still benefit from camrelizumab treatment.

In cases not explicitly specified in the protocol, the investigator must consider the benefit/risk ratio for the subject before making a decision. For situations that are clearly defined in the protocol, medication should be temporarily suspended and the dosage reduced as stipulated. If some drugs are discontinued, the dose of another drug may not be down-titrated.

Investigators should be vigilant for early and significant signs of myelosuppression, neutropenia with fever, infection, or other chemotherapy-induced toxicity symptoms, so as to treat these complications promptly and appropriately. Subjects must be reminded of the potential occurrence of the aforementioned AEs and encouraged to seek medical attention as soon as possible.

5.5.2 Dose modification of camrelizumab

Based on the severity of toxicity and the risk-benefit assessment of the subject, the investigator may consider the modification principles introduced below at their discretion. It is not permitted to increase or decrease the dose of camrelizumab, only to suspend or terminate treatment. If camrelizumab is suspended for more than 12 weeks during the study due to immune-related toxicity and cannot be reduced to ≤ Grade 1 or baseline level, permanent discontinuation of camrelizumab will be considered, unless the investigator believes that the subject can still benefit from the treatment.

The time window for each dose during the neoadjuvant therapy combination period is

calculated from the date of the first dose. Delays should generally not exceed 3 days (subsequent cycles proceed as originally planned), and in special circumstances, reasons may be noted for exceeding 3 days.

The time of each dose during adjuvant monotherapy is calculated from the date of the first dose of adjuvant therapy. Delays should generally not exceed 3 days (subsequent cycles proceed as originally planned), and in special circumstances, reasons may be noted for exceeding 3 days. If the delay exceeds 3 days, it is recommended to recalculate the time window for the next dose based on the actual time of this dose, and the subsequent dosing timing window remains 3 days.

Immune-related adverse events (irAEs) refer to AEs of unknown etiology, related to drug exposure, and consistent with immune phenomena, which may occur shortly after the first dose or months after the last dose. Before identifying an event as an irAE, other causes such as tumor, infection, metabolic disorders, poisoning, or other diseases should be ruled out as much as possible. Immunological, serological, and histological (biopsy) data should be used to support the diagnosis of immune-related toxicity reactions. IrAEs most commonly occur in the skin, colon, endocrine organs, liver, and lungs. Although other organs and tissues are rarely involved, they may be relatively more severe or even life-threatening, such as nervous system disorders and myocarditis. It is recommended to follow the table below for delaying or interrupting camrelizumab treatment. For more information, please refer to the package insert of camrelizumab.

In addition, disease progression as assessed by RECIST 1.1 criteria (unless the subject meets the criteria for continuing treatment after progression, refer to Protocol 5.4.3) should lead to permanent discontinuation of treatment. Subjects are allowed to interrupt study treatment due to other medical or surgical events unrelated to the study treatment, or accidents (holidays), and should resume study treatment within 2 weeks after the interruption (the planned dosing time for this cycle), unless otherwise decided upon discussion by the investigator. The reasons for interrupting treatment must be recorded in the CRF.

Table (1) Dose Modifications of Camrelizumab Due to Immune-related Toxicity

**Camrelizumab-related Toxicity**	**Severity**	**Adjusted Regimen**
Diarrhoea/Colitis	Grade 2-3	Temporarily suspend until the adverse reaction recovers to Grades 0-1
	Grade 4	Dose permanently discontinued
AST/ALT or bilirubin increased	Grade 2, AST/ALT at 3-5 × ULN or TBIL at 1.5-3 × ULN	Temporarily suspend until the adverse reaction recovers to Grades 0-1
	Grade 3 or 4, AST/ALT >5 × ULN or TBIL >3 × ULN	Dose permanently discontinued
Nephritis	Grade 2-3 blood creatinine increased	Temporarily suspend until the adverse reaction recovers to Grades 0-1
	Grade 4 blood creatinine increased	Dose permanently discontinued
Endocrine disorders	Grade 2-3 symptomatic hypothyroidism<br>Grade 2-3 hyperthyroidism<br>Grade 2-3 hypophysitis<br>Grade 2 adrenal insufficiency<br>Grade 3 hyperglycaemia or diabetes mellitus	Temporarily suspend until the adverse reaction recovers to Grades 0-1
	Grade 4 hypothyroidism<br>Grade 4 hyperthyroidism<br>Grade 4 hypophysitis	Dose permanently discontinued

Camrelizumab-related Toxicity	Severity	Adjusted Regimen
	Grade 3-4 adrenal insufficiency<br>4 Grade 4 hyperglycaemia or diabetes mellitus	
Pneumonitis	Grade 2	Temporarily suspend until the adverse reaction recovers to Grades 0-1
	Grade 3-4 or recurrent Grade 2	Dose permanently discontinued
Infusion reactions	Grade 2	Reduce the infusion rate or withhold, and the study drug may be resumed when there is a symptomatic remission, closely observation should also be conducted.
	Grade 3-4	Dose permanently discontinued
Others	Grade 3-4 blood amylase increased or lipase increased<br>Grade 2-3 pancreatitis<br>Grade 2 myocarditis<br>Other immune-related Grade 2-3 adverse reactions that occur for the first time	Temporarily suspend until the adverse reaction recovers to Grades 0-1
	Grade 4 pancreatitis<br>Grade 3-4 myocarditis<br>Grade 3-4 encephalitis<br>Other immune-related Grade 4 adverse reactions that occur for the first time	Dose permanently discontinued

5.5.2.1 Toxicity management of irAEs

The grading management principles of irAEs are as follows:

Table (2) Grading Management Principles of Immune-related Toxicity

Grade	Corticosteroids	Other Immunosuppressants	Treatment with ICIs
Grade 1	Not recommended	Not recommended	Continued
Grade 2	Topical corticosteroids, oral prednisone 0.5-1.0 mg/(kg□d)	Not recommended	Withheld*
Grade 3	Systemic corticosteroids, oral prednisone or IV methylprednisolone 1-2 mg/(kg□d)	If symptoms do not resolve after 3-5 days of corticosteroid treatment, consider using under the guidance of a specialist	Discontinued, discuss whether to resume ICI treatment based on the risk/benefit ratio of the subject
Grade 4	Systemic corticosteroid treatment, IV methylprednisolone 1-2 mg/(kg□d) for 3 consecutive days, taper to 1 mg/(kg □ d) for maintenance if symptoms resolve, then gradually taper off over about 6 weeks until discontinuation	If symptoms do not resolve after 3-5 days of corticosteroid treatment, consider using under the guidance of a specialist	Permanently discontinued

*If only skin or endocrine symptoms are present, continued use may be considered.

5.5.2.2 Management of infusion reactions

If an infusion reaction occurs, it should be managed according to the situation by slowing down or interrupting the infusion, providing clinical supportive care, and administering prophylactic medication before subsequent treatments. Related symptoms and signs of acute infusion-related reactions (including angioedema, anaphylactic shock and allergic reactions; refer to NCI CTCAE v5.0 for relevant terms and criteria) usually occur during or immediately after drug infusion, and disappear within 24 hours after the completion of infusion. Symptoms and signs include allergic reactions/hypersensitivity reactions (including drug-induced fever), cough, fear of cold, chills/shivering, dizziness, headache, fatigue (asthenia), rash, pruritus cutaneous/pruritus, joint pain, muscle pain, low or high blood pressure, nausea, vomiting, sweating, tachycardia, urticaria (rubella), dyspnea (shortness of breath) or bronchial spasm.

The management of allergic reactions should be based on the medical practice and guidelines of the study site.

The management of delayed-type hypersensitivity (such as itching that occurs 1 week after the end of infusion) should involve symptomatic treatment (such as oral antihistamines or corticosteroids).

5.5.2.3 Reactive capillary endothelial proliferation

Reactive capillary endothelial proliferation (RCEP) mostly occurs on the surface of the skin, and is less common in oral mucosa, nasal mucosa, and eyelid conjunctiva. The following grading criteria and treatment recommendations can be referred to for management:

Grade	Clinical Manifestation	Treatment Recommendation
Grade 1	Single maximum diameter ≤10 mm with or without ulceration and bleeding	Dose unchanged;<br>Gauze can be used for protection in areas prone to friction to avoid bleeding. The area with ulceration and bleeding can be treated with local compression hemostasis.
Grade 2	Single maximum diameter >10 mm with or without ulceration and bleeding	Dose unchanged;<br>Gauze can be used for protection in areas prone to friction to avoid bleeding. The area with ulceration and bleeding can be treated with local compression hemostasis, or local treatment measures, such as laser or surgical resection, to avoid infection at the site of ulceration.
Grade 3	Generalized, can be complicated by skin infection, may require hospitalization	Hold the drug until recovery to ≤ Grade 1 or baseline level; resume medication thereafter;<br>Gauze can be used for protection in areas prone to friction to avoid bleeding. The area with ulceration and bleeding can be treated with local compression hemostasis, or local treatment measures, such as laser or surgical resection, and anti-infective treatment should be given for those complicated with infection.
Grade 4*	Multiple and generalized, life-threatening	Dose permanently discontinued
Grade 5*	Death	

When the subject experiences this adverse reaction, scratching or friction should be avoided, and gauze can be used for protection in areas prone to friction to avoid bleeding. The area with ulceration and bleeding can be treated with local compression hemostasis, or local treatment measures, such as laser or surgical resection should be given for those with recurrent bleeding. Local anti-infective therapy can be given to those with concurrent infections. See the package

insert of camrelizumab for details.

5.5.3 Dose modification of chemotherapy drugs

This study allows for dose modification of albumin-bound paclitaxel, paclitaxel, and cisplatin in response to chemotherapy-related AEs. Dose modifications for chemotherapy can be made with reference to the following text or as decided by the investigator according to clinical routine. If the AE is related only to one drug, only that drug's dose may be adjusted. For chemotherapy-related AEs not listed in the table below, the investigator may refer to the package insert or the treatment standards of the site to provide appropriate treatment to the subjects.

Chemotherapy can only be started on Day 1 of each cycle when the absolute neutrophil count (ANC) ≥ 1.5×10^9/L and platelets (PLT) ≥ 100 $\times10^9$/L. To provide sufficient recovery time, if chemotherapy is suspended for more than 9 weeks due to an AE and cannot be reduced to Grade 1 toxicity or baseline level, discontinuation of chemotherapy will be considered. Growth factor therapy can be used during chemotherapy. After recovery, at the start of subsequent treatment cycles, dose modifications will be based on the nadir ANC and PLT of the previous cycle. If the subject requires dose modifications for both ANC and PLT, it is recommended to receive a lower dose.

Table (3) Dose Modifications for Hematological Toxicity

Toxicity	Number of Occurrences	Albumin-bound Paclitaxel (mg/m^2)	Paclitaxel	Cisplatin
Febrile neutropenia	First occurrence	100	75% of planned dose	75% of planned dose
	Recurrence	75	NA	50% of planned dose
	Third occurrence	Discontinue treatment	NA	NA
Lowest PLT < 50 × 10^9/L	First occurrence	100	75% of planned dose	75% of planned dose
	Recurrence	Discontinue treatment	NA	Discontinue treatment
Lowest PLT<50×10^9/L with Grade 2 bleeding event	First occurrence	75	50% of planned dose	50% of planned dose
	Recurrence	Discontinue treatment	NA	Discontinue treatment

Interstitial lung tissue inflammation was observed in < 1% of patients treated with albumin-bound paclitaxel monotherapy and 4% of patients treated with albumin-bound paclitaxel in combination with gemcitabine. Therefore, signs and symptoms of pneumonitis should be monitored in patients. After excluding an infectious cause and confirming pneumonitis, albumin-bound paclitaxel should be permanently discontinued. Immediate initiation of appropriate treatment and supportive care is required. After excluding an infectious cause, high-dose corticosteroids should be administered immediately, along with appropriate premedication to prevent secondary infection.

Dose modification recommendations for patients with hepatic and renal impairment during the study treatment can be seen in the table below. Hepatotoxicity should recover to baseline level and nephrotoxicity should fully recover or decrease to Grade 1 prior to dosing.

Table (4) Dose Modifications for Hepatic and Renal Impairment

Toxicity	Number of Occurrences	Albumin-bound Paclitaxel (mg/m^2)	Paclitaxel	Cisplatin
Mild hepatic impairment (total bilirubin >1 to ≤1.5×ULN and ALT/AST <1.5×ULN)	-	100	75% of planned dose	75% of planned dose
Moderate hepatic impairment (total bilirubin 1.5-5.0×ULN and/or ALT/AST 5-10×ULN)	First occurrence	100	75% of planned dose	75% of planned dose
	Recurrence	50	NA	50% of planned dose
	Third occurrence	Discontinue treatment	NA	Discontinue treatment
Severe hepatic impairment (total bilirubin >5×ULN or ALT/AST >10×ULN	First occurrence	Discontinue treatment	Discontinue treatment	Discontinue treatment
Renal impairment (creatinine clearance 15-50 mL/min)	First occurrence	100	75% of planned dose	75% of planned dose
	Recurrence	50	NA	50% of planned dose
	Third occurrence	Discontinue treatment	NA	Discontinue treatment

In the event of Grade 3 or 4 toxicities not mentioned above, treatment with albumin-bound paclitaxel, paclitaxel and/or cisplatin should be suspended until the subject fully recovers or the toxicity reduces to Grade 1 or baseline level. When the toxicity recovers to Grade 1 or baseline level, treatment with albumin-bound paclitaxel, paclitaxel and/or cisplatin may be resumed but at a reduced dose (refer to the table). If the toxicity does not recover to Grade 1 or baseline level within 9 weeks, it is recommended to discontinue treatment with albumin-bound paclitaxel, paclitaxel and/or cisplatin. For Grade 1 and 2 toxicities, dose reduction is not recommended.

When diarrhoea, nausea, or vomiting occurs, appropriate antidiarrheal and antiemetic medications should be used for control. If the subject can tolerate it, the original dose should be resumed as soon as possible.

Table (5) Dose Modifications for Other Non-hematologic Toxicities

Toxicity	Number of Occurrences	Albumin-bound Paclitaxel (mg/m^2)	Paclitaxel	Cisplatin
Grade 3-4 diarrhoea or Grade 3-4 mucositis/oral inflammation or Grade 3-4 nausea/vomiting	First occurrence	100	75% of planned dose	75% of planned dose
	Recurrence	50	NA	50% of planned dose
	Third occurrence	Discontinue treatment	NA	Discontinue treatment
Grade 2 neurotoxicity	First occurrence	-	-	75% of planned dose
	Recurrence	-	-	50% of planned dose
	Third occurrence	-	-	Discontinue treatment
Grade 3-4 sensory neuropathy	First occurrence	100	75% of planned dose	50% of the planned dose or discontinue treatment
	Recurrence	50	NA	
	Third	Discontinue	NA	

	occurrence	treatment		
Other Grade 3-4 non-hematologic toxicities (excluding alopecia)	First occurrence	100	75% of planned dose	75% of planned dose
	Recurrence	50	NA	50% of planned dose
	Third occurrence	Discontinue treatment	NA	Discontinue treatment

5.5.4 Management, dispensing and recovery of drugs

The management, dispensing, and recovery of the study drugs are the responsibility of designated personnel at each study site, and investigators must ensure that all study drugs are used only for subjects participating in this study, with dosage and administration following the study protocol. Remaining study drugs must be returned to the sponsor. Leftover drug solutions should be destroyed directly according to medical waste standards. Study drugs should be stored as required in the package inserts.

The study monitor is responsible for overseeing the supply, use and storage of study drugs, as well as management of unused study drugs.

5.6 Concomitant Medications and Concomitant Therapies

Concomitant medications/concomitant therapies refer to other drugs/therapies other than the study treatment given at the discretion of the investigator for the purpose of a subject's interests.

All concomitant medications and therapies will be collected from the time of signing the ICF until the end of the safety follow-up period (90 days after the last dose of camrelizumab for the test group; 30 days after surgery for patients who have undergone surgery or 30 days after the last dose of study drug for patients who have not undergone surgery in the control group), including concomitant medications and therapies related to surgical complications up to 90 days post-surgery for subjects who have undergone surgery (if the patient starts a new anti-tumor therapy before the end of the safety follow-up period, only concomitant medications and therapies for AEs/SAEs suspected to be related to the study drug or fatal will be collected after the new anti-tumor therapy); after the safety follow-up period, investigators are not required to proactively record concomitant medications unless they are used for treating SAEs related to the study drug.

Drugs or vaccines explicitly prohibited in the study protocol are forbidden throughout the study; if a subject develops a concomitant disease that necessitates the use of prohibited drugs, discontinuation of study drug or administration of prohibited drugs may be required, and the investigator needs to discuss with the principal investigator (sponsor).

In addition to the following, standard chemotherapy treatment for subjects should also refer to the package insert and clinical practice for contraindications and precautions.

5.6.1 Permitted concomitant therapies

Palliative care and supportive care for underlying diseases and symptom management are allowed during the study for subject health concerns. Palliative and supportive care for disease-related symptoms will depend on the investigator's judgment and relevant guidelines. During the study, subjects should receive the best supportive care, such as supportive therapies (e.g., granulocyte colony-stimulating factors, blood transfusion) for chemotherapy-related myelotoxicity as determined by the investigator.

Patients with infusion-related symptoms should receive drug therapy according to the

symptoms with acetaminophen, ibuprofen, diphenhydramine, and/or H2 receptor antagonists (e.g., famotidine, cimetidine) or equivalent drugs, following local standard of care principles. For serious infusion-related events, such as dyspnoea, hypotension, wheezing, bronchospasm, tachycardia, oxygen saturation decreased, respiratory distress, etc., supportive care (e.g., supplemental oxygen inhalation and β2 adrenergic agonists) should be provided according to clinical indications.

Pre-existing hormone replacement therapy at physiological dose is allowed. For example, subjects who are currently receiving gonadotropin-releasing hormone (GnRH agonists) and have tolerated GnRH agonists well for at least 3 months before enrollment can enter this study. Patients with a history of autoimmune-mediated hypothyroidism who are receiving a stable dose of thyroid replacement hormone therapy may be enrolled in this study; patients with type I diabetes mellitus who are on a stable insulin regimen and have controlled blood glucose can be enrolled in this study.

For concomitant treatments and medications, the dose, frequency, route and date of administration should be recorded in the CRF. In case of AEs, the subjects should be closely observed, active symptomatic treatment should be given if necessary, and the medications used should be recorded and explained.

5.6.2 Other anti-tumor therapies

During the study treatment period, the use of other systemic anti-tumor therapies such as chemotherapy, molecular targeted therapy, immunotherapy, and biological therapy for the treatment of esophageal cancer is not allowed, including but not limited to modern Chinese medicine preparations approved by the NMPA with indications for esophageal cancer (refer to the relevant classifications and recommendations in Expert Consensus on the Use of Chinese Patent Medicines for Palliative Treatment of Tumors (2013)).

During the study treatment, the use of immunostimulants and immunomodulators is prohibited (including but not limited to interferons, interleukin-2, thymosin, thymalfasin, and immunocell therapy, etc.).

During the study treatment, any radiotherapy targeting tumor lesions is not allowed.

During the study treatment, participation in clinical studies of other drugs/devices is not allowed.

5.6.3 Glucocorticoids and immunosuppressants

Systemic glucocorticoids and immunosuppressants (e.g., TNF-α inhibitors) may weaken the immunological effects of immune checkpoint inhibitors. Long-term, systemic use of glucocorticoids for palliative or supportive care purposes is not allowed. Short-term use of glucocorticoids for non-autoimmune diseases (e.g., delayed allergic reactions caused by contact allergens) is allowed for individual subjects.

Emergency use of glucocorticoids, topical application, inhalation spray, eye drops, or local injection is allowed.

The use of systemic glucocorticoids at physiological replacement doses (≤ 10 mg/d of prednisone or equivalent) is allowed.

The use of prophylactic glucocorticoids to prevent allergic reactions (e.g., premediction before intravenous contrast administration) is allowed according to the drug instructions.

The use of glucocorticoids and immunosuppressants is permitted for the treatment of irAEs.

5.6.4 Surgery (non-anti-tumor purpose)

Any surgery planned to be performed during the study period should be justified and necessary. The time interval between surgery and administration of the study drug should be adequate for recovery of surgery wound and identification of the cause of unexplained bleeding.

It is recommended that the study drug should be discontinued within 1 week before surgery. Treatment resumption is based on the clinical assessment of wound healing and postoperative recovery.

6. Study Procedures

The utmost effort will be made to ensure that the required tests and procedures are carried out according to the protocol. However, unexpected situations beyond the investigator's control may occur from time to time, making it difficult to perform the tests and procedures. In such cases, the investigator must take all necessary measures to ensure the safety and interests of the subjects. When a required test cannot be conducted, the investigator needs to record the reasons.

6.1 Screening Period

First, it should be determined whether the subject has signed the ICF. The radiological examinations, pathological diagnoses, laboratory tests, etc., that are available within a specified time limit prior to randomization may be used as examinations at screening. Written informed consent must be obtained from subjects included in the study.

This study allows subjects who have previously failed screening to be screened again. The re-screened subjects must sign a new ICF and assigned a new subject number.

The time from when the subject signs the ICF to the first dose should not exceed 21 days. Subjects will be evaluated for final eligibility for the study within the specified time period given in the Schedule of Activities.

The examination items and procedures for the screening phase can be found in detail in the Schedule of Activities.

6.2 Treatment Period

The study treatment will be started within three working days after randomization.

If the scheduled visit prior to the first dose has been performed at screening and is performed within 7 days prior to the first dose, then there is no need for another test before the first dose. A ±3-day time window is allowed for visits in each cycle during the study.

Tumor assessment will be performed regularly using RECIST 1.1 criteria for radiological response evaluation. All subjects will undergo tumor radiological assessments at screening and have a tumor radiological assessment after Cycle 2 of neoadjuvant therapy upon entering the study. If surgery treatment is more than 6 weeks after the last dose of neoadjuvant therapy, the tumor radiological assessments will be repeated within 14 days prior to surgery. Tumor assessments should still be performed regularly after surgery (see details in Section 7.2.2).

When disease progression is suspected (e.g., worsening of clinical symptoms) and the subject's treatment has ended, an additional assessment should be conducted if it has not been completed in the previous 4 weeks. When there are clinical indications, the frequency of assessments can be increased according to the investigator's arrangement. After the initial assessment of radiological disease progression, if the subject is clinically stable, a follow-up radiological assessment is required after 4-6 weeks for confirmation. If the subject's clinical condition is unstable, there is no need for confirmation after 4-6 weeks.

Specific tests and examinations as well as procedures required for each visit are detailed in the Schedule of Activities.

It is required to record AE evaluation and concomitant medication/concomitant therapies at each visit.

6.3 End of Treatment and Safety Follow-up Period

The subject completes the end of treatment visit at the end of treatment.

At the end of the study treatment, if a subject has relevant test results within 14 days before the end of treatment, these tests need not be repeated; otherwise, the tests specified in the protocol should be conducted. Visit contents are detailed in the Schedle of Activities.

The definition of safety follow-up period: 90 days after the last dose of camrelizumab for the test group; 30 days after surgery for patients who have undergone surgery or 30 days after the last dose of study drug for patients who have not undergone surgery in the control group. Only SAEs considered related to the study drug are collected after the safety follow-up period. Subjects in the test group will be followed up every 30 days until 90 days after the last dose of camrelizumab, either through clinical visits or telephone interviews, to collect information on the subjects' survival status, AEs, concomitant medications, and concomitant therapies. Investigators may increase visits as needed for AE follow-up, with the aim of monitoring the resolution of AEs.

The investigator may increase visits as needed for AE follow-up, with the aim of monitoring the esolution of AEs.

6.4 Survival Follow-up

After discontinuation of study treatment, survival follow-up will be performed every 3 months, either through clinical visits or telephone interviews, to collect information on the subjects' survival (death date and cause of death) and information after the end of study treatment (including subsequent anti-tumor therapy and efficacy), until the endpoint of death, loss to follow-up, or termination of the study.

6.5 Tumor Progression/Recurrence Follow-up

Subjects who discontinue treatment for reasons other than radiological progression (such as toxicity) should be followed up for tumor progression and radiological examination should be performed at the end of treatment (unless it has been performed within 28 days), and thereafter should continue to undergo tumor assessments as planned until disease progression, loss to follow-up, death, withdrawal of informed consent, or termination of the study.

After the subjects discontinue treatment due to recurrence or initial progression, the investigator should assess or follow up the subjects every 3 months and record the assessment results.

6.6 Unscheduled Visits

Subjects may experience AEs during the study that require unscheduled visits, which should include follow-up on the following items:

- AEs;
- Concomitant medications/therapies;
- Relevant tests and examinations performed (including laboratory tests and radiological examination, if any).

6.7 Collection of Tissue Samples and Blood Samples

Subjects should agree to provide formalin-fixed paraffin-embedded (FFPE) tumor tissue blocks or at least 10 slides of unstained tumor specimen obtained prior to the first dose, and approximately 10 mL of whole blood samples will be collected at baseline.

7. Efficacy Evaluation

7.1 Efficacy Parameters

Primary efficacy endpoint:

- pCR assessed by Blinded Independent Review Committee (BIRC), defined as the proportion of subjects with no residual tumor in the primary tumor site (Mandard grade 1) and histologically negative lymph nodes;
- EFS assessed by the investigator according to RECIST 1.1, defined as the time from randomization to the occurrence of any of the following events, whichever comes first:
  - Radiographic tumor progression assessed by RECIST 1.1;
  - Tumor recurrence assessed by radiology or tissue biopsy, including local recurrence or distant metastasis (for subjects with no residual tumor after surgery);
  - Death due to any cause;

Note: A second primary malignancy, or radiological progression occurring during the neoadjuvant phase that does not affect the radical resection of esophageal cancer, is not considered an EFS event.

Secondary efficacy endpoints:

- MPR assessed by BIRC: The percentage of subjects with <10% residual tumor in the primary tumor site;
- R0 resection rate;
- Pathological stage after neoadjuvant therapy (ypTNM stage) based on the 8th edition of AJCC;
- OS: The time from randomization to death due to any cause;
- DFS: The time from the first day of no disease (i.e., the date of surgery) to local or distant recurrence, or death due to any cause, whichever occurs first;

7.2 Efficacy Assessment

7.2.1 Pathological assessment

Each study institution should designate a physician engaged in the pathological assessment of esophageal cancer, preferably a senior attending physician with 5 or more years of experience. The pathological response should be assessed according to the Mandard criteria, and the radicality and ypN staging should be assessed according to the 8th edition of AJCC.

Pathological response evaluation: Based on the surgically resected primary tumor foci, the assessment site should include all primary tumor foci seen on the radiological examination performed before the neoadjuvant therapy, and slides that may contain residual tumor cells will be selected for assessment. Viable tumor cells are defined as cells of proliferative potential, and the tumor regression grading (TRG) according to the Mandard criteria [15] is described as follows:

TRG1	Complete tumor regression
TRG2	Fibrosis of most tumor beds, small foci or residual individual tumor cells
TRG3	Areas of fibrosis greater than areas of residual tumor cells

TRG4	Area of fibrosis less than the area of residue tumor cells
TRG5	No tumor regression

In this study, all pathological slides will be independently reviewed by the BIRC. Each study site will submit postoperative pathological slides to the independent pathology review institution during the study or upon the sponsor's request. Detailed rules and guidelines for the BIRC's pathological response assessment will be described in a separate operation manual.

The preservation and processing of surgical specimens, and detailed rules and guidelines for the assessment of pathological reseponse will be described in a separate central operation manual.

7.2.2 Radiological assessment

Efficacy indicators such as EFS, DFS, etc., are assessed using radiological examinations according to the RECIST 1.1 criteria[10].

High-resolution CT with oral/intravenous contrast agents is the preferred radiological method for tumor radiological assessment. Unless otherwise specified in this section, tumor radiological assessment refers to the assessment using contrast-enhanced CT (CT scan slice thickness ≤ 5 mm). If the subject is known to be allergic to contrast agents, the contrast-enhanced radiological assessment should be performed according to appropriate prophylactic standards (if possible), or contrast-enhanced MRI should be adopted. It is recommended to add neck ultrasound for the diagnosis and differential diagnosis of metastatic lesions such as cervical lymph nodes. If the use of contrast agents is strictly prohibited, a non-contrast CT scan of the chest may be used, and an ultrasound of the neck and an MRI scan of the abdomen (plain + enhanced) should be performed. PET-CT may be used for tumor assessment, but the technical parameters and imaging quality of its CT component must meet the standards for radiological assessment using CT.

Radiological examinations during the screening period should be completed within 21 days before randomization, with enhanced CT or MRI of the neck/chest/abdomen, and endoscopic ultrasound (EUS) for tumor assessment and clinical staging. When possible, PET-CT is also recommended to exclude metastatic lesions. During the screening period, all measurable and evaluable lesions should be assessed and recorded, and radiological assessment of the tumor should be performed after completion of Cycle 2 during neoadjuvant therapy according to the Schedule of Activities, including the neck, chest, abdomen, or other suspected disease sites. Baseline assessment and post-treatment response assessment should use the same method (imaging technical parameters and imaging quality should be consistent with the screening period). Investigators must promptly review the results of the scans after Cycle 2 to assess response, determine whether the patient should undergo surgery (for patients with disease progression but still suitable for surgical treatment) and resectability, or other standard of care (for patients with disease progression and no longer suitable for surgical treatment).

Surgery should be performed within 4-6 weeks after the last dose of neoadjuvant therapy. If surgery is planned for 6 weeks after the last study treatment, a CT scan should be performed again within 14 days prior to the planned surgery.

For subjects who have completed surgery, a baseline scan should be performed at 4 weeks (±7 days) after surgery and before the start of adjuvant therapy in Groups A and B, followed by a radiological assessment of tumor every 12 weeks (±14 days) starting from the postoperative baseline Scan, and every 24 weeks (±28 days) starting from the third year. The frequency of radiological monitoring can be increased when there are clinical indications.

For subjects who have not completed radical surgery (including those who have not undergone surgery for various reasons or are found to be inoperable during surgery), radiological assessments of tumor will be performed every 12 weeks (±14 days) from the post-neoadjuvant radiological assessment, and every 24 weeks (±28 days) starting from the third year. The frequency of radiological monitoring can be increased when there are clinical indications.

Tumor evaluations should be conducted for all subjects at the frequency specified in the protocol, regardless of interruptions or delays in medication. Response should be assessed by the investigator according to RECIST 1.1 criteria. At each evaluation, tumor measurements should be performed by the same investigator or radiologist whenever possible to ensure consistency between visits.

If the subject's clinical symptoms are stable at the time of the first radiologically confirmed disease progression of tumor on treatment with camrelizumab, it is recommended to perform another radiological examination after an interval of 4-6 weeks for confirmation. If disease progression is confirmed, tumor assessments will be performed at the originally planned frequency. If it is not confirmedand the investigator judges that the subject benefits from continuing camrelizumab treatment, the subsequent treatment period should follow the tumor assessment frequency as originally specified in the protocol. If clinical symptoms are unstable, no further radiological confirmation is needed. If disease progression is observed in the subject's next tumor assessment, and the investigator determines that the subject can no longer benefit from continuing treatment, they should be withdrawn from study treatment;

When clinically indicated, the frequency of assessments can be increased according to the investigator's arrangement.

Once clinical progression occurs, a physical examination and immediate radiological examination are required at any time, rather than waiting for the next scheduled radiological examination. If this unscheduled radiological examination does not confirm clinical progression as defined by RECIST v1.1, the next examination should still be carried out on the date originally planned, unless the interval between the next scheduled examination and this examination is less than 14 days.

For subjects who discontinue treatment for reasons other than radiological progression (such as toxicity), an radiological examination should be conducted at the end of treatment (unless an radiological examination has been done within the last 28 days), and subsequent tumor assessments should continue as much as possible at the above frequency, following the previous schedule until disease progression, recurrence, loss to follow-up, death, withdrawal of study consent, or termination of the study (whichever comes first).

8. Safety Evaluation

8.1 Safety Parameters

- AEs: All SAEs/AEs that occur during the study, including symptoms and signs at screening, whether related to the study drug or not, must be recorded in the CRF.
- Surgical complications, perioperative mortality: Surgical complications are recorded within 30 days after surgery or during the hospital stay after surgery, and deaths are recorded within 90 days after surgery.

8.2 Safety Assessment Criteria

Each subject will be assessed by the investigator or a qualified person designated by the investigator, with the aim to evaluate possible new or worsening AEsas specified in the

Schedule of Activities, and more frequently if clinically indicated. The assessment of AEs includes type, incidence, severity, onset and end time, outcome, and relationship to treatment/drug, etc. During the study and follow-up period, AEs will be graded and recorded according to NCT-CTCAE v5.0.

If an adverse event is assessed by the investigator as a surgical complication, its severity should also be graded and recorded according to the Clavien-Dindo (CD) criteria.

8.3 Laboratory Safety Evaluation

- **Hematology:** total blood cell differential count + five-part differential, red blood cell count, haemoglobin, platelet count;
- **Blood chemistry:** total bilirubin (TBIL), direct bilirubin (DBIL), alanine aminotransferase (ALT), aspartate aminotransferase (AST), alkaline phosphatase, total protein, albumin, blood urea nitrogen (BUN), creatinine (Cr), endogenous creatinine clearance rate (Ccr), blood glucose, amylase, potassium (K), sodium (Na), chlorine (Cl), calcium (Ca), phosphorus (P);
- **Urinalysis:** urine protein, occult blood, red blood cells, white blood cells;
- **Thyroid function:** serum free thyroxine (FT3), free thyroxine (FT4), and serum thyroid-stimulating hormone (TSH); triiodothyronine (T3) and thyroxine (T4) can be used as alternatives;
- **Muscle enzyme spectrum:** creatine kinase MB isoenzyme (CK-MB), cardiac troponin I (cTnI), lactate dehydrogenase (LDH);
- **Fecal occult blood;**
- **Coagulation function:** international normalised ratio (INR), activated partial thromboplastin time (APTT), prothrombin time (PT), fibrin (FIB);
- **Virology:** HBsAg (if positive, HBV-DNA testing is required), HBsAb, HBeAg, HBeAb, HBcAb, HCV-Ab (if positive, HCV-RNA testing is required), HIV-Ab
- **Pregnancy test:** serum or urine pregnancy;
- **12-lead ECG:** heart rate, QT interval, and P-R interval should be indicated;
- **Echocardiogram:** it must include left ventricular ejection fraction (LVEF, %)

The timing of each test/examination is detailed in the Schedule of Activities.

8.4 Others

Vital signs include body temperature, blood pressure, pulse, and respiratory rate.

Physical examination includes general condition, head and face, eyes, ENT (ear, nose, throat), oral cavity, skin, lymph nodes, respiratory system, cardiovascular system, gastrointestinal system, genitourinary system, musculoskeletal system, nervous system, mental status, and others.

The timing of each examination is detailed in the Schedule of Activities.

9. Patient-reported Outcomes

Two self-assessment questionnaires, EORTC QLQ-C30 and OES18, are used in this study. All subjects at screening need to complete them after signing the ICF and before the first dose; during the treatment period, they should be completed within 14 days before surgery, 4 weeks

after surgery (test groups A and B need to complete them before receiving postoperative camrelizumab treatment), then every 12 weeks (±14 days), and every 24 weeks (±28 days) starting from the third year, at end of treatment/withdrawal visit, and at the end of the safety follow-up period.

The sponsor will provide training for personnel involved in managing the questionnaires (such as the principal investigator, clinical research associate) to ensure that subjects fill out the questionnaires as completely and accurately as possible, and conscientiously collect data as required. It is very important to explain the significance and relevance of the data in detail to the participating subjects.

Each study site must designate specific personnel (such as study nurses, study coordinators) to be responsible for conducting surveys, and if possible, assign a support staff to substitute in their absence. Before initiating any other study activities or discussions on study drug treatments or disease progression, and before any contact with investigators, study sites should hand out paper questionnaires to subjects, advising completion within 30 minutes to avoid bias in responses. Study sites should strive to ensure that a fully completed questionnaire is obtained from each subject at each planned time point to prevent any lag in clinical assessments.

As it is a self-assessment report, subjects must complete the questionnaire themselves without assistance from friends, family, or clinical staff; nor is it permitted for any personnel to help interpret the questionnaire. Subjects are required to fill out the questionnaire as completely and accurately as possible. If local regulations have confidentiality requirements regarding questionnaire completion (e.g., the completed questionnaires should not be seen by clinical staff), the sites should take appropriate measures to ensure compliance as much as possible. However, if patients are unable to read the questionnaire (e.g., blindness or illiteracy), it may be read and responses recorded by trained clinical staff.

The completed questionnaires will be returned to the designee responsibe for patient-reported outcomes (PROs), who will check for completeness of the form. There is only one answer to each question.

9.1 EORTC QLQ-C30

EORTC QLQ-C30, the most widely used cancer-specific HRQoL assessment tool, contains 30 items and measuring 5 functional dimensions (physical, role, emotional, cognitive, and social functions), 3 symptom items (fatigue, nausea/vomiting, and pain), 6 single items (dyspnoea, sleep disorder, loss of appetite, constipation, diarrhoea, and financial impact), as well as an overall health and quality of life (QoL) scale [Aaronson, N. K., et al 1993]. Therefore, the exploratory objective is to assess the mean change in overall health status/QoL score relative to baseline using the EORTC QLQ-C30.

9.2 EORTC QLQ-OES18

EORTC QLQ-OES18 is a developed and validated disease-specific questionnaire for measuring esophageal cancer-specific indicators. It is one of several disease-specific modules developed by the EORTC Quality of Life Working Group, designed for clinical studies and used in conjunction with the EORTC QLQ-C30 to assess disease-specific treatment indicators. It contains 18 items, 6 of which involve swallowing saliva, choking, dry mouth, taste, cough, and speaking; additionally, it includes 12 items divided into 4 subscales: dysphagia (3 items), eating (4 items), reflux (2 items), and pain (3 items).

10. Adverse Event Reporting

10.1 Adverse Events

10.1.1 Definition of AE

An AE is defined as any untoward medical occurrence in a clinical study subject after administration of a drug, but does not necessarily have a causal relationship with the study treatment. AEs can be any unfavorable and unexpected symptoms, signs, laboratory abnormalities, or diseases, including the following situations::

1) The pre-existing (before entering the clinical study) medical conditions/diseases, which only worsen after the start of study drug (including worsening in symptoms, signs and laboratory abnormalities);
2) Any new AEs: any new adverse medical conditions (including symptoms, signs, newly diagnosed diseases);
3) Abnormal clinically significant laboratory findings.

Any invasive procedures (such as surgery) and non-invasive procedures for diagnosis or treatment should not be reported as an AE. However, if the medical condition leading to the procedure meets the definition of an AE, it should be reported as an AE. For example, acute appendicitis occurring during the AE reporting period should be reported as an AE, while the appendectomy performed as a result should be documented as the treatment of the AE.

The study personnel should record all AEs occurring in subjects in detail, including the AE name, onset and end dates, severity (graded according to NCI CTCAE v5.0), relationship with the study drug, duration, action taken with the study drug due to the AE, the outcome of the AE, whether it is a serious AE or not, etc.

10.1.2 Criteria for determining the severity of AEs

The severity of AEs will be determined in reference to the NCI-CTCAE v5.0 grading criteria. For AEs not listed in the NCI-CTCAE v5.0, please refer to the following criteria:

Grade	Clinical Description of Severity
1	Mild; with no symptoms or minor clinical symptoms; only clinical or laboratory abnormalities; no treatment required;
2	Moderate; requiring minor, local, or non-invasive treatment; limitations in age-appropriate instrumental activities of daily living (ADL); instrumental ADL refer to cooking, shopping, making phone calls, and counting money, etc.;
3	Severe or medically significant but not immediately life-threatening; hospitalization or prolongation of hospitalization indicated; disabling; limiting self care ADL. Self-care ADL refer to bathing, dressing and undressing, feeding self, using the toilet, taking medications, and not bedridden;
4	Life-threatening; urgent intervention indicated;
5	Leading to death;

Note that the seriousness and severity of AEs should be differentiated. For example, “severe headache” may be severe in seriousness but cannot be reported as a serious adverse event (SAE) unless it meets the criteria for an SAE.

Clavien-Dindo (CD) grading criteria for postoperative complications:

Grade	Clinical Description of Severity
Grade I	Any deviation from the normal postoperative course without the need for pharmacological

	treatment or surgical, endoscopic and radiological interventions<br>Allowed therapeutic regimens are: drugs as antiemetics, antipyretics, analgetics, diuretics and electrolytes and physiotherapy. This grade also includes wound infections opened at the bedside.
Grade II	Requiring pharmacological treatment with drugs other than such allowed for grade I complications.<br>Blood transfusionsand total parenteral nutritionare also included.
Grade IIIa	Requiring surgical, endoscopic or radiological intervention, intervention not under general anesthesia
Grade IIIb	Requiring surgical, endoscopic or radiological intervention, intervention under general anesthesia
Grade IVa	Life-threatening complication (including CNS complications)* requiring IC/ICU-management, single organ dysfunction (including dialysis)
Grade IVb	Life-threatening complication (including CNS complications)* requiring IC/ICU-management, multiorgandysfunction
Grade V	Death of a patient;

10.1.3 Determination of relationship between AEs and the study drug

The investigator should determine whether there is a reasonable possibility of the study drug to cause or contribute to the AE by comprehensive assessment, including whether there is a reasonable temporal sequence between the occurrence of the AE and the administration of the study drug, the characteristics of the study drug, the toxicological and pharmacological effects of the study drug, the use of concomitant medications, the subject's underlying disease, medical history, family history, and dechallenge and rechallenge reactions, etc. The possible causality between the AE and the study drug should be assessed as "definitely related, possibly related, unlikely related, not related, and unable to determine" using a five-level classification method.

10.2 Serious Adverse Events

10.2.1 Definition of SAE

An SAE is an AE that meets one or more of the following criteria during the course of a clinical study:

- Events leading to death;
- Life-threatening (the term "life-threatening" refers to a situation in which the subject has risk of death at the time of the event/reaction, rather than assuming a possible death if the condition worsens);
- Events requiring hospitalization or prolongation of hospitalization;
- Events leading to permanent or serious disability/loss of function;
- Congenital anomalies or birth defects;
- Other medically significant events, which are events/reactions that do not immediately threaten life or lead to death or hospitalization, but based on reasonable medical and scientific judgment, may harm the subject or may require intervention [e.g., medication or surgery] to prevent the serious consequences defined above.

10.2.2 Hospitalization

AEs in the clinical study that lead to hospitalization (even if less than 24 hours) or prolongation of existing hospitalization should be considered as SAEs.

However, hospitalization or prolongation of hospitalization for the following reasons should

not be reported as an SAE:

- Rehabilitation facility
- Sanatorium
- Routine emergency room treatment (less than 24 hours)
- Day surgery (such as outpatient/day/ambulatory surgery)
- Social reasons (medical insurance reimbursement, etc.)
- Hospitalization or prolongation of hospitalization due to any causes other than AEs will not be considered as an SAE. This includes, but is not limited to, the following:
  - Hospitalization for treatment of a pre-existing disease, which is not associated with the new AE and does not involve worsening of a pre-existing disease (e.g., hospitalization to check laboratory abnormalities that occurred prior to the study and are still present);
  - Hospitalization for administrative reasons (e.g., annual routine physical examination);
  - Hospitalization during the clinical study as specified in the study protocol (e.g., procedures required by the study protocol);
  - Elective hospitalization unrelated to an AE (e.g., elective cosmetic surgery);
  - Pre-planned treatments or surgical procedures (they should be documented in the overall study protocol and/or individual subject baseline data);
  - Hospitalization for blood product use only.

10.3 Collection/Follow-up of Adverse Events and Reporting of Serious Adverse Events

10.3.1 Collection and follow-up of AEs

AE/SAE information will be collected from the time the subject signs the ICF until the end of the safety follow-up period (the end of the safety follow-up period is defined as 90 days after the last dose of camrelizumab for the study group, 30 days after surgery for patients who have undergone surgery, or 30 days after the last dose of study drug for patients who have not undergone surgery, or screening failure).

If a subject starts a new anti-tumor therapy before the end of the safety follow-up period, the collection period for non-fatal AEs/SAEs suspected to be not related to the study drug will end at the start of new anti-tumor therapy.

If death occurs during the safety follow-up period, it must be reported as an SAE regardless of whether the subject has received other treatments.

After the safety follow-up period, only study drug-related SAEs will be reported by the investigator.

At each visit, the investigator should inquire about any AE/SAE that occurred after the last visit. Follow-up information should be provided promptly according to the queries received.

All AEs/SAEs should be followed up until symptoms subside, or clinically relevant changes in laboratory values return to baseline and/or ≤ Grade 1, or a reasonable explanation is obtained

(such as loss to follow-up, death), or until the event is finally confirmed to be not related to the study drug or study procedure at the end of the safety follow-up period.

10.3.2 SAE reporting procedure

In the event of an SAE, whether it is an initial report or a follow-up report, the investigator must immediately fill out the Serious Adverse Event Report Form, sign and date it, and report to the relevant department within 24 hours of becoming aware. **If an SAE occurs after the start of treatment with camrelizumab or albumin-bound paclitaxel, it should also be reported to the Hengrui Drug Safety Department** (hengrui_drug_safety@hengrui.com).

All SAEs should be recorded in detail, including symptoms, severity (refer to NCI-CTCAE version 5.0), relationship to each study drug, onset time, treatment time, action taken with each study drug due to the SAE, follow-up time and mode, and outcome, etc. If the investigator believes that a certain SAE is not related to the study drug but potentially related to study conditions (e.g., discontinuation of previous treatment, or complications during the study), this relationship should be detailed in the narrative section of the SAE report form. If the severity of an ongoing SAE or its relationship to the study drug is changed, a follow-up report should be submitted immediately. If the investigator believes that previously reported SAE information was misreported, then correction, revocation or downgrading instructions may be made through a follow-up report, which should be submitted in accordance with the SAE reporting procedures.

10.4 Pregnancy Reporting

If a female subject becomes pregnant during the clinical study, the subject should immediately discontinue the study drug and withdraw from the study; if the partner of a male subject becomes pregnant during the clinical study, the subject will continue the clinical study.

During the clinical study, the investigator should fill out the Hengrui Clinical Trial Pregnancy Report/Follow-up Form within 24 hours of becoming aware of a pregnancy event and report it to the relevant department; if the pregnancy occurs after the start of using camrelizumab or albumin-bound paclitaxel, it should also be reported to the Hengrui Drug Safety Department (hengrui_drug_safety@hengrui.com).

The investigator should follow up the pregnancy event to the final outcome (including any premature termination of pregnancy or delivery), follow up the delivery until one month after the mother gives birth, and report the result to the relevant institutions and Hengrui Drug Safety Department (hengrui_drug_safety@hengrui.com).

If the outcome of the pregnancy is ectopic pregnancy, spontaneous abortion, intrauterine fetal death, neonatal death, or congenital anomaly, etc., it should be considered an SAE and reported within the time limit required for SAE.

If a subject becomes pregnant and an SAE occurs during the AE/SAE information collection period, the Serious Adverse Event Report Form must also be filled out, and the SAE reporting procedure must be followed.

10.5 Non-serious Adverse Events/Reactions

The non-serious adverse events/reactions of the investigational drug will be provided to Hengrui through the final clinical study report.

10.6 Disease Progression and Death Reporting

Disease progression is defined as the worsening of the subject's condition caused by the indication of the study. It includes radiological progression and the progression of clinical

symptoms and signs. A new lesion of the primary tumor or progression of an existing lesion will be considered disease progression.

Events that are life-threatening, requiring hospitalization or prolongation of existing hospitalization, or leading to permanent or significant disability/incapacity due to symptoms and signs of disease progression will not be reported as SAEs. If there is any uncertainty as to whether an SAE is caused by disease progression, it should be reported as an SAE.

In the study population of this study, “disease progression” is an expected occurrence and should not be reported as an AE term. When disease progression occurs, the event used to confirm disease progression should be reported as an AE. For example, if a subject has epilepsy that is determined to be related to brain metastasis, the AE term should be recorded as “epilepsy”, rather than ”disease progression” or “brain metastasis”.

During the safety follow-up period, deaths assessed by the investigator as possibly caused by symptoms and signs of disease progression should be reported as SAEs.

However, the term “death” should not be used as an SAE term, but rather as the outcome of the event, and the adverse event that caused or led to death should be recorded as an SAE term. If the cause of death cannot be determined at the time of reporting, the SAE term should be recorded as “death of unknown cause”.

10.7 Immune-related Adverse Events

irAEs refer to specific events in which subjects need to receive immunosuppressive drugs for treatment, including pneumonia, diarrhea/colitis, hepatitis, nephritis/renal dysfunction, rash, endocrine disorders, etc. Endocrine disorders (hypothyroidism/thyroiditis, hyperthyroidism, hypophysitis, diabetes mellitus, adrenal insufficiency) are usually exceptions, although they are immune-related events, they are usually unrelated to treatment and can be managed without immunosuppressive intervention.

10.8 Hepatic Function Abnormal

If AST and/or ALT levels are abnormalalong with the abnormal increase of total bilirubin (TBIL) level, and the following abnormalities (1) (2) (3) are present without any other causes for the abnormalities, such cases should be **considered significant medical events and reported according to the SAE procedure**.

Abnormalities	Judgment Criteria
(1) ALT or AST abnormal	Normal at baseline: ALT or AST ≥ 3× upper limit of normal (ULN) during treatment;<br>Abnormal at baseline: ALT or AST ≥ 2× baseline level, and value ≥ 3× ULN; or value ≥ 8 × ULN during treatment;
(2) TBIL abnormal	Normal at baseline: TBIL > 2×ULN during treatment;<br>Abnormal at baseline: TBIL increase ≥ 1×ULN or value > 3×ULN during treatment;
(3) No hemolysis, and alkaline phosphatase < 2×ULN (or no information available)	

ULN (upper limit of normal)

If a subject has abnormal levels of AST and/or ALT with an abnormal increase in TBIL level during the safety follow-up period, it is recommended to return to the study site as soon as possible (preferably within 48 hours) for assessment and confirmation after learning the abnormal results.

11. Data Analysis/Statistical Methods

Detailed statistical analysis of this study will be included in the Statistical Analysis Plan (SAP), which will be finalized before database lock. The SAP will provide the complete content of the analysis, results presentation, and other relevant information, and will be kept by the investigator. Appropriate modifications can be made in the SAP for the plan outlined in the protocol. However, any significant revisions to the definitions and analysis of the primary study endpoints need to be reflected in the amended protocol.

11.1 Sample Size

The study's co-primary endpoints are pCR and EFS, and the final analysis of the study will be performed when approximately 228 EFS events are observed, with a planned sample size of about 390 subjects.

(1) pCR: Assuming that the pCR of test group A is 30%, the pCR of test group B is 25%, and the pCR of control group C is 9%, with a randomization ratio of 1:1:1, and α set at one-sided 0.005, 111 subjects in each group are expected to provide at least 93% power to detect that test group A is superior to control group C, and can provide at least 75% power to detect that test group B is superior to control group C. Considering a 15% dropout rate, each group will need 130 subjects.

(2) EFS: Assuming the median EFS in the control group is 30 months, the hazard ratio (HR) for the test group (A+B) compared to the control group is 0.67, with an initial α assignment set at one-sided 0.02, the efficacy interim analysis of EFS is planned when 70% of events is observed;
- If the randomization ratio is 2:1 (test group A+B vs. control group C), then 228 events (141 in the test group, 87 in the control group) are required to provide at least 80% power to detect the superiority of the test group over the control group, with an enrollment period of 36 months, a total study duration of 84 months, and a dropout rate of 15%, so approximately 390 subjects are needed for the three groups.
- If the randomization ratio is 1:1 (test group A vs. control group C or test group B vs. control group C), 158 events (71 in the test group, 87 in the control group) can be observed in the above sample size, to provide at least 67% power to detect the superiority of test group B or test group A over the control group.

11.2 Statistical Hypotheses

This study is designed for superiority, and is statistically tested for the dual primary endpoints pCR and EFS using the multiplicity test. The multiplicity strategy is detailed in Section 11.4.5. This study includes two primary hypotheses and three secondary hypotheses.
The two primary hypotheses are:
1) pCR: Test group A vs. control group
H_{01} (null hypothesis): The pCR rate of test group A is lower than or equal to that of the control group.
H_{11} (alternative hypothesis): The pCR rate of test group A is higher than that of the control group.
2) EFS: Test group A + B vs. control group
H_{02} (null hypothesis): The EFS of test group A+B is worse than or equal to that of the control group.
H_{12} (alternative hypothesis): The EFS of test group A+B is superior to that of the control group.
The three secondary hypotheses are:
3) pCR: Test group B vs. control group

H_{03} (null hypothesis): The pCR rate of test group B is lower than or equal to that of the control group.
H_{13} (alternative hypothesis): The pCR rate of test group B is higher than that of the control group.
4) EFS: Test group A vs. control group
H_{04} (null hypothesis): The EFS of test group A is worse than or equal to that of the control group.
H_{14} (alternative hypothesis): The EFS of test group A is superior to that of the control group.
5) EFS: Test group B vs. control group
H_{05} (null hypothesis): The EFS of test group B is worse than or equal to that of the control group.
H_{15} (alternative hypothesis): The EFS of test group B is superior to that of the control group.

The Cochran-Mantel-Haenszel (CMH) chi-square test is used for intergroup comparison of pCR, and the stratified Logrank test is used for intergroup comparison of EFS. Stratification factors include clinical stage (Stage I/II vs. Stage III vs. Stage IVa). The sequence of tests is detailed in Section 11.4.5 on multiplicity, and the specific process of hypothesis testing is detailed in the statistical analysis plan (SAP).

11.3 Analysis Sets

- Intention to Treat (ITT): According to the ITT principle, it includes all subjects who are randomized. The ITT set is the primary analysis set for the efficacy analysis of this study.
- Per-Protocol Set (PPS): It is a subset of the ITT set that is more compliant with the protocol; subjects with significant protocol deviations judged to have a significant impact on the primary efficacy analysis will be excluded from the PPS.
- Safety Set (SS): It will include subjects who have received at least one dose of study drug. Analysis will be based on the actual treatment group received.
- Surgery Analysis Set: It will include all subjects who have undergone the prescribed surgery. This analysis set is primarily used for the assessment of surgery safety.

11.4 Statistical Analysis Methods

11.4.1 General analysis

For continuous data, statistics such as number, mean, standard deviation, median, minimum, and maximum values will be summarized; for categorical data, statistics such as frequency and percentage will be summarized; for time-to-event data, the Kaplan-Meier method will be used to estimate the survival function and the median time to event occurrence, and survival curves will be plotted.

11.4.2 Efficacy analysis

11.4.2.1 Primary efficacy endpoints

The primary efficacy endpoints of this study are pCR and EFS.

pCR:

Based on the ITT analysis set, the differences in pCR between test group A and the control group, test group B and the control group, along with the 95% confidence intervals and p-values, will be compared using a stratified Cochran-Mantel Haenszel (CMH) test. An unstratified sensitivity analysis based on the ITT analysis set and a sensitivity analysis based on the surgery analysis set will be conducted.

EFS:

Analyses will be performed based on the ITT Analysis Set.The Kaplan-Meier method will be used to estimate the survival distribution for each treatment group and the combined A+B group, and survival curves will be plotted. The Brookmeyer Crowley method will be used to estimate the two-sided 95% confidence interval for median EFS. The differences in EFS between the test groups and the control group will be compared using a stratified log-rank test that considers stratification factors [redacted] The hazard ratio (HR) for the test groups compared to the control group and its 95% confidence interval will be estimated using a Cox proportional hazards model that considers stratification factors. Additionally, the number and percentage of subjects with events, and the number and percentage of subjects censored in each treatment group and the combined A+B group will be calculated, and the event types and reasons for censoring will be further categorized. Censoring rules will be detailed in the SAP. An unstratified sensitivity analysis based on the ITT analysis set and a sensitivity analysis based on the PPS analysis set will be conducted.

11.4.2.2 Secondary efficacy endpoints

For the time-to-event data among secondary endpoints, DFS and OS are analyzed using the same method as EFS, but the intergroup survival differences are only subjected to informal statistical hypothesis testing, and nominal p-values can be reported if needed; for the grade data among secondary endpoints, such as post-surgery pathological stage (ypTNM stage), the distribution of stage before and after medication will be described; for the binary data among secondary endpoints, the R0 resection rate, the MPR, etc. are analyzed using the same method as the primary efficacy endpoint pCR, but the inter-group differences are only subjected to informal statistical hypothesis testing, and nominal p-values can be reported if needed.

11.4.3 Safety analysis

Safety analysis will be based on the Safety Set and will include descriptive statistical summaries of the following data (but not limited to):

- Summary of AEs (all cause and treatment-related);
- Incidence and severity of AEs (all cause and treatment-related);
- Detailed summary of SAEs;
- Analysis of adverse event relatedness;
- Descriptive analysis of laboratory indicators, vital signs, and ECG data.
- Assessment of surgery complications (including 30-day and 90-day mortality).

AEs will be coded using the Medical Dictionary for Regulatory Activities (MedDRA). All treatment-emergent AEs, drug-related AEs, SAEs, and drug-related SAEs will be tabulated by

NCI-CTCAE Version 5.0 criteria using the worst grade. Laboratory test parameters during the study will be summarized according to the NCI-CTCAE Version 5.0 criteria, using the worst grade.

11.4.4 Interim Analysis

An interim efficacy analysis of EFS is planned for this study when 70% of EFS events (160 cases) have been collected (test group A+B vs. control group). There are two scenarios:

[redacted]

	[redacted]	[redacted]	[redacted]
[redacted]	[redacted]	[redacted]	[redacted]
[redacted]	[redacted]	[redacted]	[redacted]

[redacted]

	[redacted]	[redacted]	[redacted]
[redacted]	[redacted]	[redacted]	[redacted]
[redacted]	[redacted]	[redacted]	[redacted]

[redacted]

The final analysis of pCR and the interim analysis of EFS will be performed by an independent statistical team, and their results will be evaluated by an independent data monitoring committee (iDMC). For a more detailed plan of the final analysis of pCR and the interim analysis of EFS, please refer to the SAP and the iDMC charter.

11.4.5 Multiplicity

The sequential strategy will be applied to the two primary hypotheses H_{01}, H_{02} and three secondary hypotheses H_{03}, H_{04}, H_{05} of this study. Please refer to Section 11.2 for details of the

statistical hypotheses.

The overall type I error (α) for the two primary hypotheses is controlled at 2.5% (one-sided), with 0.5% assigned to the pCR hypothesis for test group A vs. control group, and 2% assigned to the statistical hypothesis test involving EFS.

α will be assigned and passed among multiple hypotheses using the graphical approach of Maurer and Bretz[11]. According to this approach, the study hypotheses may be tested more than once, and when a primary hypothesis is rejected, the α assigned to that hypothesis can be passed on to another hypothesis test. The following figure represents each hypothesis and its initial one-sided α assignment, with the circles above the lines connecting the hypotheses representing the weight of α reassigned to other hypotheses.

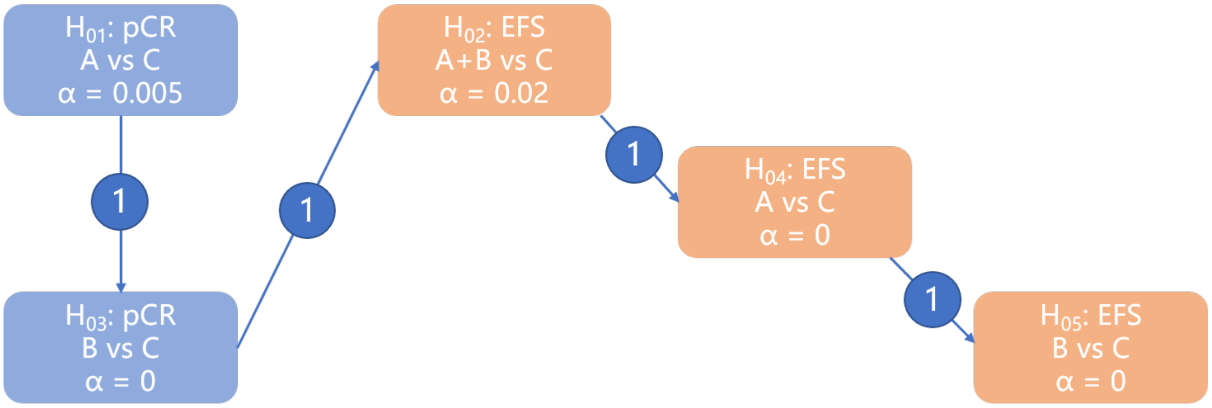

12. Data Management

12.1 Study Data Management

Investigators must retain all study process records and original documents for the longest period required by relevant regulations and guidelines, or procedures of the study site, whichever is longer. Before destroying any records related to the study, investigators must first contact the sponsor.

When study records are no longer needed, the sponsor will notify the investigators at each site.

If an investigator withdraws from the study (e.g., changing positions or retirement), the records should be transferred to a recognized designated person (e.g., another investigator). The sponsor must be notified of such a transfer.

12.1.1 Electronic Case Report Forms

Data managers will establish a study data collection system and database according to the study protocol and provide it online before subject enrollment. Before use, all EDC users must receive adequate training and fill out training records and account application forms to obtain the corresponding system login accounts.

Data in the eCRF should come from original documents such as study medical records and laboratory test reports and should be consistent with the original documents. Any observations and test results in the study should be filled in the eCRF in a timely, correct, complete, clear, standardized, and truthful manner, and should not be arbitrarily changed.

If necessary, when making data corrections in the eCRF, the reason for the data modification should be filled in according to the system prompts. The system's edit check program will check the integrity and logic of clinical study data entered into the EDC system and issue prompts for problematic data, allowing investigators or data entry personnel to modify or explain the

problematic data, and if necessary, queries can be issued multiple times until the problematic data is resolved.

Investigators should promptly complete, review, and submit the eCRF after each subject's visit, and timely respond to queries from study monitors, data managers, and medical reviewers. After data cleaning is completed, investigators should sign the completed eCRF for confirmation.

After the CRA's SDV is finished, data management personnel and medical staff will perform the final quality control on all data in the database, summarize all protocol deviations and violations that occurred during the study, and convene a data review meeting before statistical analysis. Decisions made at the data review meeting must be documented.

After all data have been reviewed and approved, the database will be locked upon confirmation by the investigator and statistician. After locking, the data files cannot be altered. After the database is locked, the statistician will apply to the sponsor for statistical analysis of the data. The locked data should be properly preserved for future reference.

After the study is completed, the EDC system should generate a PDF format of the subject's eCRF and save it on a non-rewritable CD-ROM, which will be handed over to each institution for archiving.

The preservation and management of study materials must be conducted in accordance with relevant laws, regulations, and GCP requirements, and the investigator should notify the sponsor in advance before destroying any documents or materials related to the study.

12.2 Data Monitoring

Hengrui's authorized representatives (study monitors, auditors, etc.) must be allowed to regularly visit all study sites to evaluate the quality of data and whether the study is complete and reliable. They will review the study records on site against the original documents, discuss the implementation of the study with the investigator, and confirm the study facilities still meet relevant requirements. The monitoring will focus on whether the study protocol is followed; whether all CRFs are filled out correctly and completely, and whether they are consistent with original documents such as medical records and laboratory test reports, and whether there are any errors or omissions in the data. Study monitors need to repeatedly verify the contents of the eCRF against the original documents to ensure the consistency of the data in the eCRF with the original data, a process also known as Source Data Verification (SDV).

Monitors will review the study data in the EDC database for completeness, consistency, and accuracy according to the monitoring plan, and discuss any problematic data with the study personnel, who will supplement or correct the data if necessary. They should ensure the consistency of the data in the eCRF with the original data.

12.3 Protocol Deviations

A protocol deviation refers to any practice that does not comply with the clinical study protocol, GCP, SOP, or medical operation procedures (MOP). Such non-compliance may occur in subjects, investigators, or other study personnel. If a deviation occurs, the study site should be prepared with corresponding corrective measures and be able to implement them immediately.

The study site has the responsibility to maintain continuous vigilance, promptly complete the identification, reporting, and handling of protocol deviations. All deviations must be recorded in the study's original documents and submitted to the local ethics committee according to local ethical regulations.

13. Raw Data and Original Documents

In accordance with ICH E6, relevant regulations, and the study institution's requirements for the protection of subject personal information, each study site must properly keep treatment and research records related to this study. As part of the sponsorship or participation in the study by Hengrui, each study site should allow Hengrui or its authorized representatives and regulatory authorities to inspect (and, if legally permitted, copy) clinical records for quality review, auditing, safety assessment, research progress evaluation, and data validity assessment. This section should also specify who has the right to access these records.

Raw data represents all information essential for the reconstruction and evaluation of clinical studies, and it is the original record of clinical findings, observations, or other activities. Examples of these original documents and data records include, but are not limited to: hospital records, clinical and Office software charts, laboratory records, memos, questionnaires or evaluation checklists of subjects, pharmacy dispensing records, recordings of consultation meetings, data records from automated instruments, photocopies or transcriptions verified as accurate and complete, microfilms, photographic negatives, microfilm rolls or disks, X-rays, CT or MRI, ECG records, and files and records of subjects kept in pharmacies, laboratories, and medical technology departments involved in the study.

Such documents must at least indicate the subject number and the date of the operation performed. Where possible, medical evaluations of these records should be documented, and signed and dated by the investigator.

The investigator is responsible for ensuring that the source data is accurate, clear, contemporaneous, original, and attributable, whether the data is handwritten or entered electronically.

14. Quality Assurance and Quality Control

To ensure the quality of the study, a clinical study plan has been developed before the formal start of the study. Training has been provided to the relevant study personnel involved in the study. Training topics include, but are not limited to: GCP, AE/SAE reporting, study protocol, eCRF completion, original document recording, informed consent, and drug management.

Each study site must manage study drugs according to SOP, including the receipt, storage, distribution, recovery, and destruction (if applicable). According to GCP guidelines, necessary measures should be taken during the design and implementation phases of the study to ensure that the data collected is accurate, consistent, complete, and reliable. All observed results and abnormal findings in the clinical study should be verified and recorded in a timely manner to ensure the reliability of the data. Instruments, equipment, reagents, and standards used in various tests and examinations in the clinical study should follow strict quality standards and function normally.

Information required by the protocol should be entered into eCRF by the investigator, which is then verified by the study monitor for completeness and accuracy, and the study site staff are guided to make necessary corrections and supplements.

The drug regulatory authority, Institutional Review Board (IRB)/Independent Ethics Committee (IEC), monitors, and/or auditors may conduct systematic inspections of study-related activities and documents to evaluate whether the study is conducted in accordance with the protocol, SOPs, and relevant regulatory requirements, and whether the study data is recorded in a timely, true, accurate, and complete manner. Audits should be conducted by personnel not directly involved in the clinical study.

15. Ethics and Informed Consent

15.1 Ethical Codes

Without the consent of the sponsor, no site may modify the study protocol on its own.

Only to eliminate direct and immediate harm to the subjects, the investigator may make changes or deviations to the study protocol before obtaining approval from the EC/IRB. At the same time, the deviations or changes made and their reasons, and the proposed amendments to the protocol should be promptly submitted to the EC/IRB for review.

All possible serious violations should be reported as soon as possible. Serious violations refer to violations of GCP conditions and principles related to the study or violations of the study protocol, which may have a significant impact on the safety or mental and physical health of the study subjects or the scientific value of the study.

Personnel involved in the implementation of this study must have the appropriate qualifications; their educational background, training experience, and experience in their respective work must meet the requirements.

15.2 Institutional Review Board/Independent Ethics Committee

Before the initiation of the study, the investigator must first obtain written approval/consent dated by the IRB/IEC for the study protocol, ICF, subject recruitment materials (if any), and all other written materials to be provided to the subjects.

The investigator or Hengrui must submit reports, updates, and other materials (e.g., expedited safety reports, amendments, and administrative letters) to the IRB/IEC in accordance with the requirements of the competent authorities or the institution's procedures.

During the clinical study, any modifications made to the protocol should be submitted to the IRB/IEC, and if necessary, other study documents should be revised accordingly at the same time, and submitted and/or approved according to the requirements of the IRB/IEC.

The investigator is responsible for submitting regular interim reports of the study to the IRB/IEC according to relevant requirements, and should notify the IRB/IEC that the study has ended after its completion.

15.3 Informed Consent

15.3.1 ICF and other written information required for subjects

The ICF will provide a detailed description of the study treatment and procedures, and provide a full explanation of the study risks to the subjects. Before administering the study medication, written documentation of informed consent must be obtained.

All revisions to the ICF must also be approved by the EC, and necessity of re-signing new ICF by subjects will be determined by the EC..

Confidentiality of subjects' personnel information should be described in the informed consent form, but Hengrui's medical representatives or regulatory authorities may access the subjects' information.

15.3.2 Informed consent process and records

Informed consent begins before an individual's consent to participate in a clinical study and continues throughout the entire course of the clinical study. The risks involved in the study and the potential benefits will be discussed in detail and fully with the subjects and their dependents.

Subjects will be asked to read and review the ICF approved by the EC. The investigator will explain the clinical study to the subjects and answer any questions they may have. Subjects will have the opportunity to carefully review the written ICF and ask questions before signing. Subjects will have the opportunity to discuss the study with their representatives and to think it over thoroughly before agreeing to participate. Subjects can only begin participating in the study after signing the ICF. During the entire process of the clinical study, subjects may withdraw consent at any time. A copy of the ICF will be retained by the subjects. Even if the consulted patients refuse to participate in this study, their rights will be fully protected; they will be emphasized that the quality of their medical care will not be affected in any way.

15.3.3 Confidentiality of subject information

The subjects' information will be kept strictly confidential by the investigator and study personnel. Confidentiality also encompasses biological samples and genetic tests, in addition to the subject's clinical information. Therefore, the study protocol, documents, data, and all other information generated will be kept strictly confidential. Without the prior written approval of the investigator, no related study or data information may be disclosed to any unauthorized third parties.

Study monitors, auditors, or IRBs may inspect all documents and records required to be maintained by the investigator, which include but are not limited to: medical records and subjects' medication records. The study site should allow access to these records.

The contact information of subjects will be securely stored at each study site and used internally only during the study process. At the end of the study, all records will continue to be stored securely for the duration specified by the local IRB and regulations.

16. References

[1]. He Jie. Analysis of the Epidemic of Malignant Tumors in China in 2015 [J]. Chinese Journal of Oncology, 2019, 41 (1): 19-28;
[2]. Chinese Society of Clinical Oncology (CSCO) Guidelines for Diagnosis and Treatment of Esophageal Cancer, 2020 edition;
[3]. NCCN Clinical Practice Guidelines in Oncology. Esophageal and Esophagogastric Junction Cancer, 2020 Version 1;
[4]. van Hagen P, et al. Preoperative chemoradiotherapy for esophageal or junctional cance [J]. N Engl J Med, 2012, 366 (22): 2074-2084;
[5]. Shapiro J, et al. Neoadjuvant chemoradiotherapy plussurgery versus surgery alone for oesophageal or junctional cancer (CROSS): longterm results of a randomised controlled trial [J]. Lancet Oncol, 2015, 16 (9): 1090-1098;
[6]. Motz GT, et al. Deciphering and reversing tumor immune suppression [J]. Immunity, 2013, 39(1): 61-73;
[7]. Jing Huang, Jianming Xu, et al; Camrelizumab versus investigator's choice of chemotherapy as second-line therapy for advanced or metastatic oesophageal squamous cell carcinoma (ESCORT): a multicentre, randomised, open-label, phase 3 study [J]. Lancet Oncol, 2020, 21(6):832-842;
[8]. 2019ASCO Abs4058.
[9]. A M Mandard, et al; Pathologic assessment of tumor regression after preoperative chemoradiotherapy of esophageal carcinoma. Clinicopathologic correlations [J]. Cancer, 1994, 73(11): 2680-2686.
[10]. E A Eisenhauer, et al; New response evaluation criteria in solid tumours: revised

RECIST guideline (version 1.1) [J]. Eur J Cancer, 2009, 45(2):228-247.
[11]. Maurer W, Bretz F. Multiple testing in group sequential trials using graphical approaches. Stat Biopharm Res 2013;5(4):311-20.

STATISTICAL ANALYSIS PLAN
(SAP)

A RANDOMIZED, OPEN-LABEL, PARALLEL-CONTROLLED CLINICAL STUDY OF CAMRELIZUMAB COMBINED WITH NEOADJUVANT CHEMOTHERAPY VERSUS NEOADJUVANT CHEMOTHERAPY ALONE FOR RESECTABLE LOCALLY ADVANCED THORACIC ESOPHAGEAL SQUAMOUS CELL CARCINOMA

ESCORT-NEO/EC-004

VERSION 1.0, 16 JAN 2023

CONFIDENTIALITY STATEMENT

The information is provided to you in confidence to enable you to perform the work. Do not give this document or any copy of it or reveal any proprietary information contained in it to any third party or person without the prior permission of an authorization.

APPROVAL FORM

**Prepared By:**		
**Print Name/Role**	**Signature**	**Date**
**Approved By:**		
**Print Name/Role**	**Signature**	**Date**
**Print Name**	**Signature**	**Date**

DOCUMENT REVISION HISTORY		
**Document Version**	**Document Date**	**Reason for Revision**
1.0	16JAN2023	First version – Not applicable

TABLE OF CONTENTS

APPROVAL FORM 65
LIST OF ABBREVIATIONS AND DEFINITIONS OF TERMS 69
1. INTRODUCTION 71
2. STUDY OBJECTIVES 72
2.1. PRIMARY OBJECTIVES 72
2.2. SECONDARY OBJECTIVES 72
2.3. EXPLORATORY OBJECTIVES 72
3. STUDY DESIGN AND METHODS 73
3.1. GENERAL STUDY DESIGN AND PLAN 73
3.2. RANDOMIZATION 74
3.3. BLINDING 74
3.4. SCHEDULE OF EVENTS 75
4. STUDY ENDPOINTS 76
4.1. EFFICACY ENDPOINT(S) 76
4.1.1. Primary Efficacy Endpoint(s) 76
4.1.1.1. Primary Efficacy Endpoints 76
4.1.1.2. Estimands 76
4.1.2. Secondary Efficacy Endpoint(s) 77
4.2. SAFETY ENDPOINT(S) 77
4.3. HEALTH ECONOMICS AND OUTCOME RESEARCH ENDPOINT(S) 78
5. SAMPLE SIZE DETERMINATION 79
6. PLANNED ANALYSIS 81
6.1. INTERIM ANALYSES AND DATA MONITORING 81
6.2. FINAL ANALYSIS 82
7. GENERAL AND STATISTICAL CONSIDERATIONS 83
7.1. ANALYSIS SETS 83
7.1.1. Intention-to-treat Set 83
7.1.2. Per-Protocol Analysis Set 83
7.1.3. Safety Analysis Set 83
7.1.4. Surgical Analysis Set 83
7.2. GENERAL CONSIDERATIONS 83
7.2.1. Reference Start Date, End Date and Study Day 83
7.2.2. Baseline 83
7.2.3. Definition and Use of Visit Windows 84
7.2.4. Repeated or Unscheduled Assessments of Safety Parameters 84
7.3. STATISTICAL CONSIDERATIONS 84
7.3.1. Missing Date or Incomplete Date 84
7.3.1.1. Missing or Incomplete Date Information of Study Treatment84
7.3.1.2. Incomplete Date Information for Prior and Concomitant Medications 84

7.3.1.3. Incomplete Date Information for Adverse Events 85
7.3.1.4. Incomplete death date 85
7.3.1.5. Incomplete PD date 86
7.3.2. Character Values of Clinical Laboratory Tests 86
7.3.3. Computing Methods and Reporting Conventions 86
7.3.3.1. Statistical Summary Conventions 86
7.3.3.2. General Reporting Conventions 86
7.3.4. Subgroups 86
7.3.5. Multiple Comparisons/Multiplicity 87
8. STATISTICAL ANALYSIS 88
8.1. SUMMARY OF STUDY DATA 88
8.1.1. Subject Disposition 88
8.1.2. Protocol Deviations 88
8.1.3. Demographic and Baseline Characteristics 88
8.1.4. Treatment Compliance 89
8.2. EFFICACY ANALYSES 89
8.2.1. Analysis of Primary Estimands 89
8.2.1.1. pCR relevant estimand 89
8.2.1.2. EFS relevant estimand 90
8.2.2. Analysis of Secondary Efficacy Endpoints 93
8.2.2.1. MPR 93
8.2.2.2. R0 resection rate and pathologic staging after neoadjuvant therapy based on AJCC 8th edition (ypTNM staging) 93
8.2.2.3. OS and DFS 93
8.3. SAFETY ANALYSES 93
8.3.1. Dosing and Extent of Exposure 94
8.3.2. Adverse Events 95
8.3.2.1. Treatment-related TEAEs 96
8.3.2.2. Serious adverse events 97
8.3.2.3. TEAE leading to study drug interruption, dose reduction and discontinuation 97
8.3.2.4. Immune related TEAEs 97
8.3.2.5. Death, Other Serious Adverse Events, and Other Significant Adverse Events 97
8.3.3. Clinical Laboratory Evaluations 97
8.3.4. Vital Sign 98
8.3.5. ECG and Echocardiogram 98
8.3.6. Eastern Cooperative Oncology Group (ECOG) PS 99
8.3.7. Surgical safety relevant endpoints 99
8.4. PATIENTS REPORTED OUTCOME (PRO) ANALYSIS 99
8.4.1. EORTC QLQ-C30 99
8.4.2. EORTC QLQ-OES18 100
9. SUMMARY OF CHANGES TO THE STATISTICAL ANALYSES SPECIFIED IN PROTOCOL 101
10. REFERENCES 102
11. APPENDICES 103

LIST OF ABBREVIATIONS AND DEFINITIONS OF TERMS

ABBREVIATION	DEFINITION
AE	Adverse event
AJCC	American Joint Committee on Cancer
ATC	Anatomical Therapeutic Chemical
BIRC	Blinded Independent Review Committee
BMI	Body mass index
BSA	Body surface area
CM	Concomitant medications
CR	Complete response
DFS	Disease-free survival
ECOG-PS	Eastern Cooperative Oncology Group- Performance Status
eCRF	Electronic case report form
EFS	Event-free survival
EORTC QLQ-C30	European Organisation for the Research and Treatment of Cancer Quality of Life Questionnaire Core 30
EORTC QLQ-OES18	European Organization for Research and Treatment of Cancer Quality of Life Questionnaire-Oesophageal Module 18
EX	Exposure
HR	Hazard Ratio
ICH	The International Council for Harmonisation of Technical Requirements for Pharmaceuticals for Human Use
iDMC	Independent data monitoring committee
ITT	Intent to treat analysis
LVEF	Left ventricular ejection fraction
MedDRA	Medical Dictionary for Regulatory Activities
MPR	Major pathological response
NCI-CTCAE v5.0	National Cancer Institute - Common Terminology Criteria for Adverse Events Version 5.0
OS	Overall survival
pCR	Pathological complete response
PD	Progressive disease
PPS	Per-Protocol Set
PRO	Patient reported outcome
PT	Preferred term
RECCP	Reactive cutaneous capillary endothelial proliferation
RECIST v1.1	Response Evaluation Criteria in Solid Tumors
RS	Raw score
SAE	Serious adverse event
SURG	Surgery analysis set
SOC	System organ class
SS	Safety set

TEAE	Treatment Emergent Adverse Event
TESAE	Treatment Emergent Serious Adverse Event
TMB	Tumor mutational burden
TRAE	Treatment related adverse event
WHO	World Health Organization

1. INTRODUCTION

This statistical analysis plan (SAP) is developed mainly for the purpose of the primary analysis of pathologic complete response (pCR), the interim and final analysis of event-free survival (EFS). The SAP provides a detailed, technical elaboration of the statistical analyses of efficacy and safety data as described in the study protocol Version 3.0 dated 15APR2022. Specifications for tables, listings, and figures are contained in a separate document.

The primary analysis of pCR will be initiated when the 390 subjects being randomized have received surgery, refused surgery or cannot receive surgery due to any reason.

The interim analysis of event-free survival (EFS) will be initiated when 70% of preplanned [redacted] EFS events being observed.

The final analysis will be initiated when 228 EFS events being observed.

2. STUDY OBJECTIVES

2.1. Primary Objectives

- To compare the perioperative regimen of camrelizumab combined with neoadjuvant chemotherapy and postoperative camrelizumab adjuvant therapy versus neoadjuvant chemotherapy with paclitaxel and cisplatin for patients with resectable esophageal squamous cell carcinoma in terms of pathologcal complete response (pCR) and Event-Free Survival (EFS).

2.2. Secondary Objectives

- To compare the perioperative regimen of camrelizumab combined with neoadjuvant chemotherapy and postoperative camrelizumab adjuvant therapy versus neoadjuvant chemotherapy with paclitaxel and cisplatin for patients with resectable esophageal squamous cell carcinoma in terms of major pathological response (MPR), R0 resection rate, pathological stage after neoadjuvant therapy (ypTNM stage), disease-free survival (DFS), overall survival (OS), and safety and tolerability.

2.3. Exploratory Objectives

- To explore the quality of life of patients with resectable esophageal squamous cell carcinoma treated with the perioperative regimen of camrelizumab combined with neoadjuvant chemotherapy and postoperative camrelizumab adjuvant therapy compared to the neoadjuvant chemotherapy with paclitaxel and cisplatin;
- To evaluate the relationship between biomarkers in tumor tissues and response.

3. STUDY DESIGN AND METHODS

3.1. General Study Design and Plan

This is a multicenter, randomized, open-label, parallel-controlled clinical trial to evaluate the efficacy and safety of chemotherapy plus camrelizumab versus chemotherapy alone as neoadjuvant treatment for resectable esophageal squamous cell carcinoma.

Eligible subjects will be randomized at a ratio of 1:1:1 to test Group A (Camrelizumab + albumin-bound paclitaxel + cisplatin), test Group B (Camrelizumab + paclitaxel + cisplatin), and control Group C (Paclitaxel + cisplatin). Detailed information of randomization will be provided in section 3.2.

Subjects being randomized to Group A, Group B, and Group C will receive the following treatments:

Neoadjuvant therapy: 2 cycles of drug administered every 3 weeks as 1 cycle, followed by surgical resection.

- Group A: Camrelizumab (200 mg/dose) + albumin-bound paclitaxel (125 mg/m^2) + cisplatin (75 mg/m^2) on D1; albumin-bound paclitaxel (125 mg/m^2) on D8
- Group B: Camrelizumab (200 mg/dose) + paclitaxel (175 mg/m^2) + cisplatin (75 mg/m^2) on D1
- Group C: paclitaxel (175 mg/m^2) + cisplatin (75 mg/m^2) on D1

Surgical treatment: Subjects will be reassessed by the investigator prior to surgery. Preoperative visits and related assessments should be performed within 14 days prior to surgery. Surgery should be performed within 4-6 weeks after the last administration of neoadjuvant therapy. If surgical treatment is more than 6 weeks beyond the last dose of neoadjuvant therapy, then the preoperative visit and related assessments should be repeated within 14 days prior to surgery. After surgery, surgery for esophageal cancer and tumor regression grade will be assessed.

Postoperative adjuvant therapy:

- Group A and B: Camrelizumab (200 mg/dose) D1 Q3W; the first postoperative dose of camrelizumab should be administered within 4-6 weeks after surgery; camrelizumab was administered up to a maximum of 17 doses in total throughout the study period.
- Group C: Subjects will receive regular follow-up observations.

Imaging Evaluation: The window period allowed for imaging is ± 7 days when not otherwise specified.

- For the screening period, baseline tumor assessment should be completed within 3 weeks prior to randomization;

- After randomization, the conditions for radiological examination should be the same as the baseline (including scan slice thickness, contrast agents, etc.). Tumor radiological assessments should be performed within 14 days before surgery (i.e., within 3-4 weeks after the last dose of neoadjuvant therapy, or within 14 days before surgery if surgery exceeds 6 weeks after the last

dose of neoadjuvant therapy), at 4 weeks after surgery, and before receiving postoperative carelizumab treatment only for test groups A and B, then every 12 weeks (±14 days), and every 24 weeks (±28 days) starting from the third year, until radiological progression/recurrence, withdrawal of informed consent, loss to follow-up, or death, whichever occurs first.

- When radiographic progression occurs for the first time during treatment with carelizumab, after assessment by the investigator and informed consent by the subject, a second imaging examination is recommended at an interval of 4-6 weeks for confirmation if the subject's clinical status is stable.

- When a subject terminates study treatment for reasons other than radiographic progression, imaging should be performed at the end of treatment (unless imaging has already been performed within 28 days) and thereafter at the above frequency whenever possible.

Surgical Evaluation:

Extent of surgical eradication of esophageal cancer, surgical complications, perioperative mortality (within 30 days postoperatively and within 90 days postoperatively), length of hospitalization, and reoperation rate;

Pathologic assessment:

Tumor regression grading assessment (TRG) will be based on Mandard criteria, yp staging by AJCC 8th edition.

Safety follow-up:

- Group A and B: every 30 days until 90 days starting after the last dose of carilizumab.
- Group C: 30 days after surgery for patients who underwent surgery or 30 days after the last dose of study drug for patients who did not undergo surgery.

Only all SAEs considered related to study drug were collected after the safety follow-up period.

Tumor Progression/Recurrence Follow-up: For subjects who terminate treatment for reasons other than radiographic progression/recurrence, they should continue to be followed for time to radiographic progression/recurrence with imaging at the frequency specified in the protocol until radiographic progression/recurrence, initiation of other anti-tumor treatment, withdrawal of informed consent, loss to follow-up, or death.

Survival Follow-Up: After the safety follow-up is completed, survival follow-up will be performed every 3 months until the subject dies, loss to follow up, or the study is terminated.

3.2. Randomization

In the study design, the eligible subjects will be randomly assigned in a 1:1:1 ratio to the Group A, Group B and Group C via block permutation through IWRS. The randomization was stratified by clinical stage (stage I/II vs. stage III vs. stage IVa).

3.3. Blinding

The pathologist and the main statistical team are masked to the randomization information.

3.4. Schedule of Events

The schedule of events could be referred to the “Study flow chart” in the protocol.

4. STUDY ENDPOINTS

4.1. Efficacy Endpoint(s)

4.1.1. Primary Efficacy Endpoint(s)

4.1.1.1. Primary Efficacy Endpoints

- pCR assessed by the Blinded Independent Review Committee (BIRC), defined as the percentage of subjects with no tumor cells (Mandard Criteria Grade 1) in the primary tumor and histologically negative lymph nodes.
- EFS assessed by the investigator according to RECIST 1.1, defined as the time from the date of randomization to the occurrence of disease progression, recurrence, or death (whichever occurs first).

  Note: A second primary malignancy, or a radiographic progression occurring during the neoadjuvant phase that does not interfere with radical resection of esophageal cancer, is not considered as an EFS event.

4.1.1.2. Estimands

Variable	pCR assessed by BIRC			EFS based on RECIST v1.1 assessed by investigators		
Treatment	See section 3.1					
Population	Subjects without prior systematic treatment, pathohistologically or cytologically confirmed resectable locally advanced thoracic esophageal squamous cell carcinoma (clinical stage T1b-3N1-3M0 or T3N0M0 according to AJCC 8th edition)					
Population level summary	Difference or Odds ratio of pCR rate between groups			Hazard ratio of EFS between groups		
	Intercurrent Events	Strategies	Description	Intercurrent events	Strategies	Description
Intercurrent events and strategies	Drop-out before surgery due to disease progression and any other reason	Hypothetical strategies	If the subject receives surgery, the pathological result would indicate non-pCR.	End of treatment	Treatment policy strategies	Data collected after treatment termination will be used for the analysis

Intercurrent events and strategies	Use other anti-tumor therapy before surgery	Hypothetical strategies	If the subject doesn't use other anti-tumor therapy before surgery, the pathological result would indicate non-pCR	Use other anti-tumor therapy	While on treatment strategies	Data collected before the initiation of other anti-tumor therapy will be used for the analysis
Intercurrent events and strategies	/	/	/	Death from non-disease causes	Composite strategies	If there are no special circumstances, it will be considered as an EFS event

4.1.2. Secondary Efficacy Endpoint(s)

- MPR assessed by assessed by BIRC, defined as the percentage of subjects with residual tumor <10% in the primary tumor;
- R0 resection rate, defined as the percentage of subjects with no residual tumor;
- Pathologic staging after neoadjuvant therapy based on AJCC 8th edition (ypTNM staging);
- OS, defined as the time from randomization to death from any cause
- DFS, defined as the surgery time to the time of local or distance recurrence, or death from any cause, whichever occurs first.

Patients with postoperative recurrence should be confirmed through cytological or histological examination whenever possible.

The second primary tumor and non-R0 resection are not considered as events for EFS and DFS.

4.2. Safety Endpoint(s)

- AEs: All SAEs/AEs that occur during the study, including symptoms and signs at screening, whether related to the study drug or not, must be recorded in the CRF.
- Surgical complications, perioperative mortality: Surgical complications are recorded within 30 days after surgery or during the hospital stay after surgery, and deaths are recorded within 90 days after surgery.

4.3. Health Economics and Outcome Research Endpoint(s)

- European Organization for Research and Treatment of Cancer Quality of Life Questionnaire-C30 (EORTC QLQ-C30)
- Supplementary Scale for Patients with Esophageal Cancer (EORTC QLQ-OES18)
- To explore the relationship between potential biomarkers in tumor tissue (including but note limited to PD-L1 expression, MMR status, TMB, EBER) and efficacy.

5. SAMPLE SIZE DETERMINATION

Statistical Hypotheses:

The study is a superiority design, and statistical tests for the dual primary study endpoints of pCR and EFS are performed using a multiplicity test. The multiplicity strategy is described in Section 7.3.5. This study includes two primary hypotheses and three secondary hypotheses.

The two primary hypotheses:

1) pCR: Group A vs. Group C

H_{01}: The pCR rate of Group A is lower than or equal to the pCR rate of Group C.

H_{11}: The pCR rate of Group A is higher than the pCR rate of Group C.

2) EFS: Group A+B vs. Group C

H_{02}: The HR of Group A+B to group C is greater than or equal to 1.

H_{12}: The HR of Group A+B to Group C is less than 1.

The three secondary hypotheses:

3) pCR: Group B vs. Group C

H_{03}: The pCR rate of Group B is lower than or equal to the pCR rate of Group C.

H_{13}: The pCR rate of Group B is higher than the pCR rate of Group C.

4) EFS: Group A vs. Group C

H_{04}: The HR of Group A to group C is greater than or equal to 1.

H_{14}: The HR of Group A to Group C is less than 1.

5) EFS: Group B vs. Group C

H_{05}: The HR of Group B to group C is greater than or equal to 1.

H_{15}: The HR of Group B to Group C is less than 1.

Sample size:

For pCR: Assuming a pCR rate of 30% for Group A, 25% for Group B, and 9% for Group C. Randomization ratio is 1:1:1. At a significance level of α=0.5% (one-sided), 111 subjects in each group are expected to provide at least 93% power to detect that test group A is superior to control group C, and can provide at least 75% power to detect that test group B is superior to control group C. Considering a 15% dropout rate, 130 subjects per group would be required.

For EFS: the median EFS time in Group C is assumed to be 30 months. If this study assumes a hazard ratio (HR) of 0.67 for Group A+B compared to Group C, with a significance level (α) of 0.02 (one-sided), an interim analysis of the effectiveness of the EFS is planned to be conducted when 70% of the number of events are observed;

- If the randomization ratio is 2:1 (Group A+B vs. Group C), 228 events (141 events in the trial group and 87 events in the control group) would be required to provide at least 80% power to

detect the superiority of Group A+B over Group C. The planned enrollment period is 36 months, and the total study duration is 84 months. Considering a dropout rate of 15%, a total of approximately 390 subjects would be required.

- With a 1:1 randomization ratio (Test Group A vs. Group C or Test Group B vs. Group C), 158 events (71 in the Test Group and 87 in the Group C) could be observed in the above sample size to provide at least 67% power to detect the superiority of either Test Group A or Test Group B to Group C.

6. PLANNED ANALYSIS

6.1. Interim Analyses and Data Monitoring

The interim analysis of the effectiveness of EFS will be performed when 70% of the EFS events [redacted] are to be observed (Group A+B vs. Group C). There are two scenarios:

(1) If both hypotheses H_{01} and H_{03} regarding pCR are successfully rejected, EFS will be tested at the one-sided $\alpha = 0.025$ level for Group A+B vs. Group C.

In order to control the overall Type I error (α) not exceeding the predetermined one-sided α = 0.025, the O'Brien & Fleming method approximated by Lan-DeMets α spending function will be used to calculate the efficacy interim analysis boundaries as shown in the table below. [redacted]

[redacted]

(2) If either hypotheses H_{01} or H_{03} regarding pCR cannot be successfully rejected, EFS will be tested at the one-sided $\alpha = 0.02$ level for Group A+B vs. Group C.

[redacted]

[redacted]

[redacted]

[redacted]	[redacted]	[redacted]	[redacted]	[redacted]
[redacted]	[redacted]	[redacted]	[redacted]	[redacted]
	[redacted]	[redacted]	[redacted]	[redacted]
[redacted]	[redacted]	[redacted]	[redacted]	[redacted]
	[redacted]	[redacted]	[redacted]	[redacted]

If no significant difference in EFS between Group A+B and Group C is detected in the interim analysis, the final analysis of EFS will be conducted when 228 EFS events are observed.

If the analysis indicates that Group A+B has superior EFS to Group C (rejection of H_{02}), the corresponding α can be passed on to the test of Group A vs. Group C (H_{04}), and so on to the test of Group B vs. Group C (H_{05}).

The final analysis of the pCR and the interim analysis of the EFS will be conducted by an independent statistical team and the results will be evaluated by the Independent Data Monitoring Committee (iDMC), please refer to the Statistical Analysis Plan and the iDMC Charter for a more detailed plan for the final analysis of the pCR and the interim analysis of the EFS.

6.2. Final Analysis

The primary analysis of pCR will be initiated when the 390 subjects being randomized have either completed the surgery, or refused surgery/cannot receive surgery due to any reason.

The final analysis of EFS will be performed after all subjects have been enrolled and followed for 48 months, at which time it is expected that at least 228 EFS events will have been collected in Group A+B and Group C.

7. GENERAL AND STATISTICAL CONSIDERATIONS

7.1. Analysis Sets

7.1.1. Intention-to-treat Set

Intention-to-treat Set (ITT): ITT will include all subjects randomized. ITT will be the main analysis set for the efficacy analysis, and the subjects in the ITT set will be analyzed according to the ITT principal.

7.1.2. Per-Protocol Analysis Set

Per-protocol Analysis Set (PPS): PPS is a subset of the ITT. PPS will include all subjects in the ITT who have no major protocol deviation which may have a significant impact on the study results. The criteria which may result in the subjects being excluded from PPS will be finalized before Database-Lock (DBL), and the list of subjects being included or excluded from PPS will be reviewed and finalized by Hengrui and the investigators before Database-Lock.

7.1.3. Safety Analysis Set

Safety Analysis Set (SS): SS will include all randomized subjects who received study treatment.

7.1.4. Surgical Analysis Set

Surgical Analysis Set (SURG): SURG is a subset of ITT. SURG will include all subjects who received radical surgery prespecified in the protocol and with assessable pathological specimen. SURG will serve as the main analysis set of surgery relevant endpoints.

7.2. General Considerations

7.2.1. Reference Start Date, End Date and Study Day

Reference start date and end date:

- Reference start date is the first date of study treatment administration
- Reference end date is the last date of study treatment administration.

Study day will be calculated based on the reference start date:

- Study day = event/assessment date – first study drug administration date + (event/assessment date ≥ first study drug administration date)

7.2.2. Baseline

In general, baseline is defined as the last assessment before first date of study treatment administration, unless otherwise specified. If no study treatment is administrated for a randomized subject, baseline is defined as the last assessment before randomization.

7.2.3. Definition and Use of Visit Windows

All the by-visit analysis will use the original visits being recorded in the CRF. No derivation of visit will be performed.

7.2.4. Repeated or Unscheduled Assessments of Safety Parameters

In general, all the by-visit summaries will only present the data being recorded in scheduled visits. Measurements recorded in unscheduled visits will not be included in the by-visit summaries, but will contribute to determine the best/worst case value if required (i.e. shift table).

Listings will include all the scheduled and unscheduled data.

7.3. Statistical Considerations

7.3.1. Missing Date or Incomplete Date

In general, the incomplete date will be imputed following the below rules unless otherwise specified if it has impact on the statistical analysis:

- Impute 15 to the day part if only day is missing;
- Impute JUL 01 to the month and day if both month and day is missing;
- Will not impute if completely missing.

7.3.1.1. Missing or Incomplete Date Information of Study Treatment

In general, the date of study treatment should not be imputed. If date of study treatment is missing or partial missing, all the efforts should be made to obtain the date.

If the last date of study treatment administration is missing due to data cut-off, the date should be imputed to the cut-off date.

7.3.1.2. Incomplete Date Information for Prior and Concomitant Medications

The date of concomitant medication will only be imputed when partial missing.

7.3.1.2.1. Incomplete Start Dates

- Missing Day and/or Month: impute to the earliest possible date.
- Complete missing date will not be imputed and will be considered as concomitant medication.

In case the imputed start date is after the stop date, then the start date will be imputed using the stop date.

7.3.1.2.2. Incomplete Stop Dates

- Month and day are missing: impute to Dec 31.
- Only day is missing: impute to the last day of the month
- Only month is missing: impute to Dec.

The imputed date should not be later than the last date known to be alive, death date and end of study date.

7.3.1.3. Incomplete Date Information for Adverse Events

7.3.1.3.1. Incomplete Start Dates

- Missing Day and Month
  - If the year of the incomplete date is the same as the year of the first date of study treatment administration, then impute to the date of first study treatment administration.
  - If the year of the incomplete date is prior to the year of the first date of study treatment administration, then impute to the Dec 31.
  - If the year of the incomplete date is after the year of the first date of study treatment administration, then impute to Jan 1.
- Missing Day Only
  - If the known parts of the incomplete date is the same as the corresponding parts of the first date of study treatment administration, then impute to the date of first study treatment administration.
  - If the known parts of the incomplete date is prior to the corresponding parts of the first date of study treatment administration, then impute to the last day of the month.
  - If the known part of the incomplete date is after the year of the first date of study treatment administration, then impute to the first day of the month.

In case the imputed start date is after the stop date, then the start date will be imputed using the stop date.

7.3.1.3.2. Incomplete Stop Dates

- If completely missing or the year is missing: will not impute;
- If month and/or day is missing: impute to the latest possible date.

The imputed date should not be later than the last date known to be alive, death date and end of study date.

7.3.1.4. Incomplete death date

- If only the day is missing: impute to the first day of the month
- If both the month and day are missing: impute with Jan 01
- If completely missing: impute with the next day of last date known to be alive.

The imputed date should be later than the last date known to be alive.

7.3.1.5. Incomplete PD date

The incomplete PD date will only be imputed in case that year is not missing. The incomplete PD date will be imputed to the earliest possible date. In case of the imputed date is later than death date, death date will be used to impute the PD date.

If PD date is completely missing and no previous PD occurred, the subjects will be considered to be censored.

7.3.2. Character Values of Clinical Laboratory Tests

If the reported value for a clinical laboratory test is in form of a character string, then the numeric part of the string will be used for the analysis purpose.

If the result is reported as below-limit-of quantification (BLQ), then the lower boundary of the quantifiable assessment range will be used for the analysis purpose. However, the actual values as reported in the database will be presented in data listings.

7.3.3. Computing Methods and Reporting Conventions

All the statistical analysis will be performed with SAS® Version 9.4 or above.

7.3.3.1. Statistical Summary Conventions

For continuous variables, descriptive statistics including number of subjects with non-missing values, mean, standard deviation, median, minimum, and maximum, will be presented. For categorical variables, frequencies and percentages will be presented. Time-to-events data will be analyzed by Kaplan-Meier method, and K-M plots will be presented if necessary.

7.3.3.2. General Reporting Conventions

The means and medians should have 1 more decimal place than the observed values, the standard deviations should have 2 additional decimal place than the observed values. Min and Max should have the same decimal place with the observed values.

p-values will be reported to 4 decimal places, the p-values less than 0.0001 will be reported as "<0.0001", while the p-values greater than "0.9999" will be reported as ">0.9999".

7.3.4. Subgroups

Subgroup analysis will be conducted for PFS and OS, which will be described in corresponding sections. It should be noted that the study is not designed to detect the difference in treatment within subgroups. If the subject number of the group is less than 10 or with other considerations, it maybe pooled with others.

The following subgroups will be assessed for OS and PFS:

- Sex (Male vs. Female)
- Age (< 65 years vs. ≥ 65 years)
- Weight (< 60 kg vs. ≥ 60 kg)
- BMI (<24 kg/m^2 vs. ≥ 24 kg/m^2)

- ECOG (0 vs. 1)
- Clinical stage (I-II vs. III vs. IVa)
- T stage (T1b vs. T2 vs. T3)
- N stage (N0 vs. N1 vs. N2 vs. N3)
- Tumor location (Upper vs. Middle vs. Lower)
- Histologic grade (G1 vs. G2 vs. G3 vs. Gx)
- PD-L1 TPS (< 1% vs. ≥ 1%, < 5% vs. ≥ 5%, < 10% vs. ≥ 10%)
- PD-L1 CPS (< 1 vs. ≥ 1, < 5 vs. ≥ 5, < 10 vs. ≥ 10)

7.3.5. Multiple Comparisons/Multiplicity

The sequential testing will be applied to the two primary hypotheses H_{01}, H_{02} and three secondary hypotheses H_{03}, H_{04}, H_{05} of this study, see Section 5 for details of the statistical hypotheses.

The overall Type I error (α) for the two primary hypotheses is controlled at 2.5% (one-sided), 0.5% is assigned to the pCR hypothesis for Group A vs. Group C, and 2% is assigned to the EFS hypothesis for Group A+B vs. Group C.

Assigning and passing α across multiple hypotheses using the graphical method of Maurer and Bretz. Under this method, the study hypotheses may be tested more than once, and when an original hypothesis is rejected, the α assigned to that hypothesis can be passed on to another hypothesis test.

The figure below (figure 1) represents each hypothesis and its initial one-sided α allocation, and the circles above the lines connecting the hypotheses represent the weights that each hypothesis reassigns to the other hypotheses.

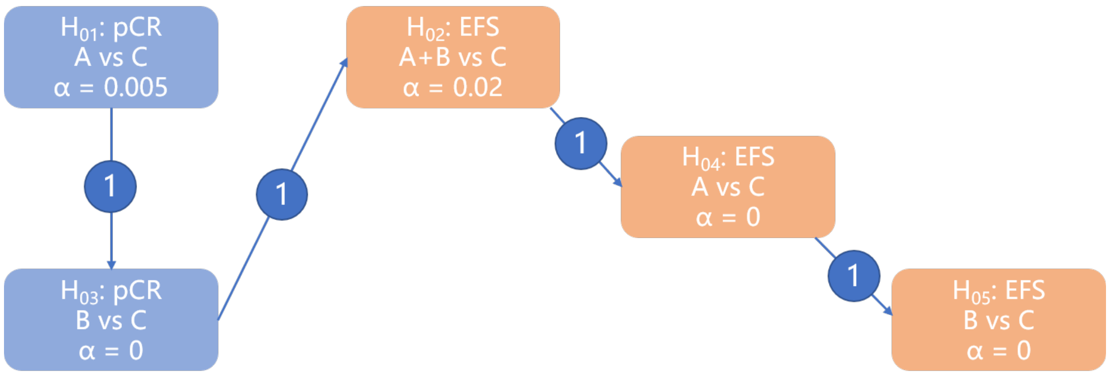

Figure 1 Overview of the Type I error control for pCR and EFS.

The type-I error rate is controlled by using Lan-DeMets method to spending alpha at the interim analysis for EFS. Interim analysis details can be found in Section 6.1.

8. STATISTICAL ANALYSIS

8.1. Summary of Study Data

8.1.1. Subject Disposition

The number of subjects being screened will be summarized, the number and percentages of subjects who failed screening as well as reasons of screen failure will also be summarized. The number and percent of subjects being randomized, received treatment, completed/not completed neoadjuvant treatment, received surgery, received adjuvant treatment, still on study treatment discontinued treatment will be summarized by treatment group. The reason for not completing neoadjuvant treatment, not receiving surgery, early termination of study treatment will be further summarized. The number and percentage of subject in each analysis set (ITT, PPS, SURG and SS) will be summarized by treatment group. In addition, the duration of subject in study will also be summarized.

- Duration in study (months) = (Last day of known to be alive – randomization date + 1)/30.4375

Data listing of analysis set, as well as end of treatment and end of study will be provided.

8.1.2. Protocol Deviations

Protocol deviations will be summarized by the treatment group and overall, and furthermore summarized by severity. Major protocol deviation leading to the exclusion of subjects from PPS will also be summarized by treatment group. Protocol deviations will be listed, and the major protocol deviations will be flagged.

8.1.3. Demographic and Baseline Characteristics

Demographic and baseline characteristics listed below will be summarized by treatment group and overall for ITT.

- Age as continues variable
- Age as categorical variable (< 65-year, ≥ 65-year)
- Gender (male, female)
- Weight (< 55 kg, ≥ 55 kg)
- Baseline ECOG PS (0, 1)
- Baseline Body Mass Index (BMI) (<24 mg/kg^2 vs. ≥24 mg/kg^2)
- Baseline Body Surface Area (m^2)

The esophageal squamous cell carcinoma history information listed below will be summarized by treatment group and overall for ITT.

- Time since primary diagnosis (month) to the randomization date
- Histological classification (squamous cell carcinoma vs. adenocarcinoma vs. other)

- Tumor location (cervical vs. upper thoracic vs. middle thoracic vs. lower thoracic vs. esophagogastric junction area)
- Histological grade (G1 vs. G2 vs. G3 vs. Gx)
- T stage (TX vs. T0 vs. Tis vs. T1a vs. T1b vs. T2 vs. T3 vs. T4a vs. T4b)
- N stage (NX vs. N0 vs. N1 vs. N2 vs. N3)
- Site of metastasis
- Clinical stage (0 vs. I vs. II vs. III vs. IVA vs. IVB)

The derivation of BMI and time since primary diagnosis are listed as below:

- BMI (kg/m^2) = weight (kg)/height $(m^2)^2$
- BSA (m^2) (male) = 0.00607*height (cm) + 0.0127*weight (kg) – 0.0698
- BSA (m^2) (female) = 0.00586*height (cm) + 0.0126*weight (kg) – 0.0461
- Time since primary diagnosis (month) = (Date of randomization – first time of disease diagnosis + 1)/(365.25/12)

Data listings for demographic information, disease diagnosis information as well as prior anti-cancer therapy will be provided.

8.1.4. Treatment Compliance

See section 8.3.1.

8.2. Efficacy Analyses

8.2.1. Analysis of Primary Estimands

The definition of estimands can be found in section 4.1.1.

8.2.1.1. pCR relevant estimand

8.2.1.1.1. Main estimator

ITT will be used as the primary analysis set for the primary analysis of pCR.

The pCR rate and its 95% Clopper-Pearson confidence interval (CI) will be calculated for each treatment group, and the pCR rate of Group A and Group C, Group B and Group C will be compared by using the stratified CMH test, and the stratification factors will be clinical stage (stage I/II vs. stage III vs. stage IVa). Although the p value will be presented in the 2-sided manner, 1-sided p value will be used for the decision-making purpose.

The differences in pCR rates between Group A/ Group B and Group C, and the corresponding 95% CIs for rate differences (using the stratified mantel-haenszel method) will be presented. The common odds ratio of pCR rate of Group A/ Group B in comparison with Group C, adjusted by the stratification factors, and its confidence interval will also be calculated by Mantel-Haenszel method.

8.2.1.1.2. Sensitivity estimator of pCR

- Unstratified CMH test will be performed to compare the pCR rate between Group A/ Group B and Group C.
- Logistic regression model will be used to adjust for stratification factors and other relevant covariates (if applicable) for sensitivity analysis.

The p-values reported with the sensitivity estimator are nominal p-values

8.2.1.1.3. Supplementary analysis of pCR

- Similar analysis methods described in section 8.2.1.1.1 will be used to analyze pCR in PPS and SURG. The p-values reported in the supplementary analysis are nominal p-values

8.2.1.1.4. Subgroup analysis of pCR

The subgroup listed in section 7.3.4 will be involved in the subgroup analysis in ITT. The number of subjects who achieved pCR will be summarized for each subgroup level in each treatment group. The pCR rate and corresponding Clopper-pearson 95% CI will be presented for each subgroup level in each treatment group, the corresponding odds ratio of or difference in pCR between Group A/ Group B and Group C at each subgroup level will also be presented. The corresponding forest plot will be provided.

8.2.1.2. EFS relevant estimand

[redacted]

[redacted]

[redacted]

[redacted]

[redacted]	[redacted]	[redacted]	[redacted]
[redacted]	[redacted]	[redacted]	[redacted]
[redacted]	[redacted]	[redacted]	[redacted]

[redacted]	[redacted]	[redacted]	[redacted]
[redacted]	[redacted]	[redacted]	[redacted]
[redacted]	[redacted]	[redacted]	[redacted]
[redacted]	[redacted]	[redacted]	[redacted]
[redacted]	[redacted]	[redacted]	[redacted]
[redacted]	[redacted]	[redacted]	[redacted]

	[redacted]		

[redacted]

[redacted]

[redacted]

[redacted]

[redacted]

[redacted]

[redacted]

[redacted]

[redacted]

[redacted]

[redacted]

[redacted]

8.2.2. Analysis of Secondary Efficacy Endpoints

8.2.2.1. MPR

MPR assessed by BIRC and investigator will be analyzed by using the similar methods described in section 8.2.1.1.1. However, the p values reported is the nominal ones.

8.2.2.2. R0 resection rate and pathologic staging after neoadjuvant therapy based on AJCC 8th edition (ypTNM staging)

The number and percentage of subjects with different degree of resection (R0 vs. R1 vs. R2) in each treatment group in SURG will be summarized. The R0 rate and its corresponding clopper-pearson 95% CI will be presented for each treatment group. The difference of R0 rate between the test group and Group C, and the corresponding 95% CIs for rate differences (using the Miettinen-Nurminen method) will also be presented.

The ypTNM staging will be summarized by descriptive statistics for each treatment group in SURG.

8.2.2.3. OS and DFS

OS is defined as the duration from randomization to death. Subjects who are still alive or have lost to follow-up at the time of data cutoff will be censored on the last date known to be alive.

Disease free survival is defined as the duration from surgery to recurrence or death which occurs earlier. The censor rule is the same as those of EFS being described in section 8.2.1.2.1, except for “Disease progression before radical surgery” and “No baseline tumor assessment and no surgery” are not applicable for DFS.

DFS and OS will be analyzed using the similar method being described in section 8.2.1.2.1 based on SURG and ITT respectively. The p-values being reported are the nominal p-values.

8.3. Safety Analyses

All the safety analysis will be summarized by treatment group for preoperative phase (neoadjuvant phase), postoperative phase (adjuvant phase) and treatment phase respectively based on SS except for otherwise specified.

Safety endpoints include treatment-emergent adverse events (TEAE), clinical laboratory tests, vital signs, electrocardiogram (ECG) parameters, echocardiogram (ECHO) parameters, Eastern Cooperative Oncology Group performance status (ECOG PS) and surgical complications.

8.3.1. Dosing and Extent of Exposure

The following items reflecting drug exposure will be summarized by treatment group respectively based on SS:

Table 3. Definition and derivation of chemotherapy for exposure relevant parameters.

Parameter	albumin-bound paclitaxel	paclitaxel	cisplatin
Cycle of exposure	(Last dose date – first dose date + 14)/21	(Last dose date – first dose date + 21)/21	
Actual cycle number of exposure	Sum of cycle number with non-zero dosing of corresponding study drugs		
Total dose (mg/m^2)	Sum of (dose being taken per day/ BSA at the time of medication)		
Dose intensity (mg/m^2/cycle)	Total dose (mg/m^2)/ Cycle of exposure		
Relative dose intensity (%)	100*[dose intensity (mg/m^2/cycle)]/[125*2 mg/m^2/cycle]	100*[dose intensity (mg/m^2/cycle)]/[175 mg/m^2/cycle]	100*[dose intensity (mg/m^2/cycle)]/[75 mg/m^2/cycle]
Compliance (%)	Total dose (mg/m^2)/ prescribed total dose by investigators (mg/m^2)*100%		

Camrelizumab is scheduled to be administered in the preoperative phase (neoadjuvant phase), and postoperative phase (adjuvant phase) for test Groups A and B. The parameters related to drug exposure will be derived for each phase and during the treatment phase are as follows.

Table 4. Definition and derivation of camrelizumab for exposure relevant parameters.

Parameter	Preoperative phase	Preoperative phase	Treatment phase
Cycle of exposure	(Preoperative last dose date – first dose date + 21)/21	(Postoperative last dose date – postoperative first dose date + 21)/21	(Sum of the preoperative and postoperative duration of exposure) /21
Actual cycle number of exposure	Sum of cycle number with non-zero dosing of corresponding study drugs		
Total dose (mg)	Sum of dose being taken per day		
Dose intensity (mg/cycle)	Total dose (mg)/ Cycle of exposure		
Relative dose intensity (%)	100*[dose intensity (mg/cycle)]/[200 mg/cycle]		
Compliance (%)	Total dose (mg)/ prescribed total dose by investigators (mg)*100%		

Moreover, the number of subjects who experienced albumin-bound paclitaxel, paclitaxel and cisplatin interruption, and camrelizumab dose reduction and interruption will be summarized. In addition, the number of interruption and dose reduction will also be summarized appropriately.

Listings of drug exposure will be provided.

8.3.2. Adverse Events

Adverse events (AE) will be coded using Version 24.0 or above of the Medical Dictionary for Regulatory Activities (MedDRA). AE will be summarized by treatment group for randomization phase and extension phase respectively.

- An AE will be considered as a TEAE occurs during preoperative phase if it occurs or becomes worse in severity after the initiation of study treatment and within max (30 days after last study treatment administration, 90 days after last dose of camrelizumab) before surgery or to the date of surgery, which occurs earlier.
- An AE will be considered as a TEAE occurs during the postoperative phase if it occurs or becomes worse in severity after the initiation of postoperative (adjuvant) study treatment and within max (30 days after last study treatment administration, 90 days after last dose of camrelizumab).
- An AE will be considered as a TEAE occurs during study treatment if it occurs or becomes worse in severity after the initiation of study treatment and within max (30 days after last study treatment administration, 90 days after last dose of camrelizumab).

If the CTCAE grade of an AE is missing, it will be considered as grade 3 AE.

The TEAEs which being recorded as being “related with” and “possibly related with” study drug in CRF will be considered as drug related TEAE (TRAE). If the relationship to the study treatment is missing or “cannot be determined”, it will be considered to be related to study treatment.

A high-level summary of the number of subjects with TEAEs will be presented by treatment group, including the number and percentage of subjects with:

- Any TEAEs
- Study drug related TEAEs (TRAEs)
- Chemotherapy related TEAEs (Only for the preoperative phase and whole treatment phase)
- Camrelizumab related TEAEs
- Serious TEAE (TESAE)
- Serious TRAEs
- Serious Chemotherapy related TEAEs
- Serious camrelizumab related TEAEs
- TEAEs with CTCAE grade ≥ 3
- TRAEs with CTCAE grade ≥ 3
- Chemotherapy related TEAEs with CTCAE grade ≥ 3
- Camrelizumab related TEAEs with CTCAE grade ≥ 3

- Immune related TEAEs
- Immune related TEAEs with CTCAE grade ≥ 3
- TEAEs leading to dose interruption in camrelizumab
- TRAEs leading to dose interruption in camrelizumab
- TEAEs leading to interruption/dose reduction in chemotherapy (Only for the preoperative phase and whole treatment phase)
- TRAEs leading to interruption/dose reduction in chemotherapy (Only for the preoperative phase and whole treatment phase)
- TEAEs leading to any study drug permanent discontinuation
- TRAEs leading to any study drug permanent discontinuation
- TEAEs leading to camrelizumab permanent discontinuation
- TRAEs leading to camrelizumab permanent discontinuation
- TEAEs leading to chemotherapy permanent discontinuation (Only for the preoperative phase and whole treatment phase)
- TRAEs leading to chemotherapy permanent discontinuation (Only for the preoperative phase and whole treatment phase)
- TEAEs leading to all study drug permanent discontinuation
- TRAEs leading to all study drug permanent discontinuation
- TEAEs leading to withdrawal from the study
- TRAEs leading to withdrawal from the study
- TEAE leading to death
- TRAEs leading to death
- Immune related TEAEs leading to death

The number and percentage of subjects reporting TEAEs in each treatment group during each study phase (preoperative phase, postoperative phase and whole treatment phase) will be summarized overall, by preferred term (PT), and further be summarized by severity (overall and for CTCAE grade ≥ 3). A subject who reports more than 1 TEAEs with the same PT will only be count once at the corresponding PT level with the most sever CTCAE grade.

Data listing will be provided

8.3.2.1. Treatment-related TEAEs

The number and percentage of subjects reporting TRAEs in each treatment group will be summarized overall, by PT, and further be summarized by severity (overall and for CTCAE grade ≥ 3). Only the occurrence with the highest CTCAE grade and the closest relationship to the study treatment will be counted at the corresponding PT level if a subject who reports more than

1 TEAEs with different CTCAE grade and relationship to the study treatment which could be coded to the same PT.

Data listing of TRAEs will be provided.

8.3.2.2. Serious adverse events

SAE will be summarized in the similar way of TEAE. The number and percentage of subjects reporting SAEs in each treatment group will be summarized overall, by PT.

Data listing of SAE will be provided.

8.3.2.3. TEAE leading to study drug interruption, dose reduction and discontinuation

TEAE leading to study drug interruption/dose reduction and permanently discontinuation will be summarized in the similar way of TEAEs. The number and percentage of subjects reporting aforementioned TEAEs in each treatment group will be summarized overall, by PT, and further be summarized by severity (overall and for CTCAE grade ≥ 3).

Data listing of TEAEs leading to study drug interruption, dose reduction and discontinuation will be provided respectively.

8.3.2.4. Immune related TEAEs

Immune related TEAEs will be summarized in the similar way of TEAEs. The number and percentage of subjects reporting immune related TEAEs in each treatment group will be summarized overall, by PT, and further be summarized by severity (overall and for CTCAE grade ≥ 3).

Data listing of immune related TEAEs will be provided.

8.3.2.5. Death, Other Serious Adverse Events, and Other Significant Adverse Events

The numbers and percentages of TEAEs and TRAEs leading to death will be summarized will be summarized by PT for each treatment group.

8.3.3. Clinical Laboratory Evaluations

The clinical laboratory evaluation system and parameters listed below will be summarized.

Table 5. List of Laboratory Tests.

Hematology	White Blood Cells (WBC), neutrophils (NEU), eosinophils (E), basophils (BAS), lymphocytes (LYM), monocytes (MO), red blood cells (RBC), hemoglobin (Hb), platelets (PLT)
Biochemistry	Total bilirubin (TBIL), direct bilirubin (DBIL), alanine aminotransferase (ALT), aspartate aminotransferase (AST), alkaline phosphatase (AKP), total protein (TP), albumin (ALB), blood urea nitrogen (BUN), creatinine (Cr), creatinine clearance rate (Ccr), fasting blood glucose (GLU), amylase (AMY), potassium (K), sodium (Na), chloride (Cl), calcium (Ca), phosphorus (P)

Urinalysis	Urine protein (U-PRO), urine occult blood (BLD), urine red blood cells (U-RBC), and urine white blood cells (U-WBC)
Coagulation	INR, activated partial thromboplastin time (APTT), prothrombin time (PT), fibrinogen (FIB)
Stool coagulation	Occult blood (OB)
Thyroid function	Serum free triiodothyronine (FT3), free thyroxine (FT4), serum thyroid-stimulating hormone (TSH), triiodothyronine (T3) and thyroxine (T4)
Muscle enzyme spectrum	creatine kinase MB isoenzyme (CK-MB), troponin I (TNI), lactate dehydrogenase (LDH)
Virological tests	Hepatitis B surface antigen (HBsAg), Hepatitis B surface antibody (HBsAb), Hepatitis B e antigen (HBeAg), Hepatitis B e antibody (HBeAb), Hepatitis B core antibody (HBcAb), anti-Hepatitis C virus antibody (anti-HCV Ab), Human Immunodeficiency Virus antibody (HIV Ab)
Other tests	HBV-DNA quantitative test, HCV-RNA quantitative test, pregnancy test

The abnormality for each laboratory parameter will be assessed by investigator. A shift table from baseline to the worst post-baseline assessment according to the investigator's evaluation for each parameter listed below will be presented by treatment group.

If applicable, a summary will be provided based on the baseline and highest NCI-CTCAE grade after baseline for laboratory parameters.

Data listing for the abnormal laboratory tests results will be provided. Also, the pregnancy assessment results as well as the stool blood assessment results will be presented in listings.

8.3.4. Vital Sign

The results and their changes from baseline of each vital sign parameter, including body temperature, pulse, respiratory rate, and blood pressure will be summarized by descriptive statistics for baseline (changes from baseline is not applicable) and each post-baseline scheduled visits by study treatment.

Data listing will be provided for the vital sign results.

8.3.5. ECG and Echocardiogram

The quantitative results and their changes from baseline of each ECG parameter, including heart rate, PR interval, QT interval will be summarized by descriptive statistics for baseline (changes from baseline is not applicable) and each post-baseline scheduled visits by treatment group.

The abnormality of ECG and Echocardiogram will be assessed by investigator. A shift table from baseline to the worst post-baseline assessment according to the investigator's evaluation will be presented for ECG and Echocardiogram by treatment group and study phase.

Data listing will be provided for the ECG and Echocardiogram results.

8.3.6. Eastern Cooperative Oncology Group (ECOG) PS

The analysis of ECOG will be performed based on ITT. The ECOG PS will be summarized as categorical variables for baseline and each post-baseline scheduled visit by treatment group.

Data listing will be provided for the ECOG PS.

8.3.7. Surgical safety relevant endpoints

Surgical complications during the 30 days after surgery or during the hospitalization period, length of hospital stay, reoperation rate, and mortality rates at 30 days post-surgery and 90 days post-surgery will be summarized in SURG. The number and percentage of subjects experienced surgical complications in each group will be summarized overall, by PT, and further be summarized by severity (overall and for different Clavien-Dindo (CD) grades).

Data listing will be provided.

8.4. Patients Reported Outcome (PRO) Analysis

8.4.1. EORTC QLQ-C30

Health-related quality of life (HRQoL) will be assessed by the European Organization for Research and Treatment of Cancer Quality of Life Questionnaire-Core 30 (EORTC QLQ-C30). The EORTC QLQ-C30 consists of 30 questions which assess five functional domains (physical, role, cognitive, emotional and social), global health status/quality of life, disease/treatment related symptoms (fatigue, pain, nausea/vomiting dyspnea, appetite loss, sleep disturbance, constipation and diarrhea) and the perceived financial impact of disease.

The subscales of the EORTC QLQ-C30 will be scored based on the EORTC scoring manual. In summary, each scale will be transformed so that scale scores will range from 0 to 100. The transformation will proceed in two steps.

1. The average of the items contributing subscale will be calculated to compute the raw score (RS) of the scale

   RS=(Q1+Q2+…+Qn)/n

2. A linear transformation will be applied to 'standardize' the raw score

   Function domains: standard score =[1-(RS-1)/R]*100

   Symptom domains and general health status domain: standard score =[(RS-1)/R]*100

   (where R is the full range of scores for each domain or item)

After scores are transformed, high scores will represent higher ("better") levels of functioning and the general health status or a higher ("worse") level of symptoms. Response for the 30 items will be summarized as categorical variable for baseline and each post-baseline scheduled visit by

treatment group. Scale scores will be summarized as continues variable for baseline and each post-baseline scheduled visit by treatment group.

The summary will be performed for the final analysis.

8.4.2. EORTC QLQ-OES18

The QLQ-OES18 includes 18 questions: 6 single item subscales relating to saliva swallowing, choking, dry mouth, taste, coughing, and talking. It also includes 12 items grouped into 4 subscales: dysphagia (3 items), eating (4 items), reflux (2 items), and pain (3 items). The response format was a four-point Likert scale.

Analysis will be performed by using the similar methods in EORTC QLQ-C30.

9. SUMMARY OF CHANGES TO THE STATISTICAL ANALYSES SPECIFIED IN PROTOCOL

Add common odds ratio to the analysis of pCR.

10. REFERENCES

1. Eisenhauer EA, Therasse P, Bogaerts J Schwartz LH, Sargent D, Ford R, et al. New response evaluation criteria in solid tumours: Revised RECIST guideline (version 1.1). Eur J Cancer. 2009;45:228-47.
2. CDE. Guidance for clinical trial data management and report & statistical analysis plan and report.. 2016.
3. CDE. Guidance for clinical trial endpoints of cancer drugs. 2012.
4. ICH E9: Statistical Principles for ClinicalTrials. 1998.
5. ICH E9 (R1): Addendum on estimands and sensitivity analysis in clinical trials. 2019.
6. Maurer, Willi, and Frank Bretz. "Multiple testing in group sequential trials using graphical approaches." Statistics in Biopharmaceutical Research 5.4 (2013): 311-320.

11. APPENDICES

Not applicable.
