# Supplementary material for: Neoadjuvant chemotherapy with or without camrelizumab in resectable esophageal squamous cell carcinoma: the randomized phase 3 ESCORT-NEO/NCCES01 trial
Source: Nat Med. 2024 Jul 2;30(9):2549–57. doi: 10.1038/s41591-024-03064-w (PMC11405280; doi:10.1038/s41591-024-03064-w)
Supplement: Supplementary file 2 — Reporting Summary [file 41591_2024_3064_MOESM2_ESM.pdf]

nature portfolio

Corresponding author(s): Yin Li

Last updated by author(s): May 8, 2024

Reporting Summary

Nature Portfolio wishes to improve the reproducibility of the work that we publish. This form provides structure for consistency and transparency in reporting. For further information on Nature Portfolio policies, see our Editorial Policies and the Editorial Policy Checklist.

Statistics

For all statistical analyses, confirm that the following items are present in the figure legend, table legend, main text, or Methods section.

n/a	Confirmed	
☐	☒	The exact sample size (*n*) for each experimental group/condition, given as a discrete number and unit of measurement
☐	☒	A statement on whether measurements were taken from distinct samples or whether the same sample was measured repeatedly
☐	☒	The statistical test(s) used AND whether they are one- or two-sided<br>*Only common tests should be described solely by name; describe more complex techniques in the Methods section.*
☐	☒	A description of all covariates tested
☐	☒	A description of any assumptions or corrections, such as tests of normality and adjustment for multiple comparisons
☐	☒	A full description of the statistical parameters including central tendency (e.g. means) or other basic estimates (e.g. regression coefficient) AND variation (e.g. standard deviation) or associated estimates of uncertainty (e.g. confidence intervals)
☐	☒	For null hypothesis testing, the test statistic (e.g. F, t, r) with confidence intervals, effect sizes, degrees of freedom and P value noted<br>*Give P values as exact values whenever suitable.*
☒	☐	For Bayesian analysis, information on the choice of priors and Markov chain Monte Carlo settings
☐	☒	For hierarchical and complex designs, identification of the appropriate level for tests and full reporting of outcomes
☐	☒	Estimates of effect sizes (e.g. Cohen's *d*, Pearson's *r*), indicating how they were calculated

Our web collection on statistics for biologists contains articles on many of the points above.

Software and code

Policy information about availability of computer code

Data collection	Data collection was performed using Bioknow EDC.
Data analysis	All statistical analyses were conducted using SAS software version 9.4. The sample size was calculated using NCSS&PASS version 15.0.

For manuscripts utilizing custom algorithms or software that are central to the research but not yet described in published literature, software must be made available to editors and reviewers. We strongly encourage code deposition in a community repository (e.g. GitHub). See the Nature Portfolio guidelines for submitting code & software for further information.

Data

Policy information about availability of data

All manuscripts must include a data availability statement. This statement should provide the following information, where applicable:

- Accession codes, unique identifiers, or web links for publicly available datasets
- A description of any restrictions on data availability
- For clinical datasets or third party data, please ensure that the statement adheres to our policy

Due to intellectual property and confidentiality obligations, individual deidentified participant data that underlie the results reported in this article can be requested 24 months after study completion. Qualified researchers must submit a proposal to the corresponding author at liyin@cicams.ac.cn, outlining the reasons for requesting the data. The leading clinical site and sponsor will review the request to ensure compliance with intellectual property and confidentiality obligations and

will respond within two weeks. A signed data access agreement with the sponsor is required before any data can be shared. The study protocol and statistical analysis plan are available alongside the published article.

Human research participants

Policy information about studies involving human research participants and Sex and Gender in Research.

Reporting on sex and gender	We used sex to report the biological factors for male (n=332) and female (n=59) patients. Gender, as shaped by social and cultural circumstances, was not specifically assessed or reported in the study design. Sex or gender was not considered in the study design. Both male and female patients were eligible. The sex was summarized as part of demographic characteristics in Table 1. The sex of participants was collected according to the identity information provided by the patients. No individual-level data were reported. No prior analysis was sex-based. Subgroup analysis of pCR was performed based on sex (male vs female).
Population characteristics	The median age of all patients was 63 years (range: 44-75), with 84.9% being male. Of the patients, 6 (1.5%) were in clinical stages I (all cT1N1), 100 (25.6%) in stage II, 279 (71.4%) in stage III, and 6 (1.5%) in stage IVA. Tumors were located in the upper, middle, and lower thoracic esophagus for 41 (10.5%), 201 (51.4%), and 149 (38.1%) patients, respectively.
Recruitment	Patients with LA-ESCC were recruited from 24 centers in China using eligibility criteria pre-specified in the study protocol and randomized to the three treatment arms. Key inclusion criteria were histologically confirmed ESCC located in the thoracic esophagus, staged as T1b-3N1-3M0 or T3N0M0 according to American Joint Committee on Cancer (AJCC) Staging 8th edition; candidates deemed suitable for R0 resection; ages between 18 and 75 years; an Eastern Cooperative Oncology Group performance status (ECOG PS) of 0-1; treatment naïve; and adequate organ function. Major exclusion criteria included synchronous or metachronous double malignancies within 5 years, history of autoimmune disease, and the use of immunosuppressive drugs or systemic steroids within 2 weeks prior to enrollment. The detailed inclusion and exclusion criteria are provided in the study protocol.
Ethics oversight	The study protocol was approved by the Ethics Committee of the National Cancer Center/National Clinical Research Center for Cancer/Cancer Hospital, Chinese Academy of Medical Sciences and Peking Union Medical College; Anyang Cancer Hospital; Tangdu Hospital, Air Force Military Medical University; Fujian Provincial Cancer Hospital; Tianjin Medical University Cancer Institute and Hospital; Fujian Medical University Union Hospital; Ruijin Hospital, Shanghai Jiao Tong University School of Medicine; Qilu Hospital of Shandong University; The First Affiliated Hospital of Xi'an Jiaotong University; Harbin Medical University Cancer Hospital; Affiliated Hospital of North Sichuan Medical College; Sichuan Cancer Hospital; West China Hospital, Sichuan University; Zhongshan Hospital, Fudan University; The Affiliated Hospital of Southwest Medical University; Union Hospital, Tongji Medical College, Huazhong University of Science and Technology; The First Affiliated Hospital of Henan University of Science and Technology; The Second Hospital & Clinical Medical School, Lanzhou University; Cancer Hospital of University of Chinese Academy of Sciences, Zhejiang Cancer Hospital; Shanxi Provincial Cancer Hospital; First Affiliated Hospital of Zhengzhou University; Henan Provincial People's Hospital; Shanghai Chest Hospital, Shanghai Jiao Tong University School of Medicine; The Fourth Hospital of Hebei Medical University. All enrolled patients provided written informed consent.

Note that full information on the approval of the study protocol must also be provided in the manuscript.

Field-specific reporting

Please select the one below that is the best fit for your research. If you are not sure, read the appropriate sections before making your selection.

☒ Life sciences ☐ Behavioural & social sciences ☐ Ecological, evolutionary & environmental sciences

For a reference copy of the document with all sections, see nature.com/documents/nr-reporting-summary-flat.pdf

Life sciences study design

All studies must disclose on these points even when the disclosure is negative.

Sample size	Assuming pCR rates of 30% for the Cam+nab-TP group, 25% for the Cam+TP group, and 9% for the TP group, and using a 1:1:1 randomization ratio with an α level set at one-sided 0.005, it was calculated that 111 patients per group would provide at least 93% power to establish the superiority of the Cam+nab-TP group over the TP group, and at least 75% power to demonstrate that the Cam+TP group surpasses the TP group. To accommodate a potential dropout rate of 15%, the study planned to enroll 130 participants in each group. We assumed the median EFS in the TP group to be 30 months. The expected HR for the Cam+nab-TP and Cam+TP groups (combined test groups) compared to the TP group was 0.67. With an initial α set at a one-sided level of 0.02 and with a randomization ratio of 2:1 (Cam+nab-TP and Cam+TP groups vs. TP group), a total of 228 events (141 in the combined test groups and 87 in the TP group) are necessary to achieve at least 80% power to detect the superiority of the test groups. Based on an enrollment period of 36 months, a total study duration of 84 months, and an anticipated dropout rate of 15%, approximately 390 patients were required across the three groups.
Data exclusions	Efficacy results were performed in the ITT population and surgical population. Safety analyses were performed in patients who received study treatment. No data were excluded from the analyses.
Replication	Replication was not applicable to the study (randomized controlled trial).

Randomization	Eligible patients were randomly assigned in a 1:1:1 ratio to either the Cam+nab-TP group, the Cam+TP group, or the TP group using the randomized trial management system. Randomization was stratified according to clinical stage into I/II, III, and IVA.
Blinding	This trial was open-label.

Reporting for specific materials, systems and methods

We require information from authors about some types of materials, experimental systems and methods used in many studies. Here, indicate whether each material, system or method listed is relevant to your study. If you are not sure if a list item applies to your research, read the appropriate section before selecting a response.

Materials & experimental systems

n/a	Involved in the study
☒	☐ Antibodies
☒	☐ Eukaryotic cell lines
☒	☐ Palaeontology and archaeology
☒	☐ Animals and other organisms
☐	☒ Clinical data
☒	☐ Dual use research of concern

Methods

n/a	Involved in the study
☒	☐ ChIP-seq
☒	☐ Flow cytometry
☒	☐ MRI-based neuroimaging

Clinical data

Policy information about clinical studies

All manuscripts should comply with the ICMJE guidelines for publication of clinical research and a completed CONSORT checklist must be included with all submissions.

Clinical trial registration	The study was registered prior to patient enrollment (ChiCTR2000040034).
Study protocol	Submitted with manuscript
Data collection	391 patients were enrolled from 24 hospitals in China between April 28, 2021, and August 7, 2023. Data cutoff date of this analysis was October 8, 2023. All efficacy and safety data were collected at the participating clinical centers, including National Cancer Center/National Clinical Research Center for Cancer/Cancer Hospital, Chinese Academy of Medical Sciences and Peking Union Medical College; Anyang Cancer Hospital; Tangdu Hospital, Air Force Military Medical University; Fujian Provincial Cancer Hospital; Tianjin Medical University Cancer Institute and Hospital; Fujian Medical University Union Hospital; Ruijin Hospital, Shanghai Jiao Tong University School of Medicine; Qilu Hospital of Shandong University; The First Affiliated Hospital of Xi'an Jiaotong University; Harbin Medical University Cancer Hospital; Affiliated Hospital of North Sichuan Medical College; Sichuan Cancer Hospital; West China Hospital, Sichuan University; Zhongshan Hospital, Fudan University; The Affiliated Hospital of Southwest Medical University; Union Hospital, Tongji Medical College, Huazhong University of Science and Technology; The First Affiliated Hospital of Henan University of Science and Technology; The Second Hospital & Clinical Medical School, Lanzhou University; Cancer Hospital of University of Chinese Academy of Sciences, Zhejiang Cancer Hospital; Shanxi Provincial Cancer Hospital; First Affiliated Hospital of Zhengzhou University; Henan Provincial People's Hospital; Shanghai Chest Hospital, Shanghai Jiao Tong University School of Medicine; The Fourth Hospital of Hebei Medical University.
Outcomes	The study's dual primary endpoints included the pCR rate, assessed by the BIRC, and event-free survival (EFS), evaluated by the investigators. pCR is defined as the absence of residual tumor at the primary tumor site (TRG grade 1) and negative lymph nodes. Secondary endpoints include the major pathological response (MPR) rate (defined as less than 10% residual viable tumor cells in the primary tumor) assessed by BIRC, R0 resection rate, post-neoadjuvant pathological staging (ypTNM) according to the AJCC 8th edition, OS, DFS, AEs, and surgical complications.
